# Surpassing the single-atom catalytic activity limit through paired Pt-O-Pt ensemble built from isolated Pt_1_ atoms

**DOI:** 10.1038/s41467-019-11856-9

**Published:** 2019-08-23

**Authors:** Hui Wang, Jin-Xun Liu, Lawrence F. Allard, Sungsik Lee, Jilei Liu, Hang Li, Jianqiang Wang, Jun Wang, Se H. Oh, Wei Li, Maria Flytzani-Stephanopoulos, Meiqing Shen, Bryan R. Goldsmith, Ming Yang

**Affiliations:** 1 School of Chemical Engineering and Technology, Tianjin University, Tianjin, China; 2 Department of Chemical Engineering, University of Michigan, Ann Arbor, MI USA; 3 Catalysis Science and Technology Institute, University of Michigan, Ann Arbor, MI USA; 4 Materials Science and Technology Division, Oak Ridge National Laboratory, Oak Ridge, TN USA; 5 Argonne National Laboratory, X-ray Science Division, Lemont, IL USA; 6 School of Chemical and Biological Engineering, Tufts University, Medford, MA USA; 7 Chemical and Materials Systems Laboratory, General Motors Global Research and Development, Warren, MI USA; 8 State Key Laboratory of Engines, Tianjin University, Tianjin, China; 9 Collaborative Innovation Center of Chemical Science and Engineering, Tianjin, China

**Keywords:** Catalytic mechanisms, Heterogeneous catalysis, Chemical engineering

## Abstract

Despite the maximized metal dispersion offered by single-atom catalysts, further improvement of intrinsic activity can be hindered by the lack of neighboring metal atoms in these systems. Here we report the use of isolated Pt_1_ atoms on ceria as “seeds” to develop a Pt-O-Pt ensemble, which is well-represented by a Pt_8_O_14_ model cluster that retains 100% metal dispersion. The Pt atom in the ensemble is 100–1000 times more active than their single-atom Pt_1_/CeO_2_ parent in catalyzing the low-temperature CO oxidation under oxygen-rich conditions. Rather than the Pt-O-Ce interfacial catalysis, the stable catalytic unit is the Pt-O-Pt site itself without participation of oxygen from the 10–30 nm-size ceria support. Similar Pt-O-Pt sites can be built on various ceria and even alumina, distinguishable by facile activation of oxygen through the paired Pt-O-Pt atoms. Extending this design to other reaction systems is a likely outcome of the findings reported here.

## Abstract (web summary)

Single-atom metal catalysts offer maximized material efficiency, but there is large room to improve the intrinsic activity per metal atom for many reactions. Here, the authors demonstrate that the solution for CO oxidation is to tackle the issue of lacking neighboring Pt atoms in the single-atom Pt1/CeO2 system.

## Introduction

An ideal supported metal catalyst will simultaneously maximize dispersion of the metal and display optimal intrinsic activity per metal atom. Recently, advanced techniques for synthesizing many heterogeneous catalysts as single atoms have addressed the former issue. Single-atom catalysis dramatically reduces the usage requirements of expensive and rare metals by stabilizing the supported metal atoms in a fully dispersed state as isolated bonded species that serve as active sites^1–6^. A general question regarding single-atom metal catalysts, despite the nearly 100% material efficiency of the supported metals, is whether a catalytic center designed in the form of one metal atom substituted or anchored on a support represents the optimal structure to deliver the highest intrinsic catalytic activity. Previous work answered this question for platinum and gold catalysts for the water–gas shift reaction, where the optimal catalytic center is the single-atom Pt_1_(or Au_1_)-O(OH)_*x*_ species on a variety of catalyst supports^4–6^. Nonetheless, for other important reactions such as the low-temperature CO oxidation, these configurations as isolated atomic active sites may lack neighboring metal centers and the reactivities associated with the latter. This fundamental question remains unanswered and industrial needs for more active catalysts await.

The catalytic oxidation of CO to CO_2_ involves classic molecular rearrangements with oxygen intermediates that make it an attractive probe reaction in catalytic systems to gain better mechanistic understanding, such as the identity of metal catalytic centers and the importance of metal–support interaction. The low-temperature CO oxidation is also important in the purification of vehicle emissions. To meet latest fuel-efficient engine designs and to reduce vehicle exhaust emissions, platinum group metals (PGMs) dispersed on ceria supports are needed to be much more active in eliminating CO emissions below 150 °C during the engine cold start^7^. A group of Pt single-atom catalysts using CeO_2_^8,9^, Al_2_O_3_^10,11^, and KLTL zeolite^12^ supports were developed and probed for CO oxidation. Compared with the conventional Pt nanoparticles where most of the metal atoms are buried inside the particle bulk without catalyzing the surface reaction, these single-atom catalysts certainly facilitate the full utilization of scarce platinum metal^4–6,8,9^. However, the properties of the Pt_1_ may be suboptimal for certain reactions. Indeed, a closer examination of the activity per Pt atom shows that such Pt_1_ catalysts are often similar to (or even worse than^3^) the conventional Pt nanoparticles and clusters under comparable reaction conditions and catalyst formulations (Supplementary Table 1). In the context of oxygen-rich reaction conditions (oxygen in excess to fully oxidize all the reductants), which reflects the implementation of emerging fuel saving technologies such as lean-burn engines, hybrid powertrains, and dynamic fuel management, the known benefit of creating oxygen vacancies on ceria surfaces to promote CO oxidation under near stoichiometric oxygen concentrations^13–15^ cannot be sustained because the surface oxygen vacancies associated with Ce(III) heal in seconds^16,17^. Consequently, the natural question arises whether a properly paired multi-atom catalytic site (ensemble of the single-atom M_1_-O_*x*_ species) will increase the catalytic performance over Pt_1_/CeO_2_ under oxygen-rich conditions, and how we can build such a site with an appreciable loading amount on a given support surface. In this work, by extending the concept of isolated atoms to the paired ensembles, we show that the paired Pt-O-Pt catalytic units can achieve higher activity through an oxygen migration mechanism.

Here we report a general approach—reassembling isolated Pt_1_ atoms as the precursor to create a one-layer multi-atom oxo site (Pt-O-Pt) while keeping ~100% Pt utilization. We use a variety of experimental and computational techniques (grand canonical Monte Carlo (GCMC) simulations combined with density functional theory (DFT) calculations (GCMC-DFT)^18^ to elucidate the catalyst active site structure and the CO oxidation reaction mechanism that is responsible for the dramatically enhanced reactivity of this multi-atom oxo site. The Pt-O-Pt ensemble is shown to be the base unit in the high-performance catalyst, where the well-known Pt-CeO_2_ metal–support interfacial catalysis no longer ranks as the favorable reaction path for this highly active low-temperature CO oxidation catalyst under oxygen-rich reaction conditions.

## Results

### Dramatic change of catalytic performance

We began this work by synthesizing a variety of single-atom Pt_1_/CeO_2_ to serve as the baseline for catalytic performance. Next, the isolated Pt atoms in the Pt_1_/CeO_2_ were used as seeds to generate a much more active Pt site through a facile activation treatment, where a mild H_2_ reduction was followed with a CO plus O_2_ treatment to trigger the restructuring of the platinum (see optimization of the activation treatment and the stable, high reaction rates in Supplementary Figs. 1–3. The optimized samples are labeled as Pt-O-Pt/CeO_2_, see Table 1). The potential alternative activity contributors, such as creating persistent oxygen vacancies and additional –OH species on the catalyst surface during the activation treatment, have been excluded (Supplementary Figs. 4 and 5).

**Table 1:** Key metrics of the Pt_1_/CeO_2_ and the Pt-O-Pt/CeO_2_ catalysts

Samples	Pt material efficiency (%)^a^	Active sites	Relative activity	*E*_app_ (kJ/mol)	Rate-determining step
Pt_1_/CeO_2_	~100	Isolated Pt_1_ atoms embedded in CeO_2_ surface by substitution of surface Ce atoms	1×^b^	86 ± 3 (Exp.) 78 (The.)	O_2_ dissociation at Pt_1_/CeO_2_; CeO_2_ is involved in the catalytic cycle
Pt-O-Pt /CeO_2_	~100	Pt-O-Pt ensemble. Pt atoms are separated but bridged by four oxygen atoms	~10^2^ – 10^3^ higher^c^	40 ± 2 (Exp.) 54 (The.)	Oxygen atom migration at the Pt-O-Pt ensemble; CeO_2_ is not involved in the catalytic cycle

*HAADF-STEM,* high-angle annular dark-field scanning transmission electron microscopy, *Exp.*, experiment measured, *The.*, theory predicted

^a^Based on spectroscopic observations from HAADF-STEM images and the chemical titrations of CO chemisorption

^b^The catalyst activity is similar with many recently reported single-atom Pt_1_/CeO_2_ catalysts (see Supplementary Table 1)

^c^Reaction in the window of 80–150 °C

**Fig. 1:**
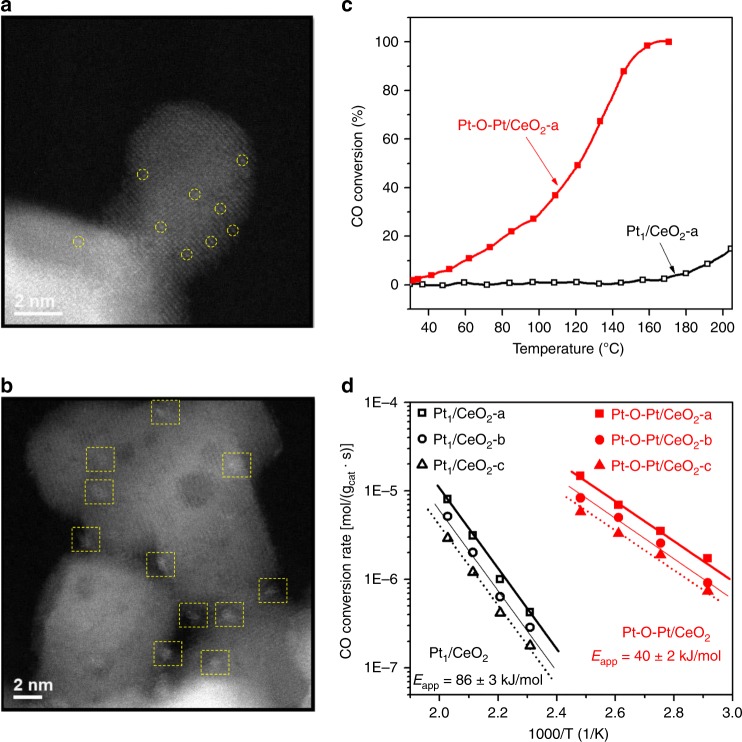
Direct measurements of the Pt_1_/CeO_2_ and Pt-O-Pt/CeO_2_ catalysts. **a**, **b** Aberration-corrected high-angle annular dark-field scanning transmission electron microscopy (HAADF-STEM) images for the Pt_1_/CeO_2_-a and Pt-O-Pt/CeO_2_-a catalysts (shown images were recorded at ×10 M and ×6 M original magnifications, respectively). Yellow circles and squares are used to highlight the single-atom Pt_1_ and the Pt-O-Pt ensemble, respectively. **c** CO oxidation light-off performance ([CO] = 1000 ppm, [O_2_] = 5%, balanced with N_2_ at a contact time of 2,400,000 mL/g_cat_//h). **d** Arrhenius-type plot of CO oxidation rates at different temperatures with apparent activation energies (*E*_app_) shown

Aberration-corrected high-angle annular dark-field scanning transmission electron microscopy (HAADF-STEM) images show that the single Pt atoms are the majority species in the various Pt_1_/CeO_2_ samples prepared in this work (see Fig. 1a and Supplementary Figs. 6–8 for Pt_1_/CeO_2_-a, Pt_1_/CeO_2_-b, and Pt_1_/CeO_2_-c samples), where Pt loadings of 0.27, 0.16, and 0.11 wt.% and ceria supports with diverse amounts of reducible oxygen species were used (Supplementary Figs. 9 and 10). A few pseudo-clusters of platinum might be found in these single-atom catalyst samples, where these pseudo-clusters are composed of several nearby single-atom Pt_1_ species embedded in the cerium columns. In the activated catalysts, namely the Pt-O-Pt/CeO_2_ samples, the Pt atoms are fully transformed into another structure with a narrow size distribution of 1 ± 0.1 nm on the ceria surface (see Fig. 1b and Supplementary Figs. 11–13 for Pt-O-Pt/CeO_2_-a, Pt-O-Pt/CeO_2_-b, and Pt-O-Pt/CeO_2_-c samples). We measure the Pt dispersion of Pt-O-Pt/CeO_2_ samples to be nearly 100% (Table 1). Additional STEM images and discussion of the representative Pt_1_/CeO_2_-a and Pt-O-Pt/CeO_2_-a samples can be found in Supplementary Figs. 14 and 15 and their accompanying text. None of the crystal patterns for platinum metal or oxides were detected in the Pt-O-Pt/CeO_2_ catalysts by either STEM imaging or X-ray diffraction (Fig. 1b and Supplementary Figs. 11–13, 15, and 16), meaning that only the highly dispersed platinum species reside on the predominant CeO_2_(111) surfaces (Supplementary Fig. 17) with ceria nanoparticle sizes of 10–30 nm. There are some rounded edges, steps, and kinks on these rather typical industrial CeO_2_ support particles, but no clear evidence of these locations as the preferred anchoring sites for the platinum species is found (Supplementary Fig. 18). Therefore, we selected CeO_2_(111) to model the stable geometry and CO oxidation reaction path for Pt_1_/CeO_2_ and Pt-O-Pt/CeO_2_.

Despite the apparently low conversion values on the Pt_1_/CeO_2_ catalysts (Fig.1c and Supplementary Fig. 19), which are due to the high gas flow rate compared to catalyst weight, the absolute (or intrinsic) activities of our single-atom Pt for low-temperature CO oxidation per Pt site (turnover frequencies, TOFs) are within the same order of magnitude as the activities reported recently for other single-atom Pt catalysts^8–12,19–24^, particularly when reducible oxide supports such as titania and ceria were used (Supplementary Table 1). Under the same reaction conditions, however, the Pt-O-Pt/CeO_2_ catalysts have 2–3 orders of magnitude higher intrinsic activity than their Pt_1_/CeO_2_ counterparts from 80 to 150 °C. The reaction rates on the Pt_1_/CeO_2_-a catalyst are 1.7 × 10^–9^ and 2.2 × 10^–7^ mol CO_2_/(g_cat_·s) at 80 and 150 °C, respectively. In contrast, at the same total platinum loading, the reaction rates on the Pt-O-Pt/CeO_2_-a catalyst are 2.6 × 10^−6^ and 2.5 × 10^−5^ mol/(g_cat_·s) at 80 and 150 °C. Kinetic measurements (Fig. 1d) further reveal that the Pt-O-Pt/CeO_2_ and Pt_1_/CeO_2_ catalysts are also distinguishable from differences between their catalytic centers and reaction mechanisms. Specifically, the CO oxidation reaction catalyzed by the Pt-O-Pt/CeO_2_ catalysts has a smaller measured apparent activation energy (*E*_app_ = 40 ± 2 kJ/mol) compared with the Pt_1_/CeO_2_ catalysts (*E*_app_ = 86 ± 3 kJ/mol).

In agreement with earlier studies on metal nanoparticle catalysts^25,26^, recent reports have confirmed the benefit of H_2_O and its dissociated –OH species for promoting CO oxidation on Pt_1_/CeO_2_ catalysts^9,19^. In our case, an activity improvement for both Pt-O-Pt/CeO_2_ and Pt_1_/CeO_2_ catalysts is observed after adding 3% H_2_O into the CO oxidation feed stream (Supplementary Fig. 20), but the large activity gap between the Pt-O-Pt/CeO_2_ and Pt_1_/CeO_2_ samples remained. This result proves that the beneficial H_2_O/–OH-rich environment^9,19,25,26^ does not diminish the superior activity of the Pt-O-Pt/CeO_2_ over its Pt_1_/CeO_2_ counterpart. Despite the further complication of reaction routes due to the water-containing experiment, these data demonstrated that the relative activity of the Pt-O-Pt structure compared with Pt_1_/CeO_2_ will not be negated even in the presence of water and –OH-enriched environment. The activated Pt-O-Pt/CeO_2_-a sample remains similarly active even after being hydrothermally aged at 750 °C for 20 h (Supplementary Fig. 20). Further evidence of the generalizability of this synthetic approach to effectively construct the active Pt-O-Pt catalytic center is shown on two types of commercial platinum–ceria catalysts (Supplementary Figs. 21 and 22).

### Atomic-level structural analyses

**Fig. 2:**
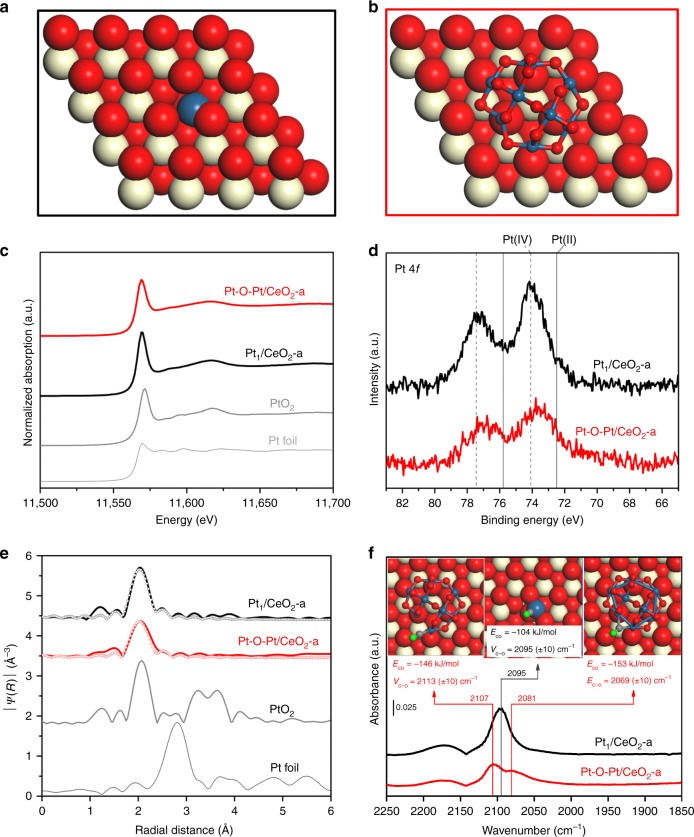
The density functional theory (DFT)-optimized Pt structures and the material characterization results. **a**, **b** DFT-optimized structure of representative single-atom Pt in the Pt_1_/CeO_2_ sample and Pt_8_O_14_ in the Pt-O-Pt/CeO_2_ sample found by grand canonical Monte Carlo-DFT (GCMC-DFT). **c** Normalized Pt L_3_ edge x-ray absorption near-edge structure (XANES) spectra and **d** Pt 4*f* x-ray photoelectron spectroscopy (XPS) spectra of the Pt_1_/CeO_2_-a and the Pt-O-Pt/CeO_2_-a catalysts. **e** Fourier transform of extended X-ray absorption fine structure (EXAFS) spectra of Pt L_3_ edge (phase corrected) for the Pt_1_/CeO_2_-a and Pt-O-Pt/CeO_2_-a catalysts. The first shell Pt-Pt coordination is not observed in the Pt_1_/CeO_2_ and Pt-O-Pt/CeO_2_ catalysts. The gray and red open circles are fitted curves for the Pt_1_/CeO_2_ and Pt-O-Pt/CeO_2_ catalysts, respectively. The PtO_2_ standard is in the β-phase. **f** In situ diffuse reflectance infrared Fourier-transform spectroscopy (DRIFTS) under the CO oxidation conditions for the Pt_1_/CeO_2_-a and Pt-O-Pt/CeO_2_-a catalysts. DFT-predicted CO adsorption modes are shown inset for Pt_1_/CeO_2 − *x*_ and Pt_8_O_13_/CeO_2_. Color legend of atoms: Ce = beige; Pt = blue; C = gray; O (in CeO_2_ and Pt_8_O_13_) = red; O (in CO) = green

Our DFT calculations identified the stable Pt structures for the Pt_1_/CeO_2_ and Pt-O-Pt/CeO_2_ catalysts. We find that the isolated Pt_1_ atom prefers to substitute the Ce atom rather than anchor on the CeO_2_(111) surface (the dominant facet on which ~70% counts of the experimental Pt_1_ and ~1 nm Pt-O-Pt species were anchored, Supplementary Fig. 17). This Pt_1_ anchoring site was proposed in a prior experimental study^27^. The Pt_1_ anchored on top of the CeO_2_(111) is thermodynamically unstable due to a highly unsaturated coordination environment (Supplementary Fig. 23a). In the identified Pt_1_/CeO_2_ structure (Fig. 2a and Supplementary Figs. 23b and 24), the Pt_1_ substitutes the Ce atom on the ceria surface and is surrounded by up to six nearby oxygen atoms. In line with the recent findings from surface science and DFT calculations studies^28,29^, we noticed that four of the oxygen atoms prefer to bind directly to the Pt center to form a square-planar Pt_1_-O_4_ structure, which is the starting structure of the Pt_1_/CeO_2_ catalysts used here to model the CO oxidation reaction.

Computationally intensive GCMC-DFT simulations were performed to search the structure and composition of Pt_8_/CeO_2_ in the presence of oxygen ($p_{\mathrm{O}_{2}}$ = 0.05 atm) at 350 K, which is consistent with the experimental reaction conditions. The computationally tractable Pt_8_ cluster was selected here because the experimental Pt-O-Pt/CeO_2_ sample has a similar platinum diameter of ~1 nm on the CeO_2_(111) surfaces (Supplementary Fig. 25a). When exposed to an oxygen atmosphere as in our reaction tests, the Pt_8_/CeO_2_ rearranges into Pt_8_O_14_ (~0.92 nm), which contains solely Pt-O-Pt as the catalytic base unit and is only one layer thick (Fig. 2b and Supplementary Fig. 26b). Our GCMC simulation is similar with the experimental activation procedure in that the reduced Pt_*x*_ clusters, formed by assembling Pt_1_ species under the first step of H_2_ treatment, can be oxidized to the Pt-O-Pt ensemble by the introduction of an O_2_-rich environment. The Pt_8_O_14_ cluster model identified by the GCMC algorithm is regarded as a representative structure of our experimental system, as in reality some experimental heterogeneity does exist. Nevertheless, as we show below it captures many of the salient features of this rather clean experimental catalytic system, as indicated by the overall agreement of experimental observations and computational predictions.

Since our synthesis of the Pt_1_/CeO_2_ and Pt-O-Pt/CeO_2_ catalysts yields almost exclusively either single-atom Pt_1_ species or the ~1 nm Pt-O-Pt structure on the different ceria supports, the representative Pt_8_O_14_ structure predicted by GCMC-DFT can be vetted by our experimental analysis. The x-ray absorption near-edge structure (XANES) and the x-ray photoelectron spectroscopy (XPS) results (Fig. 2c–d and Supplementary Figs. 26 and 27) confirm the cationic nature of the Pt atoms in both groups of catalysts. The Pt chemical valence in Pt_1_/CeO_2_ is Pt(IV) and is slightly more positive than the Pt in the Pt-O-Pt/CeO_2_ samples. Bader charge analysis of the predicted catalyst structures shows the same trend (Supplementary Table 2). Extended X-ray absorption fine structure (EXAFS) measurements (Fig. 2e, Supplementary Fig. 28, and Supplementary Table 3) show the typical exclusive Pt-O coordination environment (up to six nearby O atoms) for Pt_1_/CeO_2_. For the Pt-O-Pt/CeO_2_ samples, unlike the prevalent three-dimensional platinum (oxide) clusters that possess more than one Pt layer and the resulting Pt-Pt coordination from EXAFS in a radial distance between 2.5 and 3.0 Å^30,31^, the Pt atoms of Pt-O-Pt/CeO_2_ are only bridged by oxygen in its first shell coordination, making these Pt atoms share a high level of similarity to the isolated Pt_1_ atom in terms of the Pt-O local structures (see XPS and XAS results in Fig. 2, Supplementary Figs. 26–28, and Supplementary Table 3). Consistent with the EXAFS measurements, the GCMC-predicted Pt_8_O_14_ structure is composed of eight fully separated Pt cations, each bound to four nearby oxygen atoms with the average Pt-O distance of 1.99 Å. The lack of long-distance Pt-Pt and second shell Pt-O information for the Pt-O-Pt/CeO_2_ catalysts at higher radial distances (*R* > 3 Å) is the result of experimental signal dampening by the one-layer platinum structure, dilute platinum concentrations, and inherent signal attenuation in EXAFS analyses. These factors are discussed in the Supplementary Information accompanying Supplementary Fig. 29.

Diffuse reflectance infrared Fourier-transform spectroscopy (DRIFTS) studies under the CO oxidation conditions (Fig. 2f and Supplementary Fig. 30) reveal one dominant vibrational mode at 2095 cm^−1^ for the Pt_1_/CeO_2_ samples, as reported in the literature^9^. A Pt_1_ anchored on CeO_2_(111) can be ruled out because of its highly endothermic formation energy (Supplementary Fig. 23a) and its strong CO adsorption (*E*_CO_ = −313 kJ/mol) with a calculated CO wavenumber of 2070 cm^−1^, which is not observed in our DRIFTS measurement. The structure of Pt_1_ substituted in the CeO_2_(111) surface is more stable and the calculated CO wavenumber of 2095 cm^−1^ on Pt_1_/CeO_2 − *x*_(111) with one oxygen vacancy nearby is in line with our experimental measurement. The two sets of experimentally measured CO adsorption bands centered at 2107 and 2081 cm^−1^ for the Pt-O-Pt/CeO_2_ samples correspond to CO chemisorption at the top and bridge sites of an undercoordinated Pt-O-Pt ensemble, according to our representative Pt_8_O_13_/CeO_2_(111) model system (identified after examination of all possible CO adsorption sites on Pt_8_O_*x*_ (*x* = 13−14) involved in the CO oxidation cycle). On Pt_8_O_13_/CeO_2_(111), CO chemisorption is −146 and −153 kJ/mol at the top and bridge site, respectively (Fig. 2f and Supplementary Figs. 30 and 31). Adsorbed CO can interchange between the bridge site and the top site. This evidence does not necessarily corroborate that these CO adsorption modes must belong to the reaction intermediates of the most energy-favored reaction pathways. Also, although the calculated frequencies of adsorbed CO on Pt_8_O_13_/CeO_2_ have similar values compared with the experimental peak centers, we cannot exclude the other possible CO adsorption modes on any heterogeneous PtO_*x*_ species in the Pt-O-Pt/CeO_2_ catalyst. This limitation is also evidenced and discussed from the perspective of experimental DRIFTS results (Supplementary Fig. 30). We emphasize that the C-O vibrational frequency alone cannot be used to definitively identify the Pt adsorption site^32^, and a combined analysis approach of HAADF-STEM, XPS, XANES, and EXAFS must be used to corroborate the theory predicted representative structure, as these characterization results together set the context of platinum species size, chemical valence, and coordination environment.

### Identification of oxygen migration reaction mechanism

**Fig. 3:**
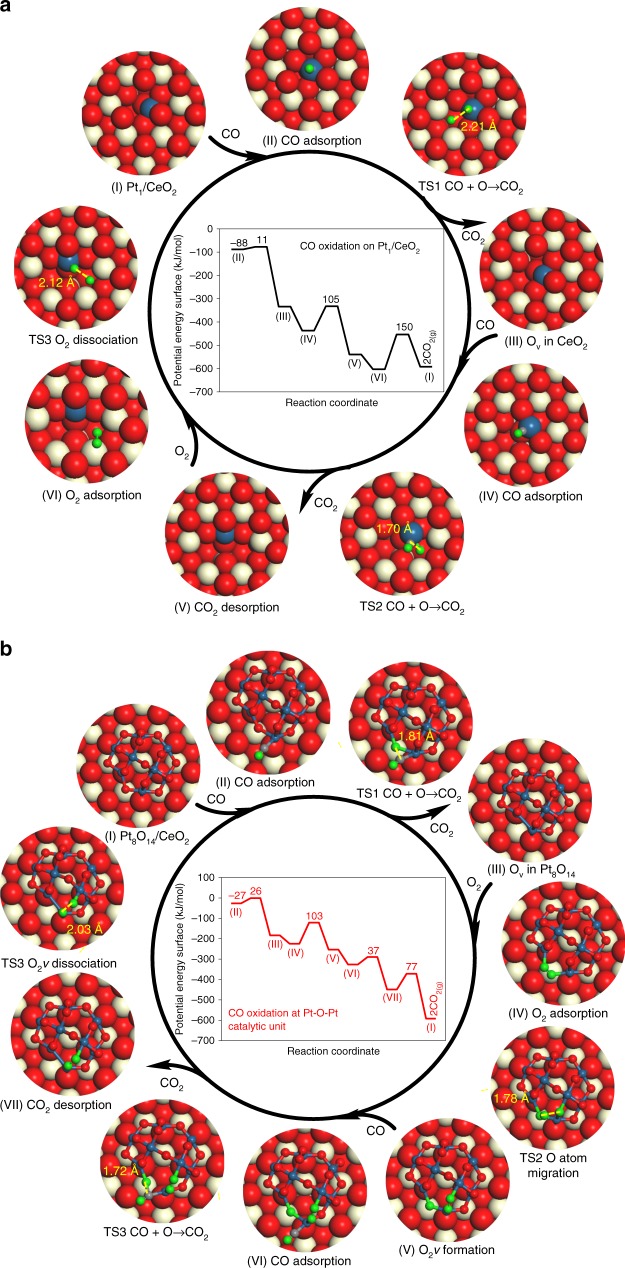
Potential energy diagrams and configurations for CO oxidation cycle. CO oxidation proceeds on **a** the Pt_1_/CeO_2_ and **b** at the Pt-O-Pt catalytic unit in Pt_8_O_14_/CeO_2_. CO adsorption energies and reaction barriers are indicated in kJ/mol in the potential energy diagram. The bond distance between the two fragments at the transition state (TS) is given in angstrom (Å) in the configurations of CO oxidation. Beige, red, and blue spheres are Ce, O, and Pt atoms, respectively. The small gray and green spheres are C and O atoms involved in CO oxidation. Corresponding energetics are given in Supplementary Table 4

**Fig. 4:**
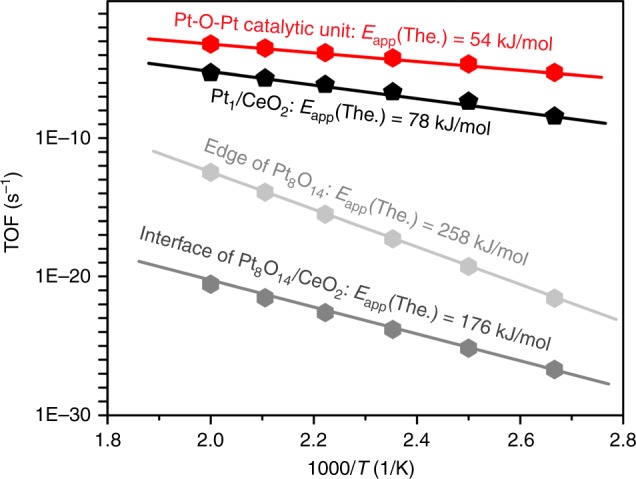
Microkinetic simulations for various CO oxidation routes on the Pt_1_/CeO_2_ and Pt_8_O_14_/CeO_2_ structures. The predicted apparent activation barriers, *E*_app_ (The., theory predicted), are indicated

To understand how the Pt_1_/CeO_2_ and Pt-O-Pt/CeO_2_ catalysts catalyze CO oxidation so differently, DFT calculations combined with mean-field microkinetic simulations were conducted to study the CO oxidation mechanism (the partial pressure for CO and O_2_ are 0.001 and 0.05 bar, respectively, consistent with our reaction studies). The schemes for the CO oxidation cycles and geometric and energetic information are shown in Fig. 3, Supplementary Figs. 32, 33, and Supplementary Table 4. On Pt_1_/CeO_2_(111) and Pt_8_O_14_/CeO_2_(111), the preferred pathway involves adsorbed CO on the Pt site during the CO oxidation cycle due to stronger adsorption of CO compared with O_2_ at the same Pt site (Supplementary Table 5). CO oxidation is predicted to follow the Mars–van Krevelen (MvK) mechanism^33–35^ at the square-planar Pt_1_-O_4_ unit in Pt_1_/CeO_2_ (Fig. 3a and Supplementary Table 4), where adsorbed CO on the Pt atom (CO_Pt_) reacts with surface lattice oxygen (O_Ce_) in CeO_2_ with a moderate activation barrier (*E*_a_ <105 kJ/mol). O_2_ dissociation (*E*_a_ = 150 kJ/mol) will heal the oxygen vacancy sites to complete the catalytic cycle on Pt_1_/CeO_2_. Based on microkinetic simulations, the predicted apparent activation energy (*E*_app_) on Pt_1_/CeO_2_ is 78 kJ/mol (Fig. 4), which is in close agreement (within typical DFT errors of ±15 kJ/mol) with the experimentally measured apparent activation energy of 86 ± 3 kJ/mol. Degree of rate control analysis shows that O_2_ dissociation is the rate-determining step (RDS) for CO oxidation on Pt_1_/CeO_2_ (Supplementary Figs. 34a, 35a).

For the Pt-O-Pt/CeO_2_ samples, we have considered three potential catalytic cycles in the Pt_8_O_14_/CeO_2_ system, namely: (1) at the platinum–ceria interface of Pt_8_O_14_/CeO_2_, (2) at the edge of Pt_8_O_14_, and (3) at the Pt-O-Pt site in Pt_8_O_14_. The first step for CO oxidation in scenario 1 is the removal of one oxygen atom in Pt_8_O_14_ by CO_Pt_ with an activation barrier of 26 kJ/mol (Supplementary Fig. 32 and Supplementary Table 4). Afterwards, CO oxidation may take place at the interface of Pt_8_O_14_/CeO_2_, obeying the MvK mechanism and involving O_Ce_ removal and O_2_ dissociation. The reaction of CO_Pt_ with O_Ce_ to form CO_2_ has the highest activation barrier for CO oxidation (*E*_a_ = 151 kJ/mol), followed by O_2_ dissociation (*E*_a_ = 72 kJ/mol). The predicted apparent activation energy is 176 kJ/mol at the metal–support interface of Pt_8_O_14_/CeO_2_, which results in a much too low CO oxidation rate compared with the experimental measurements (Fig. 4). The lower activity of the Pt_8_O_14_/CeO_2_ interface compared with Pt_1_/CeO_2_ originates from the weak CO adsorption on Pt_8_O_14_/CeO_2_ (*E*_CO_ = −27 kJ/mol), resulting in low CO coverage (Supplementary Figs. 34b and 35b). Although the platinum–ceria interface is accepted as the catalytic center for CO oxidation catalyzed by either single-atom Pt_1_/CeO_2_ catalysts or classic nanoparticles/clusters supported on ceria^36,37^, the active site at the interface of our Pt_8_O_14_/CeO_2_ system cannot rationalize the observed high activity of the Pt-O-Pt/CeO_2_ catalysts under these oxygen-rich conditions at low temperatures.

Alternatively, we probed the possible reaction route where CO oxidation proceeds solely on the Pt_8_O_14_ and the CeO_2_ support is a spectator (scenarios 2 and 3). At the Pt_8_O_14_ edge atoms, O_2_ physisorbs ($E_{{\mathrm{O}}_{2}}$ = −1 kJ/mol) after CO adsorption (Supplementary Fig. 33 and Supplementary Table 4). The RDS for CO oxidation is CO_Pt_ + O_2_ → CO_2_ + O_Pt_ with an activation barrier of 117 kJ/mol (Supplementary Figs. 34c, 35c and Supplementary Table 4). Consequently, the CO oxidation rate is still too slow at the Pt_8_O_14_ edge atoms with an apparent activation energy of 258 kJ/mol (Fig. 4) due to the weak adsorption of O_2_ and high barrier for CO_Pt_ reacting with O_2_. Surprisingly, this work finds that the Pt-O-Pt ensemble in the Pt_8_O_14_ (scenario 3) can facilitate an O migration mechanism to rapidly catalyze CO oxidation (Fig. 3b and Supplementary Table 4). One O atom from the adsorbed O_2_ can migrate to a neighboring O atom in the Pt_8_O_14_ to form O_2_*v* with an *E*_a_ = 103 kJ/mol, followed by O_Pt_ removal and O_2_*v* dissociation. The activation barriers for the O_Pt_ atom reacting with CO_Pt_ and O_2_*v* dissociation are predicted to be at least 26 kJ/mol lower than the O atom migration step; thus, O migration is the RDS for this mechanism (Supplementary Figs. 34d, 35d and Supplementary Table 4). The microkinetic simulations predict *E*_app_ for the Pt-O-Pt/CeO_2_ catalysts of 54 kJ/mol, which is close to our experimentally measured result of 40 ± 2 kJ/mol (Fig. 4).

To probe the impact of other minor CeO_2_ morphologies observed in experimental work (Supplementary Figs. 17 and 18) on the activity of Pt_8_O_14_, the rate-determining CO oxidation reaction step involving CO reacting with lattice oxygen is studied on Pt_8_O_14_/CeO_2_(110) and Pt_8_O_14_/CeO_2_(100). The representative Pt_8_O_14_ structure on CeO_2_(111) surface searched by GCMC simulations is deposited on (110) and (100) facets with each Pt atom binding four oxygen atoms according to our XPS and EXAFS data. DFT calculations (Supplementary Fig. 36) show that CO oxidation at the interface of Pt_8_O_14_/CeO_2_(110) and Pt_8_O_14_/CeO_2_(100) is unfeasible due to a prohibitively high CO oxidation reaction barrier. Therefore, our analyses of platinum species on each of the common ceria surface facets allow us to infer that CO oxidation preferentially occurs on Pt-O-Pt ensemble rather than at the interface of Pt_8_O_14_/CeO_2_ for (111), (110), and (100) facets. The measured reaction orders with respect to CO and O_2_ are −0.2 and 0.4, respectively, for Pt_1_/CeO_2_, and 0.3 and ~0, respectively, for Pt-O-Pt/CeO_2_. These reaction orders corroborate the proposed reaction mechanism (see detailed derivation for reaction orders in Supplementary discussion below Supplementary Fig. 37).

**Fig. 5:**
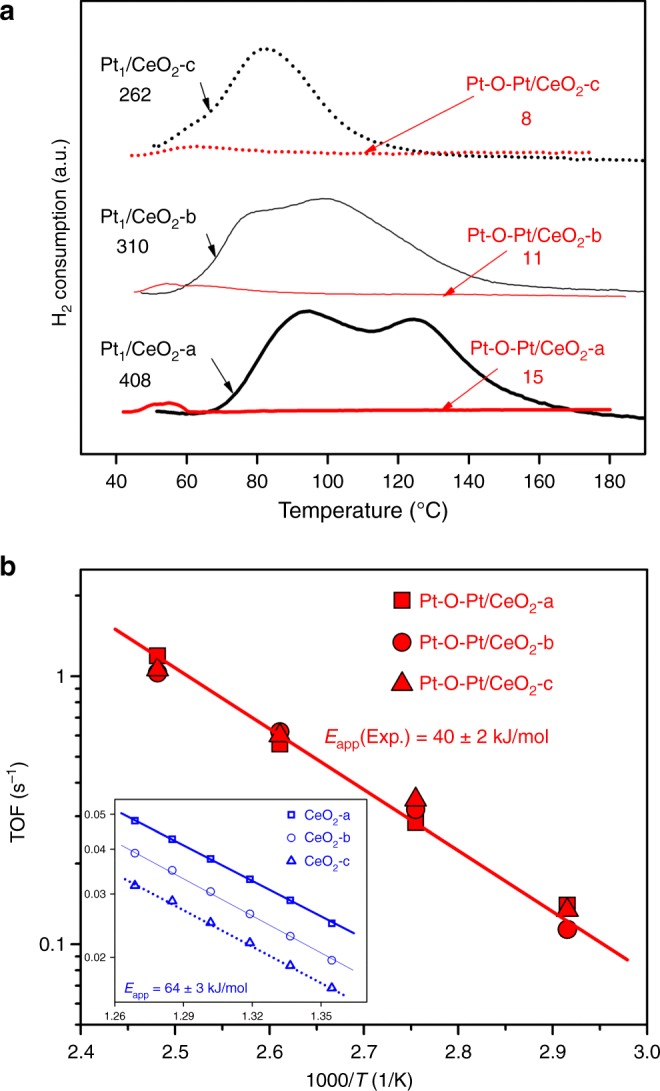
Catalyst surface [O] reducibility and site-specific turnover frequencies (TOFs) for CO oxidation reaction. **a** H_2_ temperature programmed reduction (TPR) profiles and integrated amounts of reducible [O] for the Pt_1_/CeO_2_ and Pt-O-Pt/CeO_2_ catalysts. The labeled values in **a** are the integrated amount of reducible oxygen with the default unit of “µmol [O]/g_cat_.” **b** Identical TOFs were found for the Pt-O-Pt/CeO_2_ catalysts and different TOFs were found for the Pt-free ceria supports (inset)

The indirect catalytic role of ceria predicted in this proposed catalytic route corroborates our experimental findings. As shown by the H_2_ temperature programmed reduction (TPR) results (Fig. 5a) of the reaction-spent catalysts, the more abundant reducible oxygen species from the platinum–ceria interface in the Pt_1_/CeO_2_ catalysts did not count towards the superior catalytic performance of the Pt-O-Pt/CeO_2_ catalysts. The ceria supports are essentially identical for the Pt_1_ and Pt-O-Pt groups of reaction-spent catalysts according to Ce3*d* and O1*s* XPS spectra (Supplementary Fig. 4). Without the presence of the supported platinum, the [O] reduction in the ceria lattice by H_2_ will not take place until above 200 °C (Supplementary Fig. 9b). We attribute the major peak in the temperature range of 60–100 °C to the immediate [O] depletion at the six nearest oxygen atoms in the Pt_1_-O-Ce unit. The next H_2_ consumption peak in the temperature range of 100–160 °C is related to the further depletion of the ceria lattice oxygen that can migrate to the Pt_1_-CeO_2_ interfaces. In contrast, the Pt-O-Pt/CeO_2_ catalysts do not display active [O] supply from the ceria to initiate the low-temperature oxidation. A trace amount of [O] reduction takes place in the temperature range of 40–60 °C for all three Pt-O-Pt/CeO_2_ catalysts. To examine the impact of the ceria supports, the three types of ceria, a, b, and c, were first probed with the CO oxidation reaction as bare supports. The calculated TOFs per Ce site are quite different among the Pt-free ceria materials (Fig. 5b, inset), as they have different capacities in releasing atomic oxygen species to oxidize gas molecules such as H_2_ and CO (Supplementary Figs. 9 and 10). In contrast, the same TOFs can be obtained when we calculate the TOFs per Pt atom from the various Pt-O-Pt/CeO_2_ catalysts (Fig. 5b). This finding shows that the highly active Pt-O-Pt catalytic unit overrides the influence of the different oxygen-supply capabilities from ceria in catalyzing the low-temperature CO oxidation reaction, and the uniformity of the catalytic units created by our activation procedure is remarkable.

We also probed the possibility of creating similar Pt-O-Pt sites on an alumina support, in which the parent catalyst is the single-atom Pt_1_/La-Al_2_O_3_ with a platinum loading of ~0.5 wt.% that we recently reported^11^. Through a similar redox activation protocol that we have applied to the Pt_1_/CeO_2_ catalysts, we created an activated Pt/La-Al_2_O_3_ catalyst having a portion of its platinum as the active Pt-O-Pt catalytic sites, according to CO-DRIFTS studies (Supplementary Fig. 38) and kinetic measurements of *E*_app_ (41 ± 2 kJ/mol, Supplementary Fig. 39) and reaction orders (0.3 and 0.1 for CO and O_2_, respectively, Supplementary Fig. 37). This evidence further supports our hypothesis about the indirect role of the ceria particles (10–30 nm) in influencing the intrinsic low-temperature CO oxidation catalysis of Pt-O-Pt/CeO_2_ under oxygen-rich conditions.

## Discussion

**Fig. 6:**
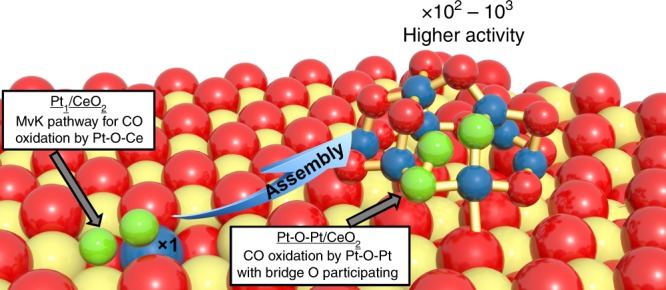
Different transition states for oxygen activation by the Pt_1_-O-Ce and the Pt-O-Pt ensembles. The detailed configurations for the CO oxidation cycles of the two groups of catalysts are illustrated in Fig. 3. The Pt_1_ single atoms are assembled into the more active Pt-O-Pt ensembles during the activation protocol. Color legend of atoms: Ce = yellow; Pt = blue; O = red; O atoms in the transition states for O_2_ activation are green. MvK = Mars–van Krevelen

It is worth noting that the concept of “maximized atom efficiency” is different from “maximized activity per atom.” Our results highlight that Pt_1_ indeed has maximized its material utilization efficiency, but there is large room to improve the activity per Pt atom. The solution from this work is to tackle the issue of lacking neighboring Pt atoms in the typical Pt_1_/CeO_2_ system. By forming the Pt-O-Pt catalytic unit in representative one-layer Pt_8_O_14_ cluster, the Pt atoms can now effectively activate and utilize the oxygen intermediates to catalyze the low-temperature CO oxidation. A recent work adjusted the Pt-O coordination number between 2 and 3 in PtO_*x*_ clusters by either reductive or oxidative treatment at 350 °C to modify the catalytic activity of PtO_*x*_/CeO_2_ nanowire catalysts within one order of magnitude for the CO oxidation under oxygen-rich conditions^24^. Despite that the platinum was not fully exposed in this prior work, as the dispersion ranged from 10 to 83% (Supplementary Table 1), the authors may have created a portion of similar sites as we did in this work (best-performing catalysts from the current work are on average six times more active by incorporating the possible impact of different reactant concentrations). Here, by maintaining 100% platinum dispersion, which means that all the Pt atoms are accountable for surface catalysis and there is a minimal amount of spectator Pt species to distort the averaged characterization results, we found that the coordination number of Pt-O may not be the most decisive factor for the much more dramatic change of the CO oxidation activity, because both our Pt_1_ and Pt-O-Pt structures have four oxygen atoms directly bonded to the platinum center at the starting point of each catalytic cycle. More importantly, the synergistic effect of the two, paired, platinum atoms in the Pt-O-Pt ensemble provides an alternative oxygen supply route independent of ceria substrates. This intrinsic catalytic difference between the isolated Pt_1_ and the paired Pt-O-Pt structure could likely not be overcome by merely changing the Pt-O coordination numbers and considering each Pt atom as an independent unit. The mechanistic importance of the Pt-O-Pt interaction is highlighted in this work, where most of the attention was on the Pt-O-Ce interaction in previous studies. As shown in Fig. 6, the rate-determining steps of the CO oxidation reaction by Pt_1_/CeO_2_ and Pt-O-Pt/CeO_2_ catalysts involve different sites and mechanisms for oxygen activation. The CO oxidation reaction proceeds through the MvK mechanism at the Pt_1_-O-Ce interface in the Pt_1_/CeO_2_ catalyst, while the reaction is more efficiently catalyzed by the Pt-O-Pt/CeO_2_ catalyst at its Pt-O-Pt unit with the bridge -O- participating. The similar feature might be shared with other oxide clusters having high metal dispersion and abundant undercoordinated metal sites.

The findings from this work should only be cautiously extended to a general type of clusters that retain a layered three-dimensional structure, where the lower dispersion and more saturated coordination environment of platinum bring more uncertainties. However, we infer that the Pt-O-Pt catalytic unit defined in this work is a prototype that illustrates an important advantage of catalysts with neighboring metal centers for efficient oxygen activation. Several recent reports are now indicating a general sense of agreement about the concept of using PGMs to create PGM-O-PGM catalytic units. Recent work by Zhao et al.^38^ report a synthesis of a Ir-O-Ir structure on α-Fe_2_O_3_, and the synergistic effects between the two nearby iridium atoms were inferred to explain the 2.6 times higher activity of the dinuclear structure from its single-Ir atom counterpart for solar water oxidation. Jeong et al.^39^ reported that the Rh_1_/CeO_2_ catalyst has low activity for C_3_H_6_ and C_3_H_8_ oxidation, but another speculated Rh ensemble catalyst is highly active for C_3_H_6_ and C_3_H_8_ oxidation. The elusive Rh ensembles cannot be observed in HAADF-STEM images, and the *k*^3^-weighted EXAFS radial distribution indicates two sets of Rh-O-Rh bonds with coordination numbers near 0.6 (means not even a dinuclear structure—instrumental signal limitations). More recently, Dessal et al.^40^ confirmed an enhancement of CO oxidation activity of the Pt/γ-Al_2_O_3_ catalysts when the Pt_1_ atoms were agglomerated into platinum oxide clusters with a Gaussian distribution of cluster sizes from 0.5 to 1.5 nm. These studies are using different PGM species, supports, PGM domain sizes, and different reactions, but the formation of the paired PGM-O-PGM bond is the common variable behind the improved catalytic performance. We believe this is where the general implication of this material development and mechanistic investigation work resides.

One should also avoid oversimplifying this work as any dinuclear Pt-O-Pt structures can be more active than the single-atom Pt species in activating [O] intermediates. Notably, one of the platinum atoms in this highly active Pt-O-Pt catalytic unit does not have any direct -O- linkages to the ceria support, and it is in fact the enabler for facile O migration and fast CO oxidation. Indeed, the lattice oxygen of ceria is critical for the formation of stable Pt-O bonds according to our XAS analyses. However, the ceria is also shown to be largely a spectator species in the low-temperature CO oxidation reaction catalyzed by the Pt-O-Pt/CeO_2_ systems. We envision that, if the Pt-O-Pt unit can be stabilized through similar oxygen linkages, a similar catalytic species may be created on various support substrates other than ceria. The formation of a Pt-O-Pt structure on activated Pt/La-Al_2_O_3_ supports this hypothesis (Supplementary Figs. 37–39), but developing alternative preparation methods to build the exclusive catalytic sites on the alumina support needs further effort that is outside the scope of this work.

The exception from the general assessment of the present work is most likely to happen when quantum-size effects become evident in small ceria nanoclusters (especially <5 nm). Elegant studies showed that the oxygen vacancy formation in CeO_2_ nanoclusters exposing small O-terminated (111) and (100) facets is more probable compared to the extended CeO_2_ surfaces^41–44^. Theoretical evidence shows that the oxygen reverse spillover from these small ceria nanoparticles to the supported Pt species can generally be a more favorable process compared with Pt species supported on larger ceria particles, the latter of which is typically represented by slab computational models^9,45,46^. Such an unusually high oxygen mobility from the ceria have been predicted by modeling studies on CeO_2_ nanoclusters comprising from about 60 to over 200 atoms, where the largest Ce_140_O_180_ has an approximate size of ~2.4 nm^42,47,48^. The supporting experimental evidence was provided by carefully growing small Pt/CeO_2_ nanoparticles on a CeO_2_(111) film under ultrahigh vacuum—these grown ceria nanoclusters are only about ~3 nm in diameter and 0.4 nm in height^29,43^. We emphasize that the these CeO_2_ nanoclusters (up to ~3–4 nm) with extraordinary capability for generating mobile oxygen species are intrinsically different from the 10–30 nm ceria particles (homemade and commercial) used in this work. For example, small ceria nanoparticles of 3–4 nm were shown to improve the catalytic activity of gold species for CO oxidation by about two magnitudes^49,50^, but this highly active oxygen supply disappears quickly for regular ceria nanoparticles that are larger than 10 nm in automotive applications^51–53^. Typical calcination treatment from 300 to 800 °C to fully decompose cerium precursors and to form stable ceria structures usually leads to the CeO_2_ particle size from 10 to 30 nm^51,52^. These ceria particles have abundant stable CeO_2_(111) facets^54,55^. These differences lead to the observation that our single-atom Pt_1_(IV)-O_4_ is mostly observed on the stable CeO_2_(111) surfaces of a 10–30 nm ceria particle, although a sinter-resistant single-atom structure Pt_1_(II)-O_4_ sits at the less stable CeO_2_(100) nanofacets of the small ceria nanoclusters (e.g., 1–3 nm)^29,56^. Of course, the small fraction of rounded edges, steps and kinks in our 10–30 nm ceria particles may well have led to the presence of a tiny portion of active sites as Pt-O-Pt plus active ceria substrates. These nonuniformities could be part of the reason for the deviations of kinetics between the computational results and experimental measurements. A success of synthesizing and stabilizing tiny ceria nanoclusters and anchoring an appreciable amount of the targeted platinum structure (e.g., the Pt-O-Pt) onto them bodes the ultimate solution of best using both the platinum and ceria substrate.

However, the bottom line is, when widely available ceria particles with larger size (>10 nm) and mediocre oxygen mobility are being adopted as an industrial catalyst support, activating the single-atom Pt to form the paired Pt-O-Pt ensemble is an effective way to create an alternative oxidative reaction pathway to benefit the low-temperature reactions. These tunable catalytic systems, either at a single-atom form or a Pt-O-Pt structure, may serve as powerful platforms for future studies of many other reactions.

## Methods

### Catalyst preparation

Platinum was loaded onto the ceria supports by a strong electrostatic adsorption method^57^. H_2_PtCl_6_ was chosen as the platinum precursor, because the platinum–ligands complex anions can evenly adsorb on the positively charged –O-Ce-OH_2_^+^ surface sites on ceria as single-atom layers when the pH of the solution is below the point of zero charge of ceria. To begin, the pH value of the H_2_PtCl_6_ solution was adjusted to pH ≈ 9 by ammonia. The transparent solution was stirred at 70 °C overnight to allow the substitution of -Cl in [PtCl_6_]^2−^ by -OH in solution. The as-prepared ceria powder was then added into the solution to adsorb the preformed [Pt(OH)_6_]^2−^ as a single-atom Pt layer. The concentrations of the platinum precursor and the amount of ceria were varied to keep the same solid–liquid contact interface of 500 m^2^/L. We washed the filtration cake (filtered catalyst) with a total of 2 L distilled water at 80 °C during each sample filtration. The obtained samples were dried at 100 °C overnight, then calcined in air at 500 °C for 3 h, followed by H_2_ reduction at 250 °C for 0.5 h to further remove any possible residual -Cl, and finally calcined in air at 500 °C for 1 h. These Pt_1_/CeO_2_ samples are hereafter referred to as the “as-prepared” catalysts, and designated as “Pt_1_/CeO_2_-a, Pt_1_/CeO_2_-b, and Pt_1_/CeO_2_-c”. None of the Pt-containing components were detected as crystallized structures (Supplementary Figs. 6–8, 11–16), and limited changes happened to the BET surface areas, that is, they changed to 74, 60, and 44 m^2^/g from 80, 64, and 51 m^2^/g, respectively. Our Pt_1_/CeO_2_ samples were calcined at 500 °C, so the bulk diffusion of the Pt into CeO_2_ that usually occurs above 700 °C is limited^58,59^. The nearly 100% Pt dispersion (measured at room temperature by a CO chemisorption method that passivates the ceria support in directly contributing to CO adsorption^39,60,61^) and the measured catalytic activity in line with published data (Supplementary Table 1) confirm that the Pt_1_ species are accessible to the reactants.

After mild reduction at 200 °C in 5% H_2_, and subsequent exposure of these Pt_1_/CeO_2_ catalysts to a CO plus O_2_ feed stream ([CO] = 1000 ppm, [O_2_] = 5%, balanced with N_2_) at ambient temperature, we saw a notable enhancement of catalytic activity due to the restructuring of the platinum species. The reason for using diluted H_2_ at mild temperatures is to adequately break the Pt-O bond that holds the single-atom Pt in place while avoiding extensive metal Pt sintering^30,31,62^. Our optimizations for reduction temperature and time are shown in Supplementary Fig. 1. Here, mild H_2_ reduction at 200 °C breaks the Pt-O-Ce bond in the Pt_1_-O_*x*_ single-atom structure, because all the reducible oxygen species near Pt–ceria interface are consumed below 200 °C according to H_2_-TPR for the Pt_1_/CeO_2_ catalysts (Fig. 5a). During the optimized H_2_ reduction phase, we expect the single-atom Pt_1_/CeO_2_ catalysts to generate abundant undercoordinated Pt atoms as nanorafts under the rather mild reduction condition and relatively short reduction time^30,31,62^. During the phase of reoxidation using a mixture of CO plus O_2_, both CO and O_2_ can induce a Pt restructuring depending on their respective pressure^63–66^. In general, O_2_ molecules tend to coordinate with Pt to form nano islands of multilayered α-PtO_2_-like oxides^67^. This is an unwanted outcome for the scope of this work, because the formation of the multi-layer spherical platinum oxide structures may result in the creation of Pt atoms with nonuniform chemical environment depending on their relative location in the platinum particle and from the ceria support. Characterizing such a mixed batch of catalytic species will generate average quantities and even distorted results, which will mask the characteristics of the active species^4,6^. Some Pt atoms may also be buried in the particle bulk, losing their ability to catalyze surface reactions. To prevent the formation of bulk particles, CO molecules were added to attach to Pt surfaces as ligands to cause CO-CO repulsion between nearby Pt sites^65^. As shown in Supplementary Fig. 2, having a trace amount of CO in the diluted oxygen feed stream is indeed helpful to activate the catalysts. These treatment steps lead to the formation of the Pt-O-Pt structure on ceria according to our characterization studies. Therefore, these activated samples are denoted as “Pt-O-Pt/CeO_2_.” We excluded the impact of chloride on the change of catalytic activities, as all our Pt_1_/CeO_2_ and Pt-O-Pt/CeO_2_ catalysts show a minimal and similar chloride concentration of 70–90 ppm according to ion chromatography analysis. The Pt-related catalytic sites were characterized by STEM, CO chemisorption, XPS, XAS, and H_2_ TPR after exposure to reaction conditions as the working catalysts.

### CO oxidation tests and kinetics

The CO oxidation reaction was conducted in a packed-bed tubular reactor. A 25 mg powder sample was diluted with 200 mg quartz sand in the catalyst bed. The catalysts were tested in an O_2_-rich gas atmosphere to reflect the lean-burn gasoline and diesel engine conditions. The test procedures were as follows: first, we ramped up the reactor temperature to 500 °C in 20% O_2_ balanced with N_2_ at a heating rate of 10 °C/min, and held for 30 min. Next, we cooled down the reactor to near-ambient temperature with an N_2_ purge until the temperature of the catalyst bed was stable. After the CO oxidation reaction feed stream ([CO] = 1000 ppm, [O_2_] = 5%, balanced with N_2_ at a flow rate of 1000 mL/min) was switched in. The steady-state and light-off conversion rates were measured at elevated temperatures after the baseline readings became stable at near-ambient temperature. To activate the as-prepared sample, a treatment including a reduction at 200 °C in 5% H_2_ for 15 min and a subsequent exposure to the CO plus O_2_ atmosphere at ambient temperature was used before the reaction. Wet CO oxidation followed the same test procedure with a feed stream containing 3% H_2_O ([CO] = 1000 ppm, [O_2_] = 5%, [H_2_O] = 3%, balanced with N_2_ at a flow rate of 1000 mL/min). The concentrations of CO and CO_2_ were monitored by an MKS 2030 gas cell Fourier-transform infrared spectroscopy, and the O_2_ concentration was measured by mass spectroscopy (Hiden HPR20). Kinetic measurements were carried out on the same equipment setups. A typical flow rate ranging from 1200 to 1500 mL/min was used for 10 to 20 mg of catalyst in each test to ensure the catalytic reaction was free of heat and mass transfer effects. The CO conversion was therefore kept below 20% for all the reaction rate measurements.

### DFT modeling approach

Spin-polarized DFT calculations were performed using the Vienna Ab initio Simulation Package (VASP)^68,69^ with the projector augmented-wave method to treat electron–ion interactions^70^. The Perdew–Burke–Ernzerhof (PBE) exchange-correlation functional^71^ was used as the density functional approximation for all genetic algorithm (GA) and GCMC calculations. The revised Perdew–Burke–Ernzerhof (RPBE) exchange-correlation functional^72^ was used to calculate the CO oxidation catalytic cycle because of its superior ability to predict accurate adsorption energies over PBE as compared with the experiment^72^. The strongly correlated 4*f* electron of cerium was treated with the DFT + U correction, using a *U*_eff_ = 5 eV for both PBE and RPBE calculations^73^. Brillouin zone sampling was restricted to the *Γ* point for all DFT calculations. To avoid artificial self-interactions between slabs due to periodic boundary conditions, the surface slabs were separated by a vacuum layer of 15 Å. A GA was used to find the global minimum structure of a Pt_8_ cluster supported on ceria (Pt_8_/CeO_2_). The most stable Pt_8_/CeO_2_ structure is used as the initial configuration for the GCMC simulations. For the GA and GCMC simulations, a *p*(4 × 4) CeO_2_(111) surface with one O-Ce-O layer was used as the support model without geometry relaxation to improve the calculation efficiency. We did not include the presence of persistent oxygen vacancies on CeO_2_(111) surfaces during our CO oxidation mechanistic studies, as their rapid healing has been demonstrated by both theory and experiments^16–18^, especially under oxygen-rich reaction conditions^17^. The plane-wave cutoff energy was set to 300 eV and the convergence threshold for geometry optimizations was specified to 10^–3^ eV for both GA and GCMC calculations.

A *p*(4 × 4) CeO_2_(111) surface with two O-Ce-O layers was used as the support for studying the CO oxidation catalytic cycle on both the Pt_1_/CeO_2_(111) and Pt_8_O_14_/CeO_2_(111) systems. To analyze the impact of the exposed ceria facet on CO oxidation, the representative Pt_8_O_14_ structure searched by GCMC^18^ simulations on CeO_2_(111) was deposited on two O-Ce-O-layered (110) and (100) surfaces with each Pt atom binding four oxygen atoms according to our XPS and EXAFS data. Experimental observations^74,75^ suggest that the (100) surface is terminated by 0.5 monolayer of oxygen. To obtain a consistent model, we constructed the oxygen terminated (100) surface by removing half of the oxygen atoms on the top and bottom surfaces (Supplementary Fig. 36). The top O-Ce-O layer, adsorbates, Pt single atom, and Pt_8_O_14_ cluster could relax during geometry optimization and transition state searches. A plane-wave basis with a cutoff energy of 400 eV was chosen. The climbing-image nudged elastic band (CI-NEB) method^76,77^ was used to find the transition states involved in the CO oxidation mechanism. The CI-NEB force tolerance was set to 0.05 eV/Å. CO vibrational frequency was calculated within the harmonic approximation. The calculated vibrational frequency of gaseous CO by DFT-RPBE was 2105 cm^−1^, which is 65 cm^−1^ smaller than the true value measured in our experiment (2170 cm^−1^). Thus, we applied a 65 cm^−1^ rigid shift to all the calculated CO vibrational frequencies on Pt_1_/CeO_2_ and Pt_8_O_14_/CeO_2_ catalysts to compare with our experimental DRIFTS spectra. The zero-point energy correction was not considered. Details on GA and GCMC simulations as well as the mean-field microkinetic simulations approach are presented in the Supplementary Information.

## Supplementary information

Supplementary Information

## Data Availability

The data that support the findings of this study are available from the corresponding authors upon request.

## References

[1] Thomas JM (2015). Tens of thousands of atoms replaced by one. Nature.

[2] Yang X-F (2013). Single-atom catalysts: a new frontier in heterogeneous catalysis. Acc. Chem. Res..

[3] Liu L, Corma A (2018). Metal catalysts for heterogeneous catalysis: from single atoms to nanoclusters and nanoparticles. Chem. Rev..

[4] Yang M, Allard LF, Flytzani-Stephanopoulos M (2013). Atomically dispersed Au-(OH)*x* species bound on titania catalyze the low-temperature water-gas shift reaction. J. Am. Chem. Soc..

[5] Yang M (2014). Catalytically active Au-O(OH)*x*-species stabilized by alkali ions on zeolites and mesoporous oxides. Science.

[6] Yang M (2015). A common single-site Pt(II)-O(OH)*x*- species stabilized by sodium on “active” and “inert” supports catalyzes the water–gas shift reaction. J. Am. Chem. Soc..

[7] Office of the Federal Register. National archives and records administration. *Fed. Regist.* **81**, 73478–74274 (2016).

[8] Jones J (2016). Thermally stable single-atom platinum-on-ceria catalysts via atom trapping. Science.

[9] Nie L (2017). Activation of surface lattice oxygen in single-atom Pt/CeO_2_ for low-temperature CO oxidation. Science.

[10] Zhang Z (2017). Thermally stable single atom Pt/m-Al_2_O_3_ for selective hydrogenation and CO oxidation. Nat. Commun..

[11] Wang H (2019). Single-site Pt/La-Al_2_O_3_ stabilized by barium as an active and stable catalyst in purifying CO and C_3_H_6_ emissions. Appl. Catal. B.

[12] Kistler JD (2014). A single-site platinum CO oxidation catalyst in zeolite KLTL: microscopic and spectroscopic determination of the locations of the platinum atoms. Angew. Chem. Int. Ed..

[13] Holmgren A, Azarnoush F, Fridell E (1999). Influence of pre-treatment on the low-temperature activity of Pt/ceria. Appl. Catal. B.

[14] Pereira-Hernandez XI (2019). Tuning Pt-CeO_2_ interactions by high-temperature vapor-phase synthesis for improved reducibility of lattice oxygen. Nat. Commun..

[15] Liu H-H (2014). Oxygen vacancy promoted CO oxidation over Pt/CeO_2_ catalysts: a reaction at Pt–CeO_2_ interface. Appl. Surf. Sci..

[16] Trovarelli A (1996). Catalytic properties of ceria and CeO_2_-containing materials. Catal. Rev..

[17] Kopelent R (2015). Catalytically active and spectator Ce^3+^ in ceria-supported metal catalysts. Angew. Chem. Int. Ed..

[18] Liu JX, Su Y, Filot IAW, Hensen EJM (2018). A linear scaling relation for CO oxidation on CeO_2_-supported Pd. J. Am. Chem. Soc..

[19] Wang C (2016). Water-mediated Mars–Van Krevelen mechanism for CO oxidation on ceria-supported single-atom Pt1 Catalyst. ACS Catal..

[20] Moses-DeBusk M (2013). CO oxidation on supported single Pt atoms: experimental and ab initio density functional studies of CO interaction with Pt atom on theta-Al_2_O_3_(010) surface. J. Am. Chem. Soc..

[21] DeRita L (2017). Catalyst architecture for stable single atom dispersion enables site-specific spectroscopic and reactivity measurements of CO adsorbed to Pt atoms, oxidized Pt clusters, and metallic Pt clusters on TiO_2_. J. Am. Chem. Soc..

[22] Kale MJ, Christopher P (2016). Utilizing quantitative in situ FTIR spectroscopy to identify well-coordinated Pt atoms as the active site for CO oxidation on Al_2_O_3_-supported Pt catalysts. ACS Catal..

[23] Lee J (2018). Influence of the defect concentration of ceria on the Pt dispersion and the CO oxidation activity of Pt/CeO_2_. J. Phys. Chem. C.

[24] Ke J (2015). Strong local coordination structure effects on subnanometer PtO*x* clusters over CeO_2_ nanowires probed by low-temperature CO oxidation. ACS Catal..

[25] Mhadeshwar AB, Vlachos DG (2004). Microkinetic modeling for water-promoted CO oxidation, water–gas shift, and preferential oxidation of CO on Pt. J. Phys. Chem. B.

[26] Xu L (2009). Direct evidence for the interfacial oxidation of CO with hydroxyls catalyzed by Pt/Oxide nanocatalysts. J. Am. Chem. Soc..

[27] Hatanaka M (2010). Ideal Pt loading for a Pt/CeO_2_-based catalyst stabilized by a Pt–O–Ce bond. Appl. Catal. B.

[28] Dvorak F (2016). Creating single-atom Pt–ceria catalysts by surface step decoration. Nat. Commun..

[29] Bruix A (2014). Maximum noble-metal efficiency in catalytic materials: atomically dispersed surface platinum. Angew. Chem. Int. Ed..

[30] Gatla S (2016). Room-temperature CO oxidation catalyst: low-temperature metal–support interaction between platinum nanoparticles and nanosized ceria. ACS Catal..

[31] Ganzler AM (2017). Tuning the structure of platinum particles on ceria in situ for enhancing the catalytic performance of exhaust gas catalysts. Angew. Chem. Int. Ed..

[32] Aleksandrov HA, Neyman KM, Hadjiivanov KI, Vayssilov GN (2016). Can the state of platinum species be unambiguously determined by the stretching frequency of an adsorbed CO probe molecule?. Phys. Chem. Chem. Phys..

[33] Mars P, van Krevelen DW (1954). Oxidations carried out by means of vanadium oxide catalysts. Chem. Eng. Sci..

[34] Song W (2018). Combination of density functional theory and microkinetic study to the Mn-Doped CeO_2_ catalysts for CO oxidation: a case study to understand the doping metal content. J. Phys. Chem. C..

[35] Kim HY, Henkelman G (2013). CO oxidation at the interface of Au nanoclusters and the stepped-CeO2(111) surface by the Mars–van Krevelen mechanism. J. Phys. Chem. Lett..

[36] An K (2013). Enhanced CO oxidation rates at the interface of mesoporous oxides and Pt nanoparticles. J. Am. Chem. Soc..

[37] Cargnello M (2013). Control of metal nanocrystal size reveals metal-support interface role for ceria catalysts. Science.

[38] Zhao Y (2018). Stable iridium dinuclear heterogeneous catalysts supported on metal-oxide substrate for solar water oxidation. Proc. Natl. Acad. Sci. USA.

[39] Jeong H (2018). Fully dispersed Rh ensemble catalyst to enhance low-temperature activity. J. Am. Chem. Soc..

[40] Dessal C (2019). Dynamics of aingle Pt atoms on alumina during CO oxidation monitored by operando X-ray and infrared spectroscopies. ACS Catal..

[41] Migani A (2010). Greatly facilitated oxygen vacancy formation in ceria nanocrystallites. Chem. Commun. Chem..

[42] Vayssilov GN, Migani A, Neyman K (2011). Density functional modeling of the interactions of platinum clusters with CeO_2_ nanoparticles of different size. J. Phys. Chem. C.

[43] Vayssilov GN (2011). Support nanostructure boosts oxygen transfer to catalytically active platinum nanoparticles. Nat. Mater..

[44] Lykhach Y (2017). Redox-mediated conversion of atomically dispersed platinum to sub-nanometer particles. J. Mater. Chem. A.

[45] Mao M (2016). Metal support interaction in Pt nanoparticles partially confined in the mesopores of microsized mesoporous CeO_2_ for highly efficient purification of volatile organic compounds. ACS Catal..

[46] Spezzati G (2017). Atomically dispersed Pd−O species on CeO_2_(111) as highly active sites for low-temperature CO oxidation. ACS Catal..

[47] Loschen C (2008). Density functional studies of model cerium oxide nanoparticles. Phys. Chem. Chem. Phys..

[48] Sk MA (2014). Oxygen vacancies in self-assemblies of ceria nanoparticles. J. Mater. Chem. A.

[49] Carrettin S (2004). Nanocrystalline CeO_2_ increases the activity of Au for CO oxidation by two orders of magnitude. Angew. Chem. Int. Ed..

[50] Huang X-S (2009). Morphology effects of nanoscale ceria on the activity of Au/CeO_2_ catalysts for low-temperature CO oxidation. Appl. Catal. B.

[51] Bunluesin T, Gorte RJ, Graham GW (1997). CO oxidation for the characterization of reducibility in oxygen storage components of three-way automotive catalysts. Appl. Catal. B.

[52] Cordatos H (1996). Effect of ceria structure on oxygen migration for Rh/ceria catalysts. J. Phys. Chem..

[53] Jeong H, Bae J, Han JW, Lee H (2017). Promoting effects of hydrothermal treatment on the activity and durability of Pd/CeO_2_ catalysts for CO oxidation. ACS Catal..

[54] Wang ZL, Feng X (2003). Polyhedral shapes of CeO_2_ nanoparticles. J. Phys. Chem. B.

[55] Zang C, Zhang X, Hu S, Chen F (2017). The role of exposed facets in the Fenton-like reactivity of CeO_2_ nanocrystal to the Orange II. Appl. Catal. B.

[56] Migani A (2010). Dramatic reduction of the oxygen vacancy formation energy in ceria particles: a possible key to their remarkable reactivity at the nanoscale. J. Mater. Chem..

[57] Regalbuto, J. in *Catalyst Preparation: Science and Engineering* (ed. Regalbuto, J.) (CRC Press, 2016).

[58] Fan J (2008). Thermal ageing of Pt on low-surface-area CeO_2_–ZrO_2_–La_2_O_3_ mixed oxides: effect on the OSC performance. Appl. Catal. B.

[59] Fan J, Wu X, Yang L, Weng D (2007). The SMSI between supported platinum and CeO_2_–ZrO_2_–La2O_3_ mixed oxides in oxidative atmosphere. Catal. Today.

[60] Takeguchi T (2005). Determination of dispersion of precious metals on CeO_2_-containing supports. Appl. Catal. A.

[61] Yoshida H (2018). Redox dynamics of Pd supported on CeO_2_–ZrO_2_ during oxygen storage/release cycles analyzed by time-resolved in situ reflectance spectroscopy. J. Phys. Chem. C.

[62] Lee J (2016). How Pt interacts with CeO_2_ under the reducing and oxidizing environments at elevated temperature: the origin of improved thermal stability of Pt/CeO_2_ compared to CeO_2_. J. Phys. Chem. C.

[63] Hicks RF, Qi H, Kooh AB, Fischel LB (1990). Carbon monoxide restructuring of palladium crystallite surfaces. J. Catal..

[64] Tao F (2009). Restructuring of hex-Pt (100) under CO gas environments: formation of 2-D nanoclusters. Nano Lett..

[65] Tao F (2010). Break-up of stepped platinum catalyst surfaces by high CO coverage. Science.

[66] Zhu Z (2012). Formation of nanometer-sized surface platinum oxide clusters on a stepped Pt(557) single crystal surface induced by oxygen: a high-pressure STM and ambient-pressure XPS study. Nano Lett..

[67] Butcher DR (2011). In situ oxidation study of Pt(110) and its interaction with CO. J. Am. Chem. Soc..

[68] Kresse G, Hafner J (1993). Ab initiomolecular dynamics for liquid metals. Phys. Rev. B.

[69] Kresse G, Furthmüller J (1996). Efficient iterative schemes for ab initio total-energy calculations using a plane-wave basis set. Phys. Rev. B.

[70] Blöchl PE (1994). Projector augmented-wave method. Phys. Rev. B.

[71] Perdew JP, Burke K, Ernzerhof M (1996). Generalized gradient approximation made simple. Phys. Rev. Lett..

[72] Hammer B, Hansen LB, Nørskov JK (1999). Improved adsorption energetics within density-functional theory using revised Perdew–Burke–Ernzerhof functionals. Phys. Rev. B.

[73] Loschen C, Carrasco J, Neyman KM, Illas F (2007). First-principles LDA+U and GGA+U study of cerium oxides: dependence on the effective U parameter. Phys. Rev. B.

[74] Wu Z (2014). Thiolate ligands as a double-edged sword for CO oxidation on CeO_2_ supported Au25(SCH_2_CH_2_Ph)18 nanoclusters. J. Am. Chem. Soc..

[75] Herman GS (1999). Surface structure determination of CeO_2_ (001) by angle-resolved mass spectroscopy of recoiled ions. Phys. Rev. B.

[76] Henkelman G, Uberuaga BP, Jónsson H (2000). A climbing image nudged elastic band method for finding saddle points and minimum energy paths. J. Chem. Phys..

[77] Henkelman G, Jónsson H (2000). Improved tangent estimate in the nudged elastic band method for finding minimum energy paths and saddle points. J. Chem. Phys..

