# Supplementary material for: Surpassing the single-atom catalytic activity limit through paired Pt-O-Pt ensemble built from isolated Pt1 atoms
Source: Nat Commun. 2019 Aug 23;10:3808. doi: 10.1038/s41467-019-11856-9 (PMC6707320; doi:10.1038/s41467-019-11856-9)
Supplement: Supplementary file 1 — Supplementary Information [file 41467_2019_11856_MOESM1_ESM.pdf]

Supplementary Information

Surpassing the single-atom catalytic activity limit through paired Pt-O-Pt ensemble built from isolated Pt_1 atoms

Hui Wang[1,†], Jin-Xun Liu[2,3†], Lawrence F. Allard[4], Sungsik Lee[5], Jilei Liu[6], Hang Li[1], Jianqiang Wang[1], Jun Wang[1], Se H. Oh[7], Wei Li[7], Maria Flytzani-Stephanopoulos[6], Meiqing Shen[1,8,9*], Bryan R. Goldsmith[2,3*], Ming Yang[7*]

[1]School of Chemical Engineering and Technology, Tianjin University, Tianjin, China.
[2]Department of Chemical Engineering, University of Michigan, Ann Arbor, MI, USA.
[3]Catalysis Science and Technology Institute, University of Michigan, Ann Arbor, MI, USA.
[4]Materials Science and Technology Division, Oak Ridge National Laboratory, Oak Ridge, TN, USA.
[5]X-ray Science Division, Argonne National Laboratory, Lemont, IL, USA.
[6]School of Chemical and Biological Engineering, Tufts University, Medford, MA.
[7]Chemical and Materials Systems Laboratory, General Motors Global Research and Development, Warren, MI, USA.
[8]State Key Laboratory of Engines, Tianjin University, Tianjin, China.
[9]Collaborative Innovation Center of Chemical Science and Engineering, Tianjin, China.

*Corresponding authors: Dr. M. Yang (ming.yang@gm.com),
Prof. M. Shen (mqshen@tju.edu.cn) and Prof. B. R. Goldsmith (bgoldsm@umich.edu)

†These authors contributed equally to this work.

Supplementary methods

Catalyst preparation

The ceria supports were prepared by a precipitation method. A $Ce(NO_3)_3$•$6H_2O$ (99 %, Rongruida, China) solution with a concentration of 1.5 M was added dropwise to an 8 M ammonia solution (analytical grade, KEWEI) at a volume ratio of 1:8, and an air flow was bubbled through the suspension during the entire synthesis. The suspension was maintained under continuous stirring at 90 °C for 6 hrs. Afterwards, polyethylene glycol 4000 and glycine were added, followed by spray drying of the suspension. The fresh ceria was obtained by calcining the dried powders sequentially at 300 °C for 2 hrs and 500 °C for 3 hrs. To create different ceria materials for systematic studies, the fresh ceria was hydrothermally treated in air with 10 % H_2O at (a) 600, (b) 700, or (c) 750 °C for 5 hrs to make the CeO_2-a, CeO_2-b and CeO_2-c as-prepared cerium oxides having different amounts of reducible oxygen species in their lattice. With increasing hydrothermal treatment temperature (600, 700, 750 °C), Brunauer-Emmett-Teller (BET) surface area decreased gradually as 80, 64, and 51 m^2/g, respectively.

Two types of commercial ceria provided by major catalyst suppliers, CeO_2-C (82 m^2/g) and CeO_2-H (167 m^2/g), were used to validate the general applicability of the findings in this work. Catalysts with 2.8 wt.% Pt loading were prepared by a widely used incipient wetness impregnation method with $Pt(NO_3)_2$ (Heraeus, 15.04 wt.%) as a precursor, because these conventional preparation steps better reflect the current industrial practice. The Pt-loaded samples were dried at 100 °C overnight, followed by air calcination at 500 °C for 3 hrs. The activation conditions were the same as our Pt-O-Pt/CeO_2-a, Pt-O-Pt/CeO_2-b, and Pt-O-Pt/CeO_2-c samples.

The Pt-related catalytic sites were characterized by STEM, CO chemisorption, XPS, XAS and H_2 TPR after exposure to reaction conditions as the working catalysts.

Catalyst characterizations

The BET surface area and pore structure were measured by N_2 physisorption at 77 K on an ASAP 2460 instrument (Micromeritics). The pore structure was analyzed by the Barrett-Joyner-Halenda method. All samples were degassed at 300 °C under vacuum for 5 hrs before the N_2 adsorption experiment. XRD patterns were collected on a Bruker D8 Focus equipped with nickel-filtered Cu K_α radiation (λ = 1.54056 Å), operating at 40 kV and 40 mA, and ranging from 20 to 90 ° with a 0.02 ° step size.

High-resolution transmission electron microscopy (HRTEM) work was done using a FEI Tecnai G2 F20 microscope, operating at 200 kV. Aberration-corrected high-angle annular dark-field scanning transmission electron microscopy (HAADF-STEM) images of Pt species were obtained at the Advanced Microscopy Laboratory at Oak Ridge National Laboratory, using a JEOL 2200FS instrument equipped with a hexapole corrector (CEOS GmbH, Heidelberg, Germany) on the illuminating lenses. The corrector permits imaging in HAADF mode at a nominal resolution of 0.07 nm, with a collection semi-angle of 26.5 mr and a beam current of ~30 pA. Images to reveal single platinum atoms were typically recorded between 8–10 M× direct magnification at 512×512 px with a 32 or 64 microsecond dwell time (giving 8.5 or 17 sec scan times). Because nanoparticles, small clusters, and single atoms are best imaged on thin and flat

support surfaces, the platinum species (> 150 total counts per sample) near the edge of the samples in different regions were counted and analyzed.

The X-ray absorption spectroscopy (XAS) scans were conducted in fluorescence mode with a 13-channel Ge detector at the 12-BM of the Advanced Photon Source at Argonne National Laboratory. The X-ray absorption edge energy for Pt L_3 $2p_{3/2}$ edge was calibrated to 11564 eV by Pt foil in each scan. For each data collection point, five consecutive scans were acquired. The reference spectra of Pt(IV), Pt(II), and Pt(0) (i.e., β-PtO_2 standard, $[Pt(NH_3)_4](NO_3)_2$, and Pt foil) were collected in an ambient environment. The Feff models of Pt foil, PtO_2 film, and PtO film were used to fit the 1st shell Pt–Pt and Pt–O coordination numbers. The XANES and EXAFS data were analyzed by the Athena and Artemis software, respectively. The XPS data were obtained on a Thermo ESCALAB 250XI. The dominant C1*s* peak was calibrated at 284.8 eV.

Diffuse Reflectance Infrared Fourier Transform Spectroscopy (DRIFTS) data were collected on a Nicolet 6700 FTIR equipped with an MCT detector at a resolution of 4 cm^{-1}. Each spectrum was the result of averaging 32 scans operated by the OMNIC software. The *in situ* DRIFTS experiment was conducted to simulate the CO oxidation condition in tube reactors. The sample cell was ramped from room temperature to 500 °C at a heating rate of 10 °C/min and was held at that temperature for 30 min. 20 % O_2 balanced with N_2 at a flow rate of 50 mL/min was used as the pretreatment gas during the time. Next, the sample cell was cooled down to 100 °C with an N_2 purge until the temperature was stable and then the background spectrum was collected. Afterwards, the proportional CO oxidation feed stream ([CO] = 0.6 %, $[O_2]$ = 30 %, balanced with N_2 at a flow rate of 50 mL/min) was flowed into the sample cell, and the evolution of the Infrared (IR) spectra were monitored. To activate the as-prepared sample, a treatment including a reduction at 200 °C in 5 % H_2 for 15 min and a subsequent exposure to the CO plus O_2 atmosphere ([CO] = 0.6 %, $[O_2]$ = 30 %) at ambient temperature was used before the CO adsorption measurement. The spectra were recorded at 110 °C, consistent with the temperature range we used in kinetic experiments.

The H_2 temperature programmed reduction (TPR) test was run on a Micromeritics Autochem II 2920 equipped with a TCD detector. The samples were first purged at 300 °C for 10 min with Ar to remove any remaining adsorbates. After being cooled back and stabilized at room temperature in the Ar flow, the samples were ramped to the target temperature in 10 % H_2 at a heating rate of 10 °C/min, during which the TCD recorded the H_2 consumption signal. The TCD signal was calibrated and quantified by a H_2 TPR experiment titrating the CuO standard with known mass and controlled purity. CO chemisorption measurements for the Pt catalysts were conducted on the same equipment settings. To measure the Pt dispersion, 100 mg of reaction-spent sample was purged at 300 °C for 10 min with Ar to remove any remaining adsorbates, then the sample was cooled down to room temperature in the same inert gas flow. Afterwards, 10 % CO_2 balanced with He was flowed into the sample tube to passivate the surface Ce(III) sites against possible contributions to CO adsorption during the CO chemisorption test[1-3]. After removing the excessive CO_2, 15 or more pulses of 5 % CO were injected into the carrier gas that flowed into the reactor, and the TCD readings were recorded. The volume of each CO pulse was 0.5796 mL. There was a minimum 1 min waiting period to allow purging the physisorbed CO from the catalyst between each pulse injection. A Pt:CO adsorption ratio of 1:1 was adopted to

calculate the integral amount of CO chemisorption and exposed Pt amount[4]. The measured Pt dispersions of the various reaction-spent Pt_1/CeO_2 and Pt-O-Pt/CeO_2 catalysts were between 91 to 97 %.

The kinetic measurements for the ceria materials (without platinum) were conducted on a house-built apparatus equipped with a Hiden HPR20 mass spectrometer to monitor the O_2, CO, and CO_2 concentrations. The CO oxidation feed stream with a total flow rate of 1000 mL/min was set as CO:O_2 = 2:1 (stoichiometric condition to highlight the lattice oxygen mobility from ceria). Each testing sample was first ramped to the highest reaction temperature, 515 °C, and treated in 2 % O_2 balanced with 1 % Ar/He for 30 min. The sample was then exposed to a CO oxidation reaction condition ([CO] = 2 %, [O_2] = 1 %, balanced with 1 % Ar/He, 10~15 mg sample was used to adjust the contact time so that the conversion remained < 20 %). The CO oxidation conversion from 515 to 465 °C was measured at intervals of 10 °C. A transient CO oxidation test using sequential CO and O_2 pulses, where the reducibility of the surface oxygen species on ceria is the key contributor to the redox performance. To examine this transient activity, the dynamic oxygen storage capacity (Dynamic OSC) test[5] was conducted on the same system from 300 to 600 °C at 25~50 °C intervals. A 25 mg sample blended with 200 mg quartz sand was packed in the tubular reactor. After the same pretreatment to clean up the samples, the switching pulses of 4 % CO and 2 % O_2 balanced with 1 % Ar/He at the same flow rate of 500 mL/min were sequentially directed into the reactor at the frequency of 0.1 Hz. The Dynamic OSC was calculated by integrating the formed CO_2, a product between CO gas molecule and –O from ceria surfaces, during the pulse experiment (Supplementary Figure 10a). This transient titration experiment put the emphasis on capturing the instantly available oxygen species from ceria in catalyzing the CO oxidation reaction.

Genetic algorithm calculations

A genetic algorithm (GA), which is a powerful evolutionary search method[6], was used to find the global minimum structure of a Pt_8 cluster supported on CeO_2(111). First, an initial population containing twelve different Pt_8 structures was randomly generated. Then, DFT calculations were performed to compute the total electronic energy of each Pt_8 structure in the population. During GA calculations, all the atoms in Pt_8 clusters on CeO_2(111) support were relaxed. Energies and bond distances were used to judge whether any Pt_8 structures were the same, and if so the non-unique structure was discarded to avoid multiple occurrences of one structure in the total population. The calculated total electronic energy was used to evaluate the fitness of each structure. The fitness function of ith candidate (F_i) is[6]:

$$F_i = \tfrac{1}{2}[1 - \tanh(2\rho_i - 1)] \text{ with } \rho_i = (E_i - E_{min})/(E_{max} - E_{min})$$

where E_i is the energy of the ith candidate, and E_{max} and E_{min} denote the maximum and minimum energy of any structure in the population.

Various operations were applied to existing structures in the population to generate new structures. A lower energy structure has a higher probability for doing the "crossover" and "mutation" operations[6], which are applied to generate a new population. For performing the crossover operation, two cutting planes are randomly selected and positioned in the common center of mass of two different structures. The new structure is generated by combining the cut

portions of the two different clusters together. For the mutation operation, the “rattle and twist” operations[6] are included. First, three Pt atoms in the Pt_8 cluster on ceria were moved a random distance in a random direction with perturbations between –0.3 Å and 0.5 Å. Then, the supported cluster can rotate a random angle ranging from 1° – 180° with respect to the surface normal. The above steps were repeated to optimize and generate a larger population of candidate Pt_8 structures on ceria. More than 300 structures were calculated, and no new structures are obtained after 30 cycles, suggesting the optimal Pt_8 cluster supported on CeO_2 was identified (Supplementary Figure 25a). A repeated simulation using a different initial population of structures identified the same optimal Pt_8/CeO_2 cluster.

Grand-canonical Monte Carlo (GCMC) simulations

GCMC-based DFT simulations were done to search the most stable structure of Pt_8O_x/CeO_2 under oxygen rich conditions at T = 350 K. The partial pressure of oxygen (p) is 0.05 bar, which is the same as in our experimental reaction studies. The chemical potential of Pt_8O_x/CeO_2 at a specified state can be expressed as:

$$\mu\ (T, p) = E(Pt_8O_x/CeO_2) + [\mu_{O2,reservoir} - x/2\ \mu_{O2}\ (T, p)]$$

where $\mu_{O2,reservoir}$ is the chemical potential of the infinite oxygen reservoir, x is the number of O atoms at a specific trial, and $E(Pt_8O_x/CeO_2)$ is the electronic energy of Pt_8O_x/CeO_2. The chemical potential of an O_2 molecule in the gas phase ($\mu_{O2}\ (T, p)$) is:

$$\mu_{O2}\ (T, p) = E_{O2} + \Delta\mu_{O2}\ (T, P_0) + k_BT\ \ln(p/P_0)$$

where E_{O2} is the electronic energy of O_2 and $\Delta\mu_{O2}\ (T, P_0) = \Delta H - T\Delta S$ can be obtained from a JANAF Thermochemical Table[7]. P_0 is the standard pressure of 1.01325 bar. Addition, deletion, and random movement of oxygen atoms were performed during each trial in the GCMC calculations. Also, a random selection of 25 % of the atoms in Pt_8O_x clusters can move freely when performing the movement operations. The chemical potential μ of the Pt_8O_x/CeO_2 system was calculated after each trial (addition, deletion, or movement). The metropolis algorithm was used to judge whether the present trial move should be accepted or rejected. At the equilibrium state, any addition, deletion or movement of oxygen will increase the total chemical potential of the system. After 500 trials, no new structures of Pt_8O_x/CeO_2 were generated at the given (T, p) conditions. Pt_8O_{14} was identified as the most stable (lowest chemical potential) structure. The $CeO_2(111)$ support is not changed during GCMC calculations (i.e., it has a fixed geometry).

First-principles microkinetic simulations

The CO oxidation rate and apparent activation barriers of the Pt_1/CeO_2 and Pt_8O_{14}/CeO_2 systems were predicted using first-principles microkinetic simulations to allow comparisons with our experimental kinetic measurements. The first-principles based approach for microkinetic simulations that we use has been presented in detail elsewhere[8], so here we only explain the main points. The DFT-calculated forward and backward activation energies were used to compute the rate constant of each elementary step. For surface reactions, the rate constant of step i is computed using the Arrhenius equation

$$k = Ae^{-\frac{Ea}{k_bT}} \qquad (1)$$

where k is the rate constant in s^{-1} and k_b, T, A, and E_a are the Boltzmann constant, temperature, pre-factor and the activation energy, respectively. The exponential pre-factor A is approximated as 10^{13} s^{-1} for all the elementary surface reactions.

The rate for non-activated molecular adsorption was calculated by the rate of surface impingement of gas-phase molecules. Based on the Hertz-Knudsen formula[9], the molecular adsorption rate constant is

$$k_{ads} = \frac{pA'}{\sqrt{2\pi m k_b T}} S \qquad (2)$$

Here, p is the partial pressure of the adsorbate in the gas phase and A' is the surface area of the adsorption site. m and S are the mass of the adsorbate and the sticking coefficient, respectively. The sticking coefficient used here was $S = 1$ for CO and O_2 adsorption.

For the desorption process, we assume there are three rotational degrees of freedom and two translational degrees of freedom in a transition state. Thus, the rate of desorption is given by

$$k_{des} = \frac{k_b T^3}{h^3} \frac{A'(2\pi k_b)}{\sigma \theta_{rot}} e^{-\frac{E_{des}}{k_b T}} \qquad (3)$$

where σ and θ are the symmetry number and the characteristic temperature for rotation. E_{des} is the desorption energy.

Differential equations for all the surface reaction intermediates were constructed using the rate constants and the set of elementary reaction steps. For a system of N elementary reaction steps, $2N$ rate expressions (i.e., both forward and backward reactions) can be obtained with the form

$$r_i = k_i \prod_{w=1}^{M} c_w^{\nu_w^i} \qquad (4)$$

here, k_i is the elementary reaction rate constant (see equation 1) and c_w and ν_w^i are the concentration and stoichiometric coefficient of component w in elementary reaction step i.

The CO oxidation rate was simulated by the MKMCXX program, which is a microkinetic modeling software for heterogeneous catalysis[8]. Steady-state coverages were calculated by integrating the ordinary differential equations in time until changes in the surface coverages were smaller than 10^{-12}. Because chemical systems typically give rise to stiff sets of ordinary differential equations, backward differentiation was used for time integration[8]. The rates of the individual elementary reaction steps were computed based on the steady-state surface coverages. In our simulations, the gas phase contains a mixture of CO and O_2 with partial pressures of 0.001 bar and 0.05 bar, respectively, which are the same as our experimental reaction conditions. The elementary reaction steps that contribute to the rate control of the overall reaction were determined by degree of rate control (DRC) analysis[10].

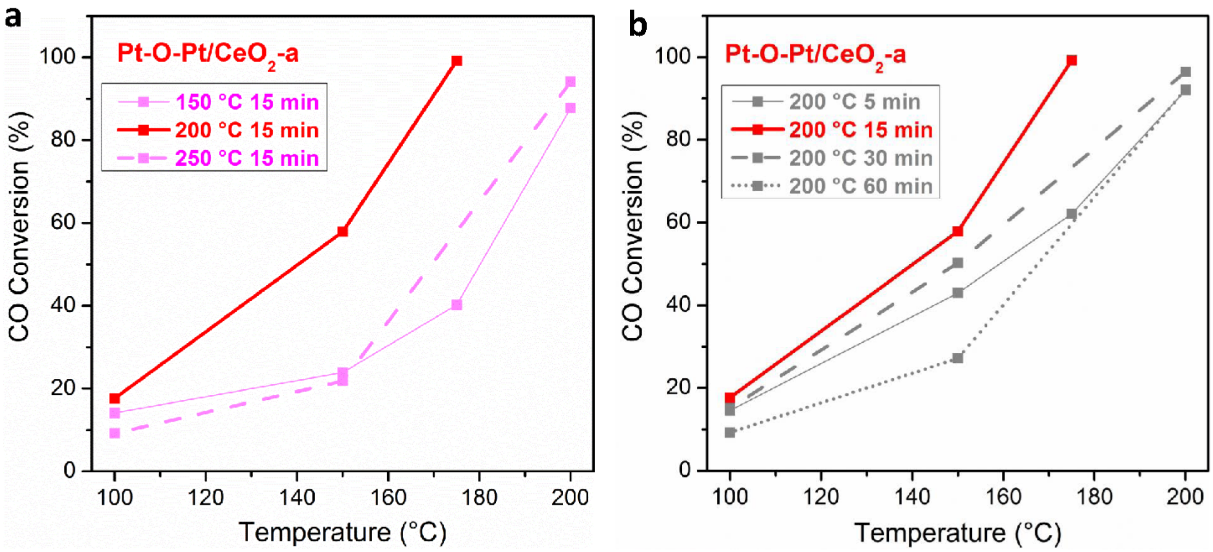

Supplementary Figure 1. Optimizing reduction conditions to make the Pt-O-Pt/CeO_2 catalysts. Investigating the optimal (**a**) reduction temperature and (**b**) reduction time. Red color indicates the optimal synthesis conditions. CO oxidation conditions: [CO] = 1000 ppm, [O_2] = 5 %, balanced with N_2 at a contact time of 2,400,000 ml g_{cat}^{-1} hour^{-1}.

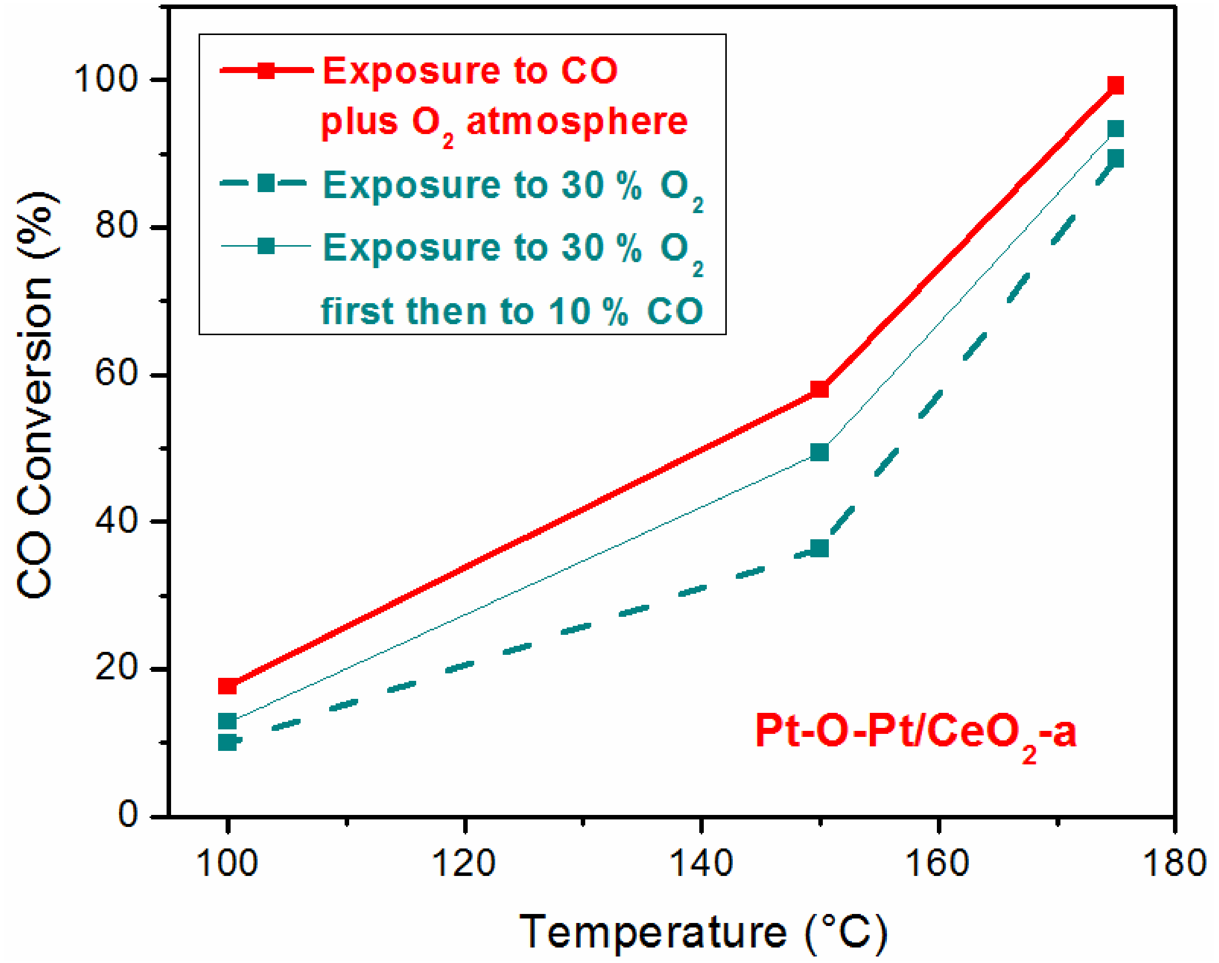

Supplementary Figure 2. Optimizing reoxidation conditions to make the Pt-O-Pt/CeO_2 catalysts. Impact of the reoxidation treatment with O_2, sequential O_2 and CO, and the mixture of CO plus O_2. Red color indicates the optimal oxidation condition. CO oxidation conditions: [CO] = 1000 ppm, [O_2] = 5 %, balanced with N_2 at a contact time of 2,400,000 ml g_{cat}^{-1} hour^{-1}.

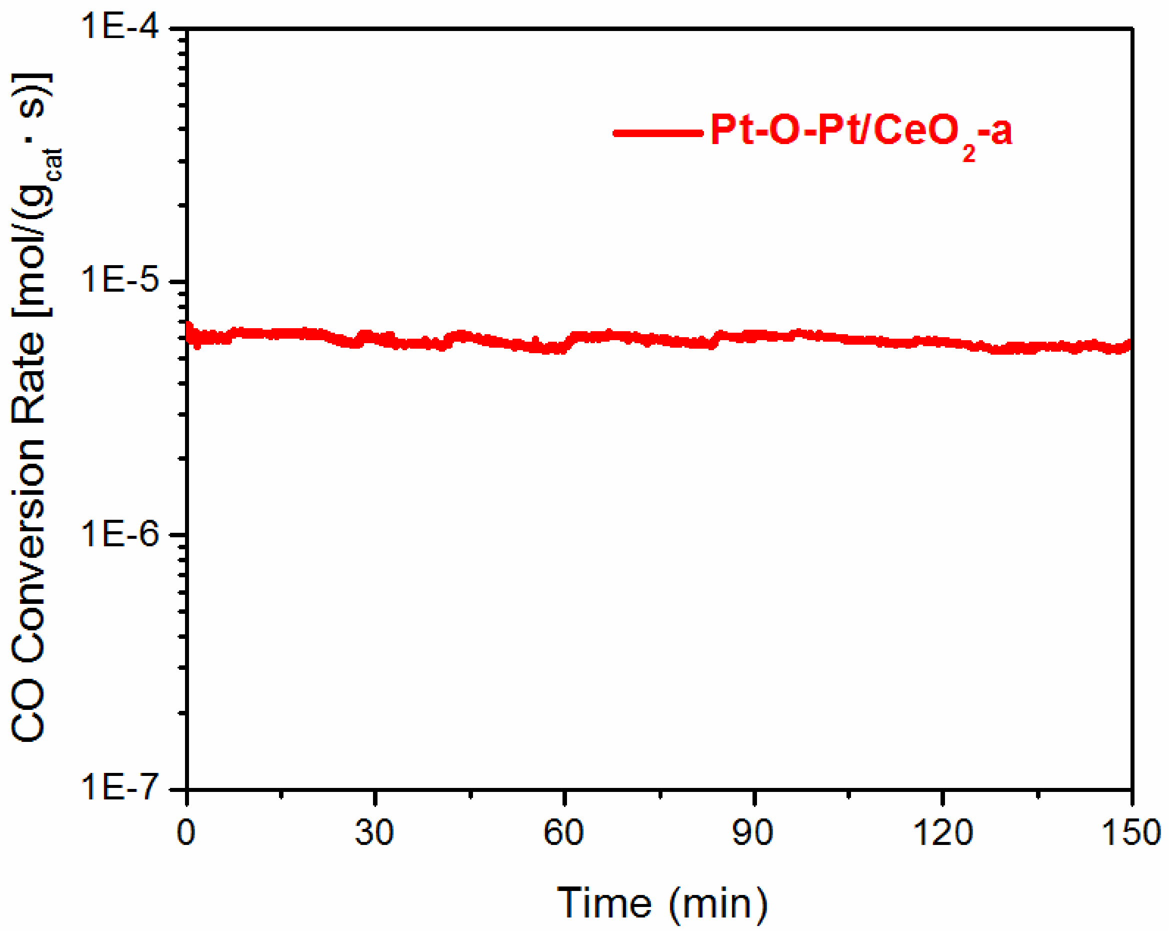

Supplementary Figure 3. Stable reaction rates for the Pt-O-Pt/CeO_2 catalysts. CO oxidation test was conducted at 100 °C with 5 % H_2 pulses at an interval of every 15 min (starts from time zero) to mimic fuel-rich spikes in real-world operations that may destroy the Pt-O-Pt structure.

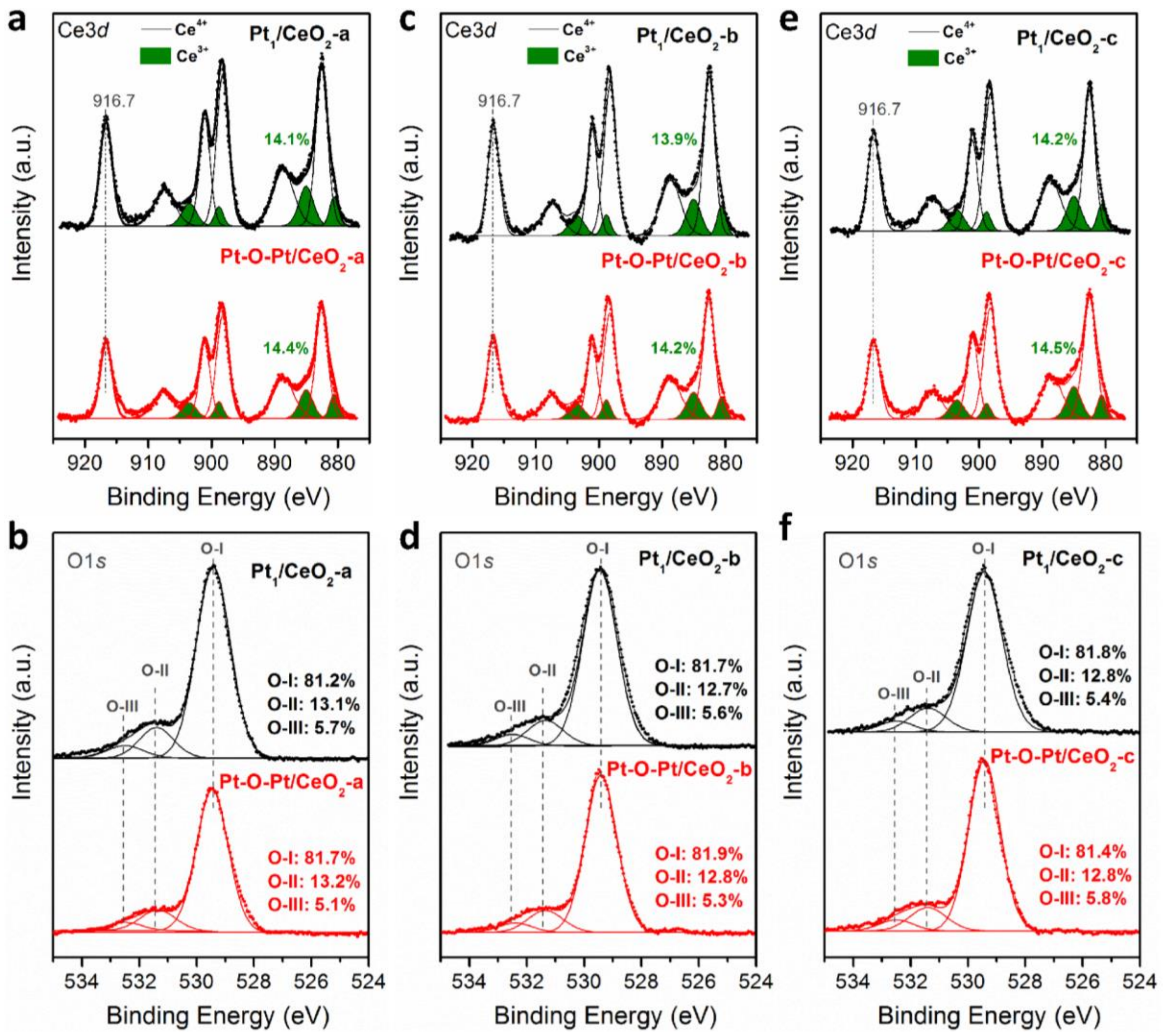

Supplementary Figure 4. Ce3*d* and O1*s* XPS spectra for the Pt_1/CeO_2 and Pt-O-Pt/CeO_2 catalysts. **a**, **c**, **e** show comparisons of Ce3*d* and **b**, **d**, **f** show the comparisons of O1*s* for the Pt_1/CeO_2 and Pt-O-Pt/CeO_2 catalysts. The labels and deconvolutions in Ce3*d* and O1*s* profiles are based on the following conventions[11-15]: O-I (529.3-529.5 eV) is attributed to oxygen in the CeO_2 lattice[16-18], O-II (531-532 eV) is attributed to surface oxygen associated with platinum, and possible –OH and carbonates[16,19-22], and O-III (532.4-532.8 eV) is attributed to adsorbed water molecules due to air exposure[22,23].

Supplementary Note 1:

We observe that the relative concentrations of both Ce^{3+} and various oxygen species are similar on the Pt_1/CeO_2 and Pt-O-Pt/CeO_2 catalysts. The slightly different availability of oxygen vacancies in the ceria surfaces is unlikely to be responsible for the large difference in catalytic activity between the two groups of catalysts. These detected Ce^{3+} species in the post-reaction catalysts are likely residing in the subsurface structure of the ceria particles, as the surface oxygen vacancies are thermodynamically preferred to fully heal under oxygen-rich reaction conditions[24,25], even in a few seconds under the reaction stream (1 % CO, 4 % O_2, 22–44 °C)[25].

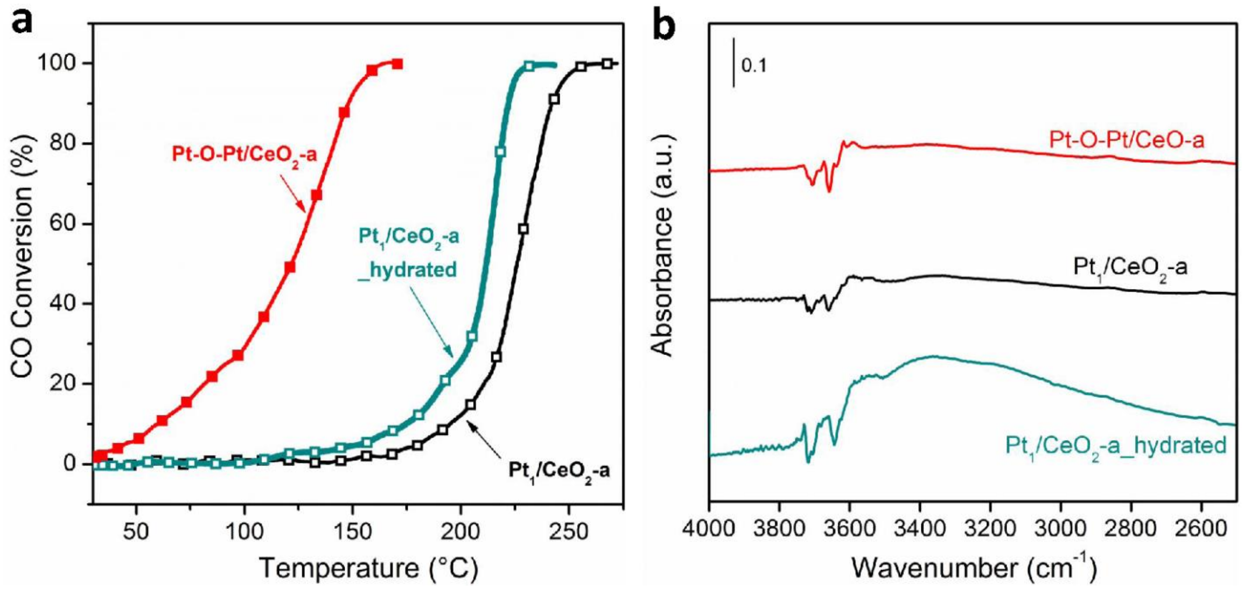

Supplementary Figure 5. Analyses of CO oxidation contribution from additional –OH species. a CO oxidation light-off performance of the Pt_1/CeO_2-a_hydrated, Pt_1/CeO_2-a, and Pt-O-Pt/CeO_2-a catalysts. **b** Surface hydroxyl (–OH) and surface adsorbed water comparison between the Pt_1/CeO_2-a, Pt_1/CeO_2-a_hydrated, and Pt-O-Pt/CeO_2-a catalysts.

Supplementary Note 2:

Recently, an alternative route to activate the Pt_1/CeO_2 catalyst has been reported by Nie, Mei, and Xiong et al.[26] who used a steam treatment method to activate the surface lattice oxygen of CeO_2 near Pt_1 sites. By flowing 10 % H_2O/Ar over a Pt_1/CeO_2 catalyst[27] at 750 °C for 9 h, the water molecules tend to fill the surface oxygen vacancies of ceria and form stable –OH groups anchored on the catalyst surface up to 767 ºC in theory. This decoration of –OH groups near the Pt_1 catalytic center results in a dramatic activity improvement (Supplementary Table 1). Although our activation conditions are very different from the above-mentioned work, the possible contribution of surface –OH decoration during the H_2O generation from the hydrogen reduction step of the activation protocol should be examined.

Following the work by Nie, Mei, and Xiong et al.,[26] we prepared a "Pt_1/CeO_2-a_hydrated" sample that reflects the maximal chance of our Pt_1/CeO_2-a catalyst to be decorated with the dissociated –OH groups near the Pt_1[28] during our activation process. To examine the case where abundant –OH species were generated from H_2O in our hydrogen reduction step of the activation protocol, and the single-atom feature of the platinum was retained, we flowed 4.5 % H_2O/N_2 instead of 5 % H_2 to the Pt_1/CeO_2 catalyst at 200 °C for 15 min (same temperature and time as for the original hydrogen reduction step), cooled down the sample to ambient temperature in 4.5 % H_2O/N_2, and held in a CO plus O_2 atmosphere for 5 min before ramping up the temperature for the reaction. As shown in Supplementary Figure 5a, the CO oxidation activity of the Pt_1/CeO_2-a_hydrated sample has improved from the original Pt_1/CeO_2-a sample but it is still much inferior to the Pt-O-Pt/CeO_2-a sample. Therefore, under our activation protocol conditions, the effort of decorating Pt_1/CeO_2 catalysts with –OH species cannot improve the catalytic performance to the

level of the Pt-O-Pt/CeO_2 samples. Here we only examined the effect of steam treatment under the condition that can be directly related to our low-temperature redox activation protocols. Compared with the work by Nie, Mei and Xiong et al.,[26] the Pt/CeO_2 catalysts used in this work under oxygen-rich reaction condition lack persistent surface oxygen vacancies, the steaming temperature at 200 ^{o}C is too low to allow the migration of any remaining oxygen vacancies from ceria bulk to surface[29], and the duration of the treatment time may not favor the kinetics of maximizing the –OH population. We believe these factors are unfavorable for developing an optimal hydroxyl-enriched Pt_1/CeO_2 catalyst as Nie, Mei, and Xiong et al. did[26], and the data provided is solely to show the possible –OH decoration on Pt_1 under our activation conditions cannot explain the observed high activity of the Pt-O-Pt catalytic site.

Next, we probe to what extent the extra –OH species were generated from our actual redox activation steps for making the Pt-O-Pt/CeO_2 catalysts. Supplementary Figure 5b compares the IR spectra of the parent Pt_1/CeO_2-a and activated Pt-O-Pt/CeO_2-a samples with the hydrated Pt_1/CeO_2-a_hydrated sample, focusing on the region where the –OH and H_2O featuring bands can be easily seen. The broad band region from 3800 to 2600 cm^{-1} can be assigned to –OH groups and adsorbed water molecules on the catalyst surface[30-32]. The band ranging from 3800 to 3400 cm^{-1} corresponds to –OH groups on ceria[33,34]. It becomes clear that, in contrast to that of the Pt_1/CeO_2-a_hydrated sample, the extent of surface change due to any creation of extra –OH and H_2O species on the Pt-O-Pt/CeO_2-a sample compared with the Pt_1/CeO_2-a is limited. Therefore, the role of –OH being the origin of the dramatically improved activity relative to our Pt-O-Pt/CeO_2 samples has been excluded by both CO oxidation activity tests and *in situ* DRIFTS. We reaffirm that the beneficial role of –OH species for Pt/CeO_2 catalysts in CO oxidation is true (Supplementary Figure 5), but the dramatic activity improvement from Pt_1 that we report in this work with and without water is driven by an important additional trigger—the Pt-O-Pt structure having oxygen-bridged Pt atoms.

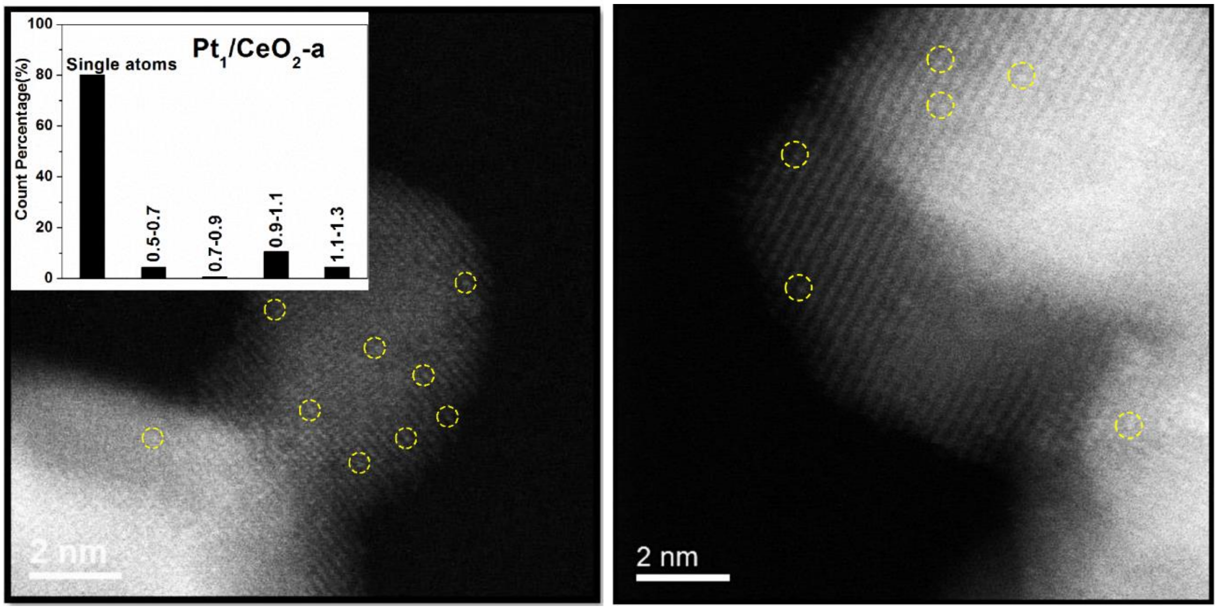

Supplementary Figure 6. HAADF-STEM images and the count frequency of Pt species in the Pt_1/CeO_2-a sample. The left and right images are two different representative views. Yellow circles are used to label the single Pt atoms. The size distribution (inset, left) in nm is based on over 150 observed platinum species counted from the high-magnification images (images recorded at 10 M× original magnification).

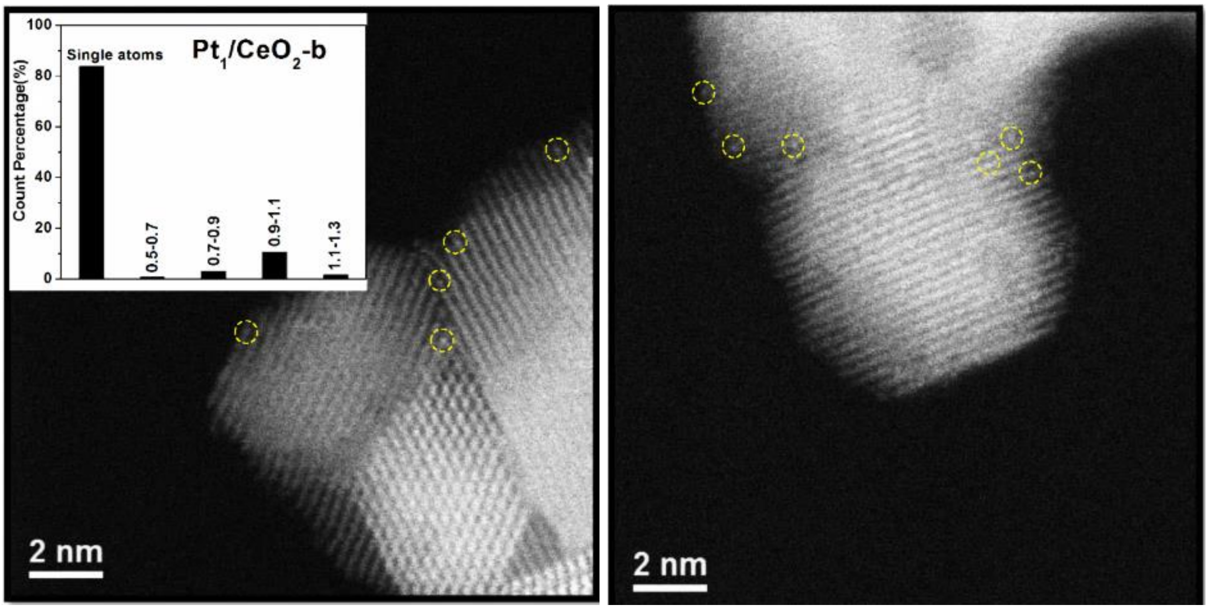

Supplementary Figure 7. HAADF-STEM images and the count frequency of Pt species in the Pt_1/CeO_2-b sample. The left and right images are two different representative views. Yellow circles are used to label the single Pt atoms. The size distribution (inset, left) in nm is based on over 150 observed platinum species counted from the high-magnification images (Both images recorded at 8 M× original magnification).

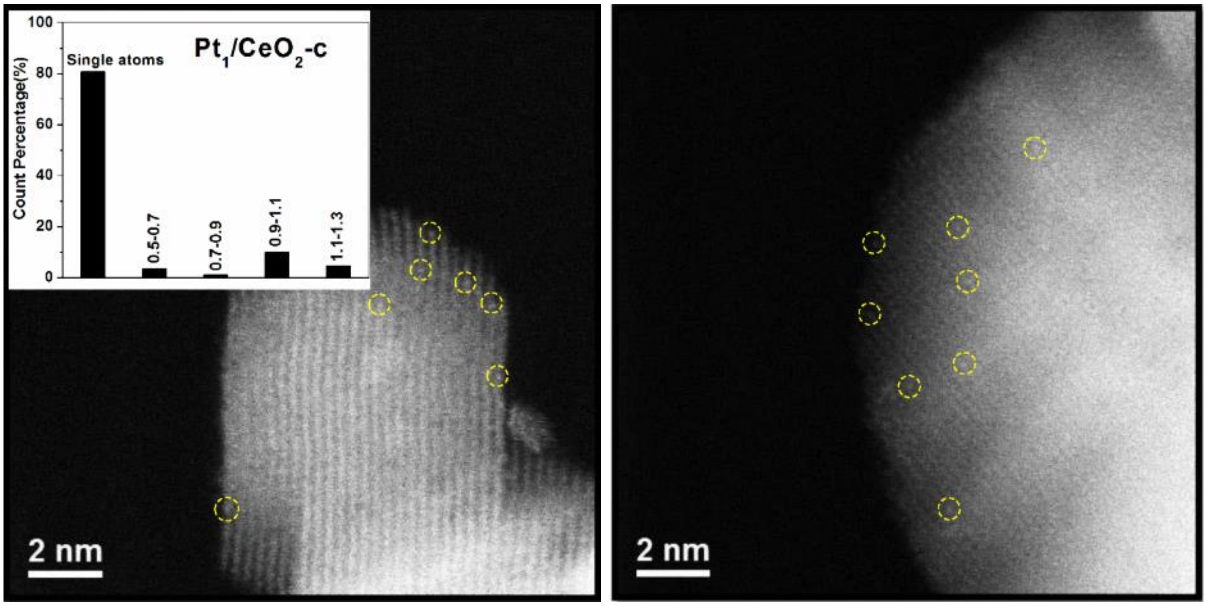

Supplementary Figure 8. HAADF-STEM images and the count frequency of Pt species in the Pt_1/CeO_2-c sample. The left and right images are two different representative views. Yellow circles are shown to label the single atoms. The size distribution (inset, left) in nm is based on over 150 observed platinum species counted from the high-magnification images (Both images recorded at 8 M× original magnification).

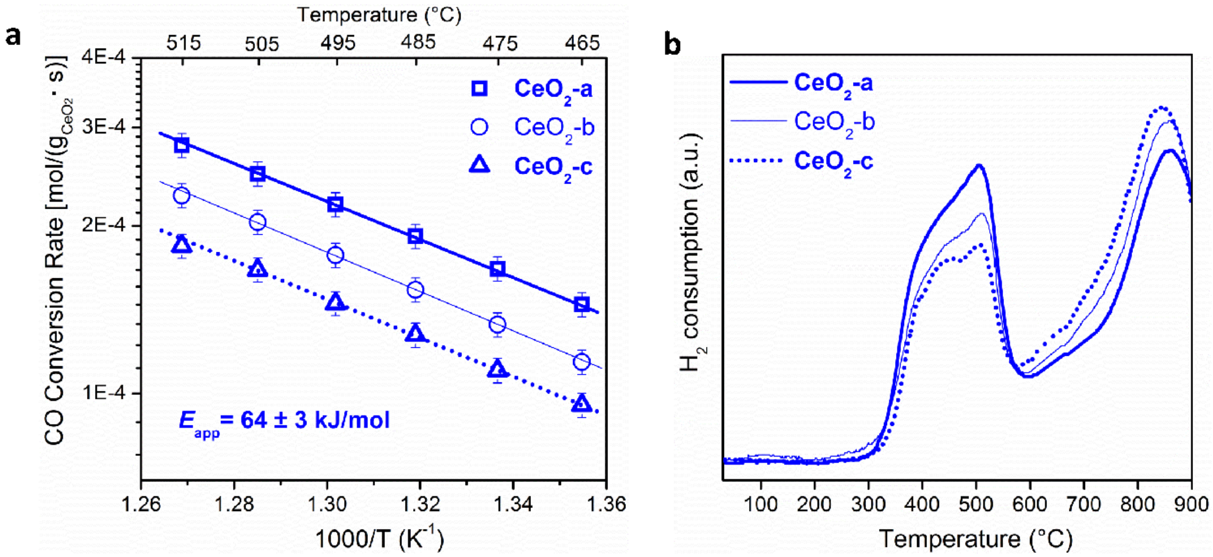

Supplementary Figure 9. Reaction tests and characterizations for the Pt-free ceria materials. a Arrhenius-type plot of CO oxidation rates and **b** H_2 TPR profiles for the Pt-free ceria materials. CO oxidation conditions: [CO] = 2 %, [O_2] = 1 %, balanced with N_2.

Supplementary Note 3:

The similar apparent activation energies of CeO_2-a, -b, and -c for catalyzing the CO oxidation reaction shows that the same intrinsic route of using the ceria lattice oxygen is followed regardless of their preparation protocol (Supplementary Figure 9a). These intrinsic consistencies allow us to focus on the different availabilities of the reducible oxygen species from these ceria samples. The different heat treatment temperatures for the CeO_2-a, -b, and -c during their preparation leads to changes in surface areas and ceria particle growth, which in turn impacts the availability of the reducible oxygen species in these ceria materials. The various abundancies of reducible oxygen species in these ceria samples manifests in the different reaction rates of using the Pt-free ceria materials to catalyze the CO oxidation under a steady-state condition (Supplementary Figure 9a). In Supplementary Figure 9b for the H_2 TPR profiles of the Pt-free ceria materials, the reduction peak < 600 °C is assigned to the depletion of the surface and near-surface reducible oxygen species, corresponding to the surface oxygen storage capacity, and the reduction at ~850 °C can be attributed to the further depletion of bulk oxygen[24,35]. We measure *ca.* 448, 345, and 287 μmol [O]/g_{CeO2} from 300 to 600 °C for CeO_2-a, -b, and -c, respectively. This difference proved to be true under both the steady-state reaction condition (Supplementary Figure 9a) and the dynamic CO-O_2 pulse reaction condition (Supplementary Figure 10). Higher activity for CO oxidation is expected when a ceria material has larger amount of reducible oxygen species in its near-surface structure.

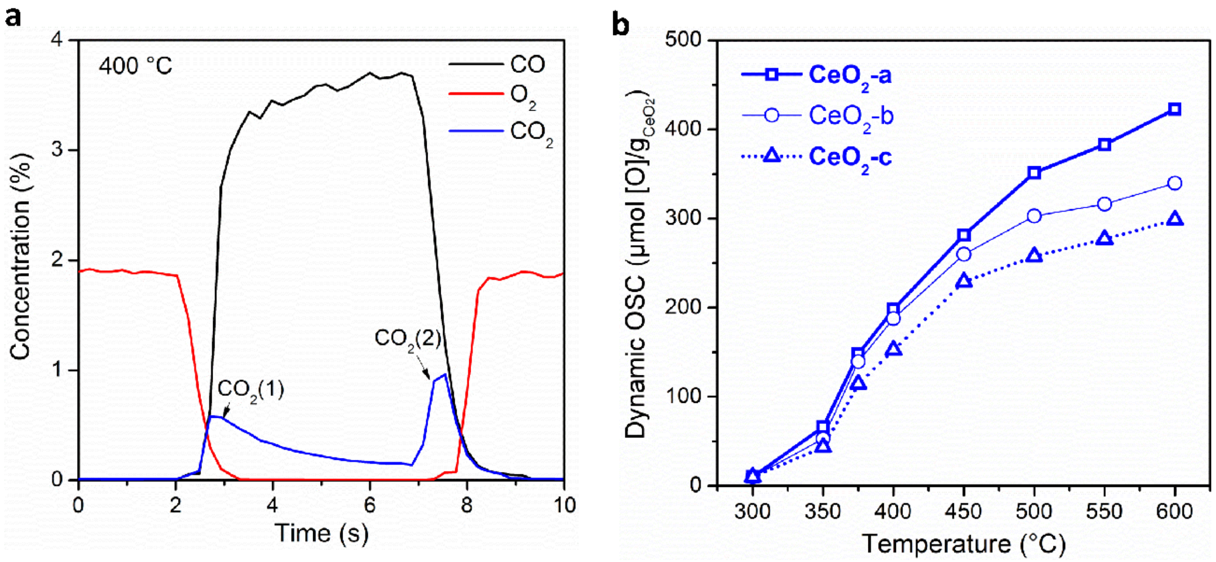

Supplementary Figure 10. Dynamic OSC of the Pt-free ceria supports. a Example showing one cycle of the transient CO-O_2 pulses and the CO_2 formation at 400 °C for CeO_2-a. **b** Dynamic OSC values vs. temperature.

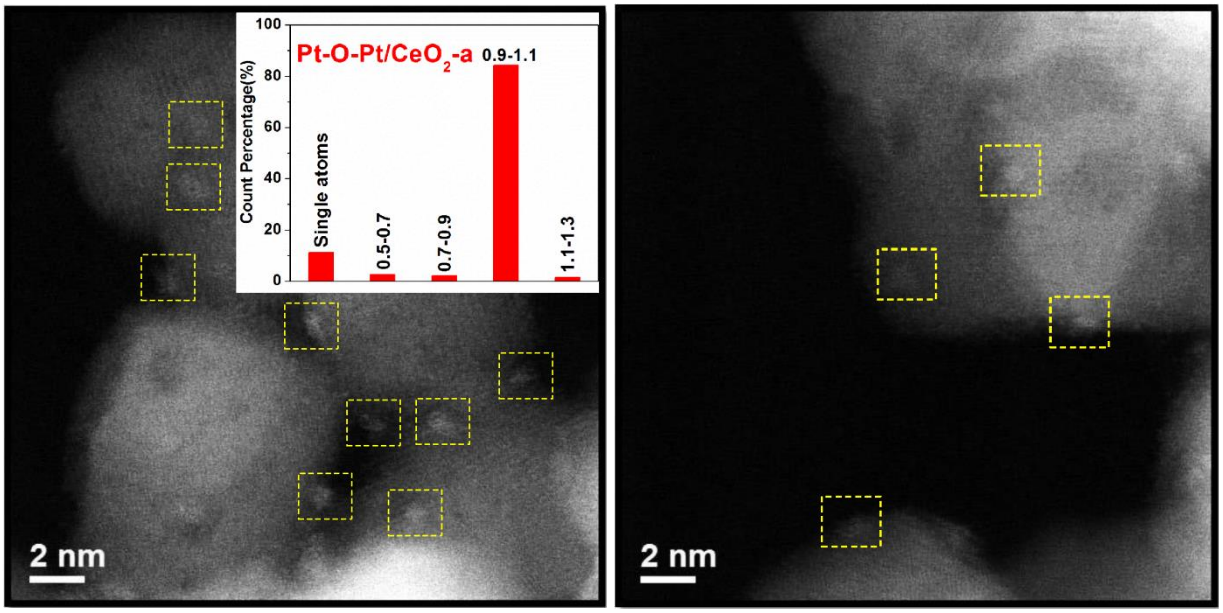

Supplementary Figure 11. HAADF-STEM images and the count frequency of Pt species in the Pt-O-Pt/CeO_2-a sample. Yellow squares are shown to label the ~1 nm species of Pt. The size distribution (inset, left) in nm is based on over 150 observed platinum species counted from the high-magnification images (images recorded at 6 M× original magnification).

Supplementary Note 4:

The visual heterogeneity of the size of platinum species from STEM images is due to three factors: 1) the heterogeneous nature of platinum structure itself; 2) the imaging distortion of size and shape of platinum species, as the platinum anchors on rather large ceria nanoparticles having uneven distribution of ceria particle shapes and surface steps; and 3) platinum atoms tend to be destabilized and become mobile under the influence of electron beam during the STEM analyses Therefore, similar platinum species will look somewhat different depending on the relative location of these species within one image.

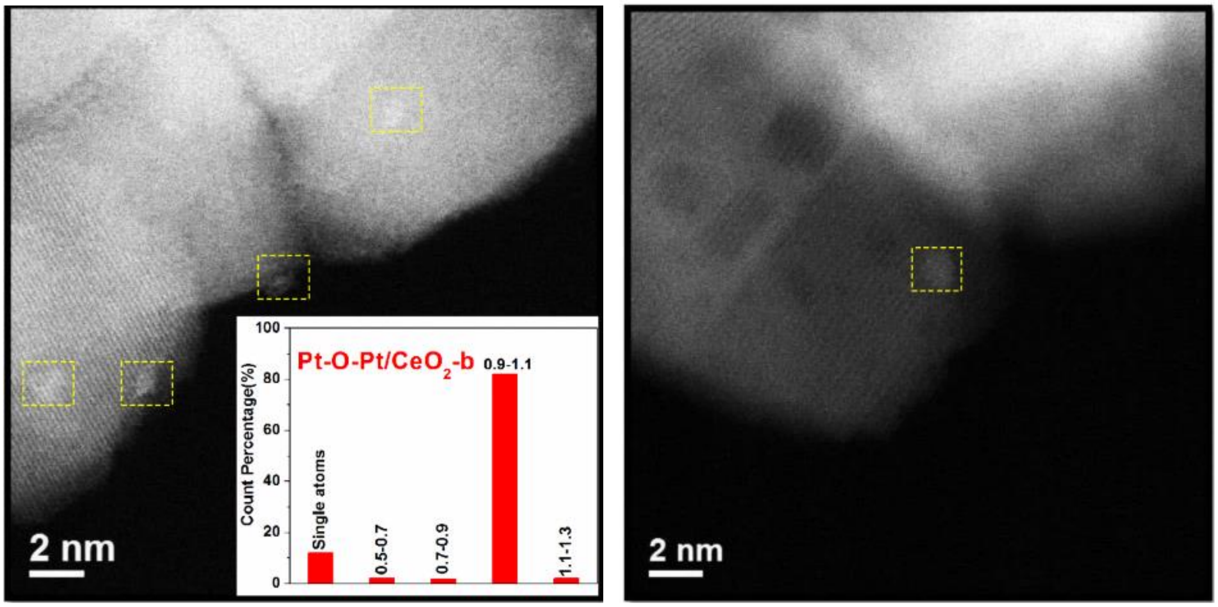

Supplementary Figure 12. HAADF-STEM images and the count frequency of Pt species in the Pt-O-Pt/CeO_2-b sample. The left and right images are two different representative views. Yellow squares are shown to label the ~1 nm species of Pt. The size distribution (inset, left) in nm is based on over 150 observed platinum species counted from the high-magnification images (both images recorded at 6 M× original magnification).

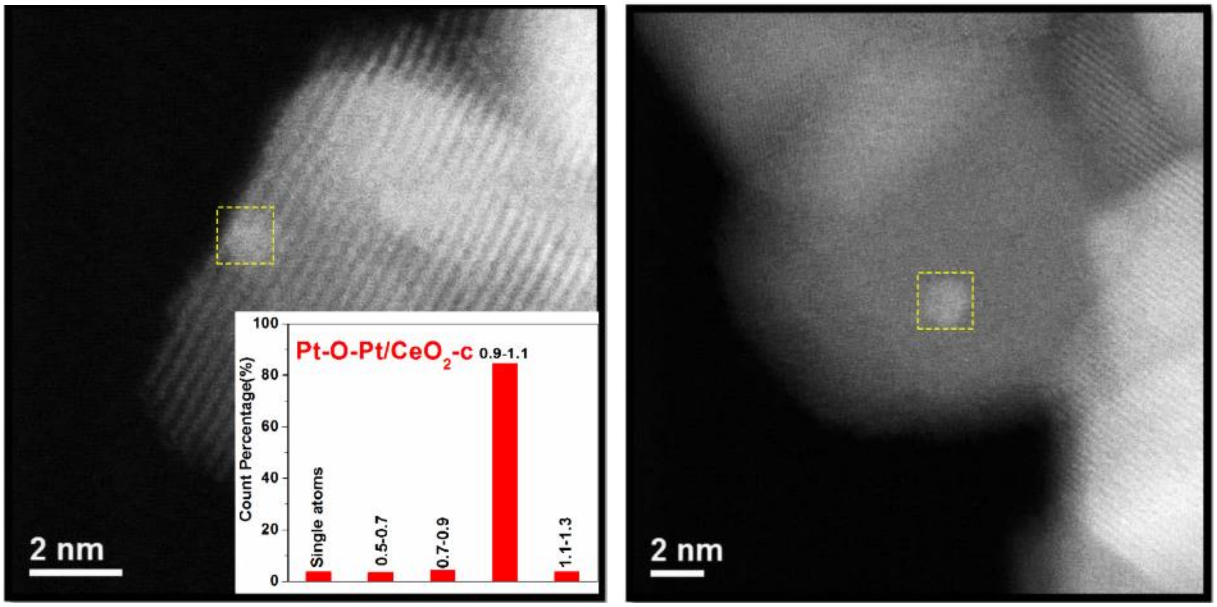

Supplementary Figure 13. HADF-STEM images and the count frequency of Pt species in the Pt-O-Pt/CeO_2-c sample. The left and right images are two different representative views. Yellow squares are shown to label the ~1 nm species of Pt. The size distribution (inset, left) in nm is based on over 150 observed platinum species counted from the high-magnification images (images recorded at 10 M× and 6 M× original magnifications, respectively).

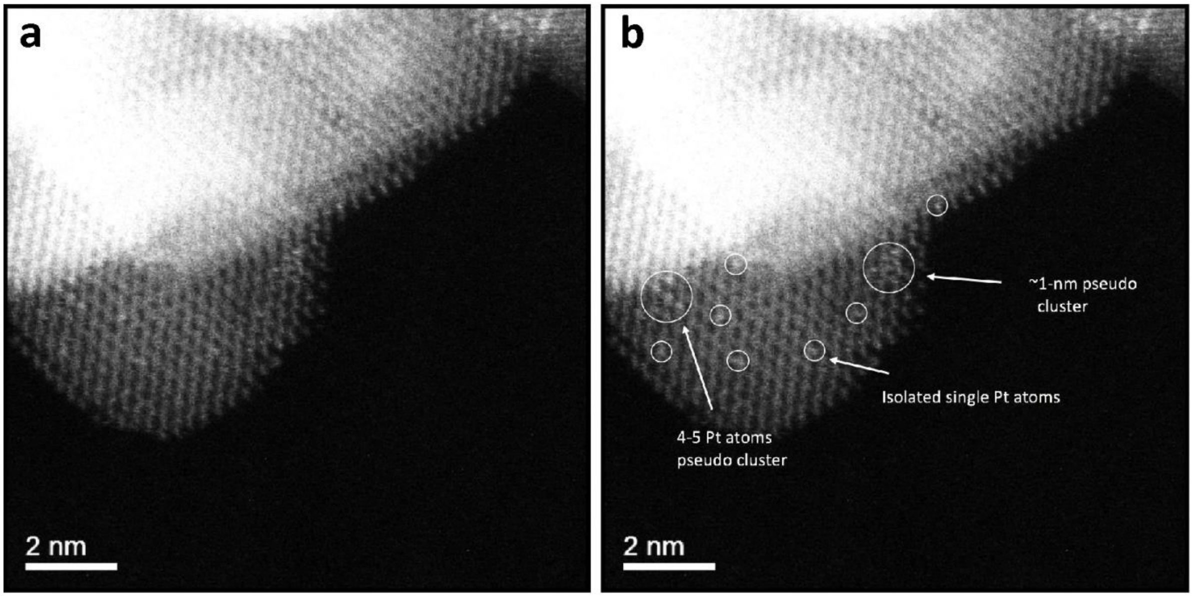

Supplementary Figure 14. Clearer visualization of the Pt_1 single atoms. Two representative HAADF-STEM images of the Pt_1/CeO_2-a sample show the pseudo clusters, consisting of nearby Pt_1 single atoms. **a** shows the contrast-enhanced raw image; **b** shows the identified pseudo clusters and isolated single atoms.

Supplementary Note 5:
As shown in Supplementary Figure 14, the Pt_1 is indeed the dominant species in the Pt_1/CeO_2 catalysts. The typical pseudo platinum clusters from sub-nm size up to 1 nm size have low abundance. The example of Supplementary Figure 14 shows HAADF images of two typical areas of the catalyst. The image pair is the same area, contrast-enhanced, with single atoms and two pseudo clusters identified in Supplementary Figure 14b. The pseudo cluster circled on the left side of Supplementary Figure 14a appears to be a few isolated platinum atoms that are substituting surface Ce atoms, as our DFT work has predicted. When STEM images were recorded with slight defocus on these platinum species due to the different heights of ceria planes, these platinum species tend to look similar to the true platinum clusters, but they are composed of isolated (non-contiguous) atoms.

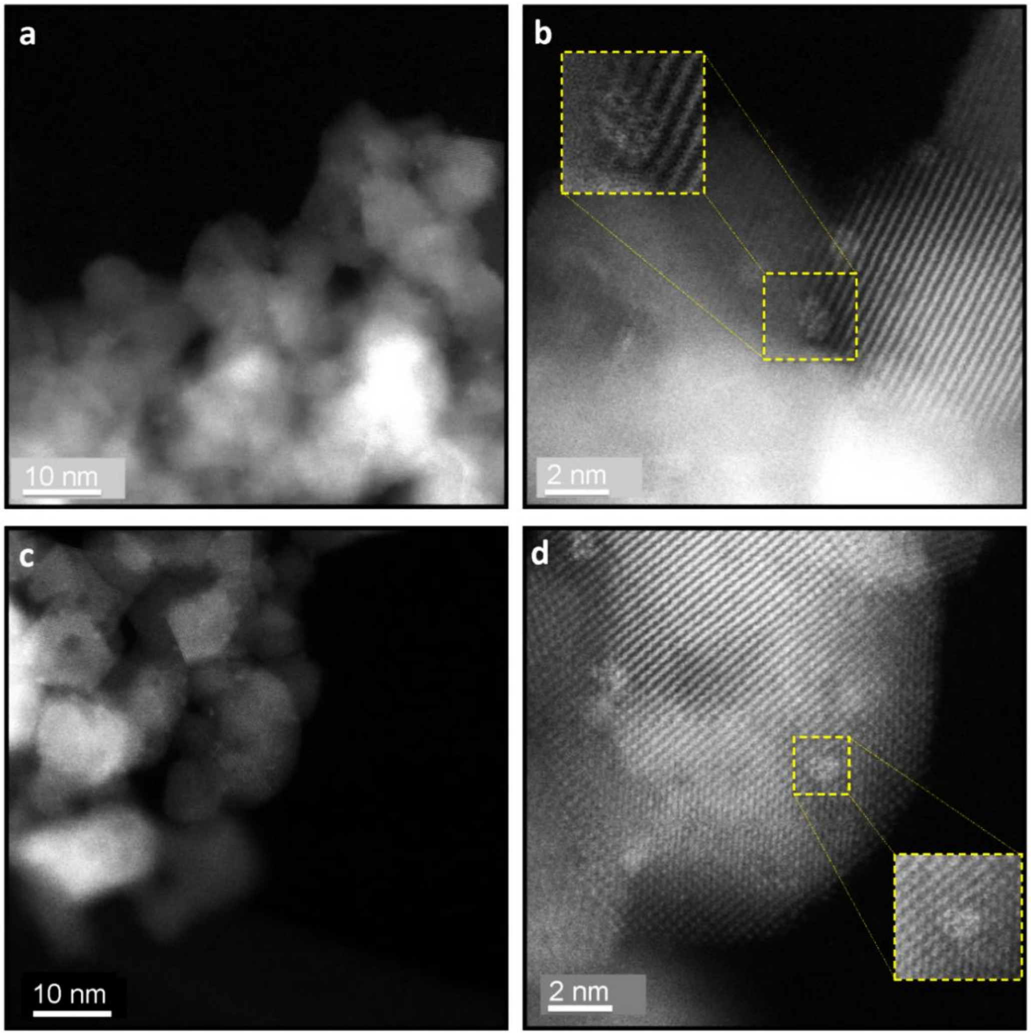

Supplementary Figure 15. Clearer visualization of the ~1 nm Pt-O-Pt ensembles. Representative HAADF-STEM images of the Pt-O-Pt/CeO_2-a sample with low (a, c) and high (b, d) resolutions give clearer visualization of the ~1 nm Pt-O-Pt ensembles.

Supplementary Note 6:

The synthesized Pt-O-Pt ensemble in the activated Pt-O-Pt/CeO_2 catalysts is different from those pseudo clusters present in the Pt_1/CeO_2 samples. As shown in Supplementary Figure 15, the Pt-O-Pt ensembles dispersed over the catalyst support show a narrow size distribution around 0.9 to 1.1 nm, and these structures tend to be anchored on top of the ceria substrate rather than being embedded in the cerium column as shown for the Pt_1/CeO_2-a sample (Supplementary Figure 14). The inset in Supplementary Figure 15 indicates that there are multiple platinum atoms in the typical Pt-O-Pt ensembles. However, the precise counts of platinum atoms in each ensemble cannot be determined with high precision as the platinum atoms tend to be destabilized and move around under the influence of electron beam during the STEM analyses. We therefore conducted the DFT work to predict the most plausible stable platinum structure (~1 nm) under the conditions reflecting the experimental work.

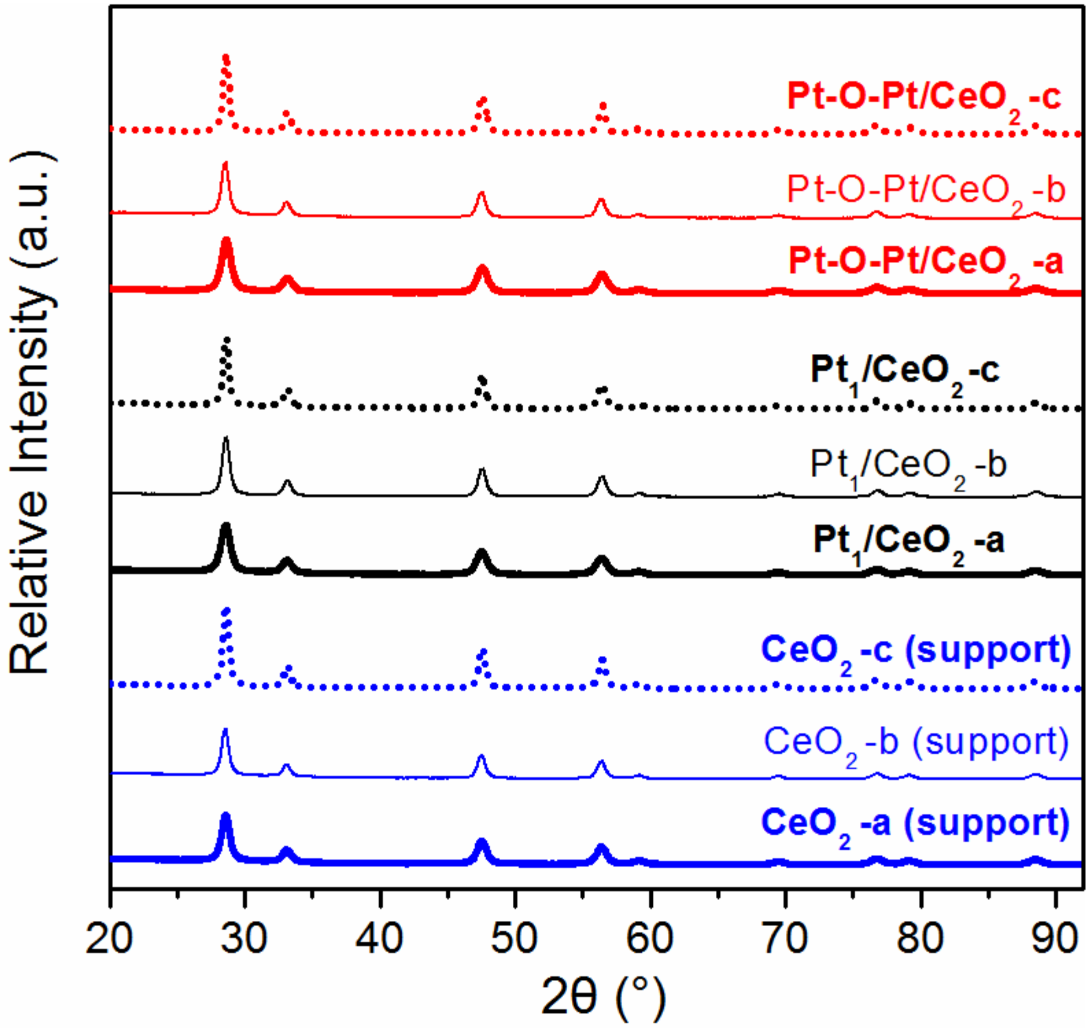

Supplementary Figure 16. XRD patterns for the Pt-free ceria materials and the Pt-loaded Pt/CeO_2 catalysts. Only fluorite cubic structures of ceria support are detected, and CeO_2(111) is the dominant facet for all Pt/CeO_2 catalysts. Higher heating treatment temperatures for the ceria supports from CeO_2-a to CeO_2-c resulted in larger ceria particle size as indicated by higher peak intensities of the fluorite cubic structures. The ceria particle size ranges from 10 to 30 nm according to XRD calculations based on Scherrer equation.

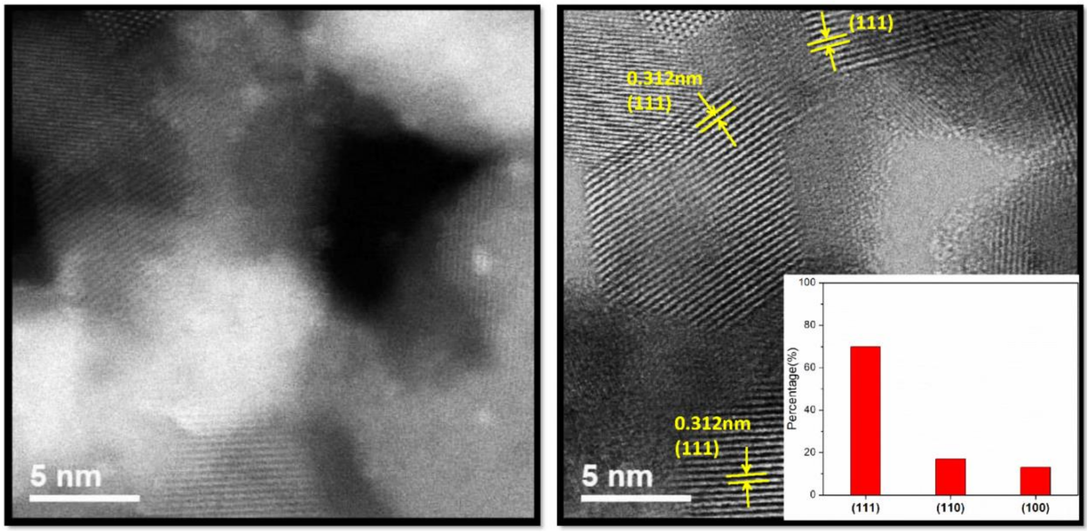

Supplementary Figure 17. HADDF-STEM and the corresponding bright-field image of the Pt-O-Pt/CeO_2-a sample. The count frequency of ceria crystal plane types (inset) was based on over 100 focused surfaces of Pt/CeO_2-a, -b, -c samples used in this study, on which Pt single atoms (in as-prepared catalyst) or ~1 nm Pt-O-Pt species were anchored. It was observed that the amounts of Pt species anchored on CeO_2(111), (110), and (100) facets are *ca.* 70, 17, 13 %, respectively. Images were recorded simultaneously at 5 M×.

Supplementary Note 7:

Our experimental evidence from XRD (Supplementary Figure 16, overall crystal surfaces) and STEM (Supplementary Figure 17, crystal surfaces where the platinum species reside) confirmed that the CeO_2(111) is the predominant ceria surface on which the platinum species anchor themselves, since all our ceria support particles have experienced calcination from 600 to 750 ^oC (see Catalyst preparation in Supplementary Information). This observation is in line with stability analysis using *ab initio* atomistic thermodynamics, for which the most stable surface structure was predicted to be the stoichiometric (111) surface under "oxygen-rich" conditions among the considered CeO_2(111), (100) and (110) facets[36]. Performing our modeling studies of the platinum structures on representative CeO_2(111) reflects our experimental observation that 70 % of both Pt_1 single atoms and Pt-O-Pt ensembles were present on CeO_2(111) (Supplementary Figure 17). A minority of 17 and 13 % of Pt species were found on the CeO_2(110) and CeO_2(100) surfaces (Supplementary Figure 17), respectively. We therefore decide to perform a majority of our modeling studies of the platinum structures over the CeO_2(111) surface. Although surface science studies[26,27,37] and theory work[26,38] have shown that the direct contact of Pt and CeO_2(111) is more favorable than ceria alone in activating ceria lattice oxygen and consequently in promoting the CO oxidation reaction, other experimental studies involving deposited platinum on ceria nanoshapes[15,39-41] and theory studies adopting nanoparticles other than slabs[42,43] show that the more reactive oxygen species may exist on CeO_2(110) and (100) planes.

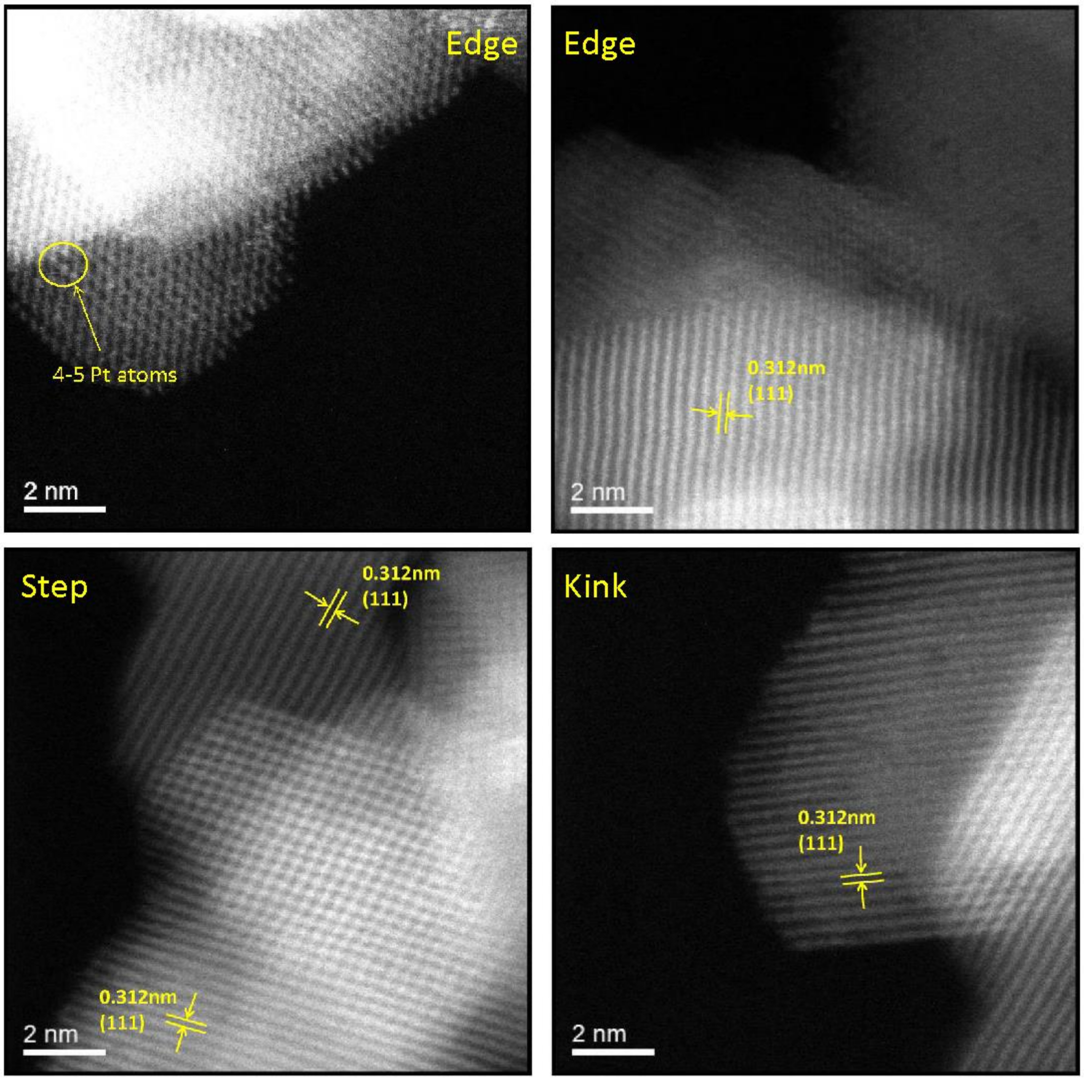

Supplementary Figure 18. Clearer visualization of the Pt_1/CeO_2-a sample. HAADF-STEM images of the Pt_1/CeO_2-a sample show the rounded edges, steps, and kinks on ceria particles.

Supplementary Note 8:

Our CeO_2-a, -b, -c are hydrothermally treated at 600, 700 and 750 °C for 5 hrs respectively before Pt loading, and our ceria support particle sizes are within the typical reported ranges (10–30 nm), which has an abundance of stable CeO_2(111) facets[44,45]. Due to the preparation method we adopted, in which the platinum-ligands complex anions can adsorb on any positively charged –O-Ce-OH_2^+ surface sites as a single-atom platinum complex, we do not observe preferred platinum anchoring on those edges, steps and kinks (Supplementary Figure 18) similar to what would happen by using < 5 nm ceria clusters exposing small O-terminated (111) and (100) facets[42,46-48], where a much more facile oxygen activation can happen through the Pt-CeO_2 interface to catalyze the CO oxidation reaction.

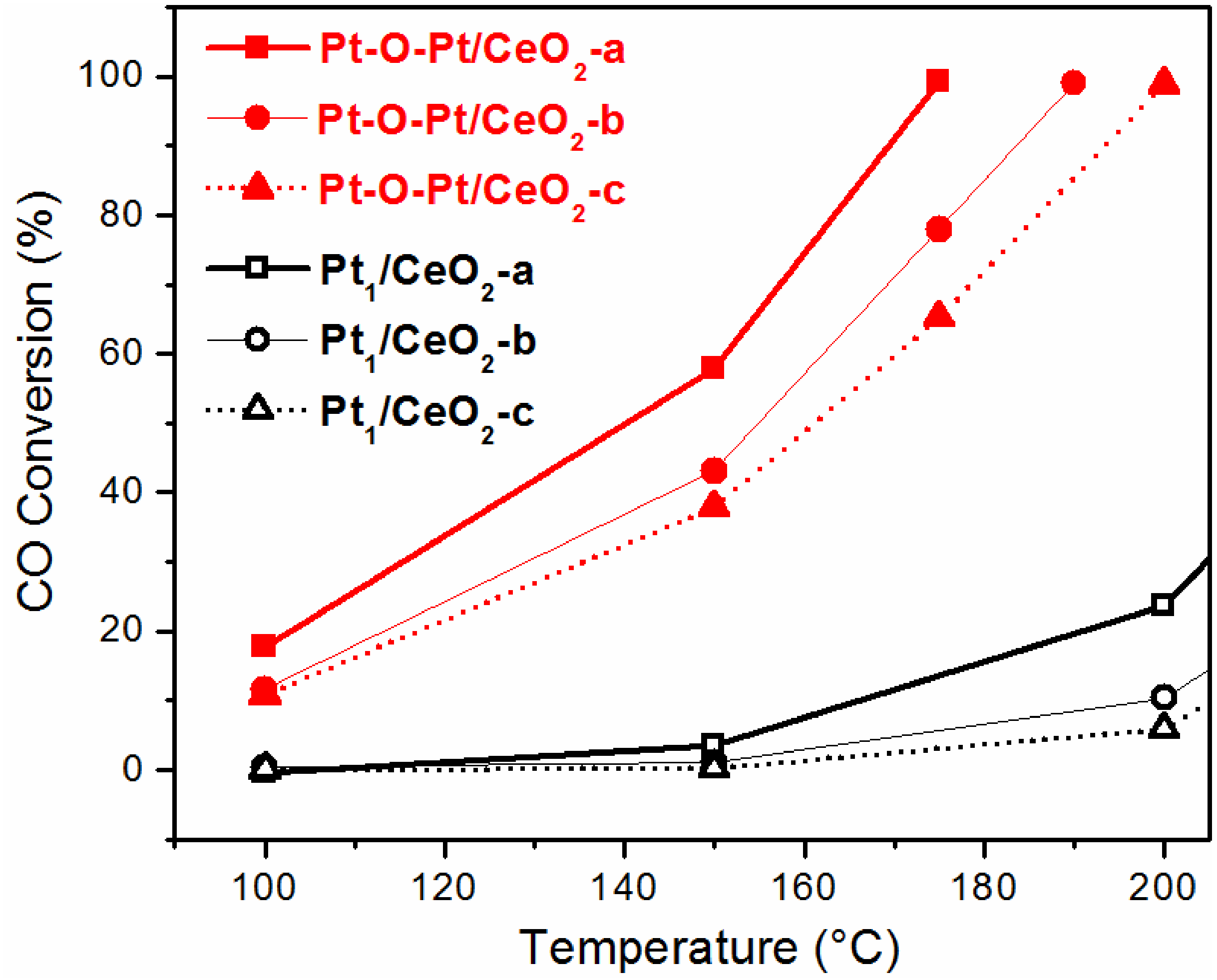

Supplementary Figure 19. CO oxidation test results for the complete series of the Pt_1/CeO_2 and Pt-O-Pt/CeO_2 catalysts. CO oxidation conditions: [CO] = 1000 ppm, [O_2] = 5 % balanced with N_2 at a contact time of 2,400,000 ml g_{cat}^{-1} hour^{-1}.

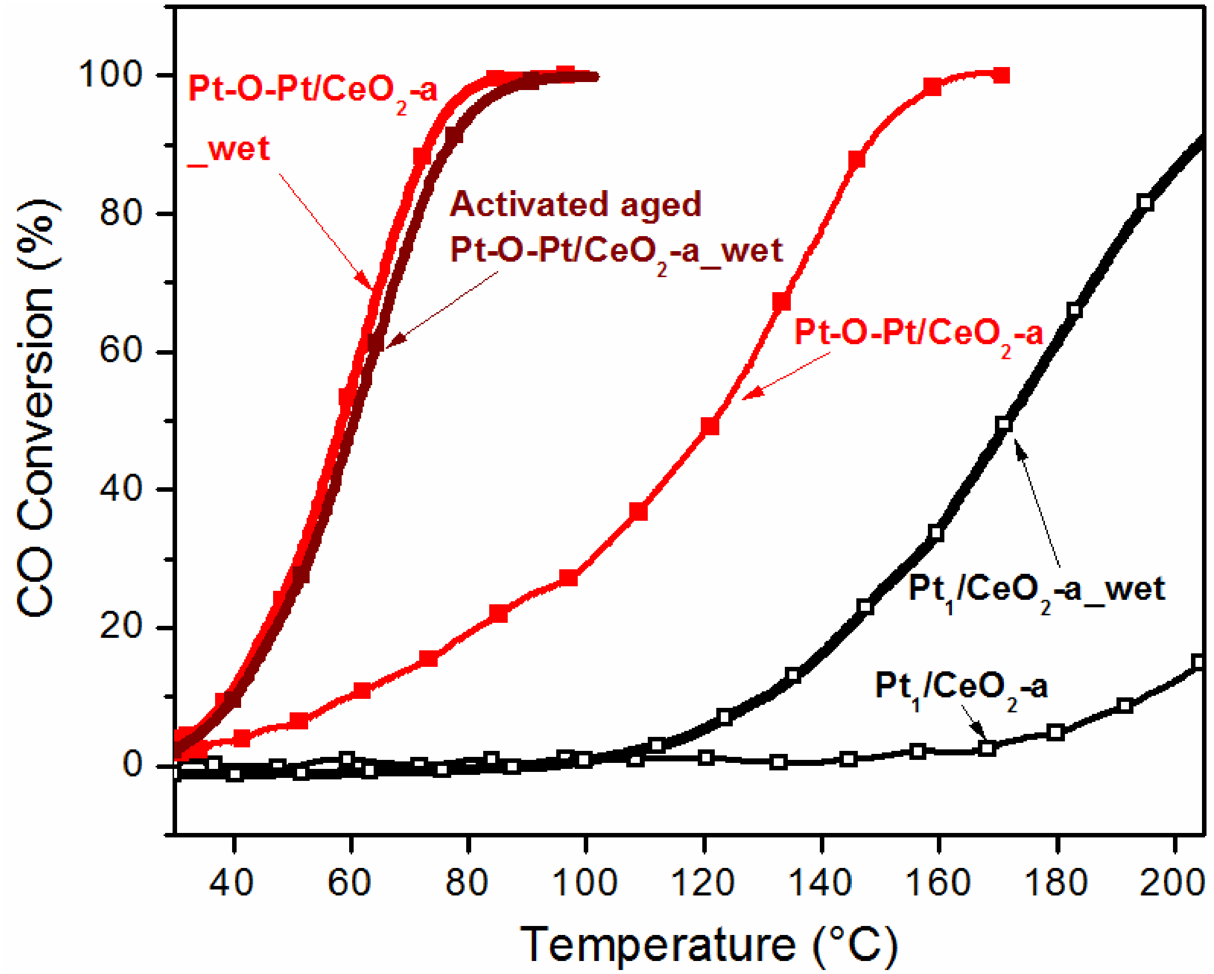

Supplementary Figure 20. CO oxidation with and without water in the feed gas for the as-prepared and hydrothermally aged catalysts. Catalyst aging condition: 750 °C for 20 hrs, 10 % H_2O in air. CO oxidation: [CO] = 1000 ppm, [O_2] = 5 %, with or without 3 % H_2O, balanced with N_2 at a contact time of 2,400,000 ml g_{cat}^{-1} hour^{-1}.

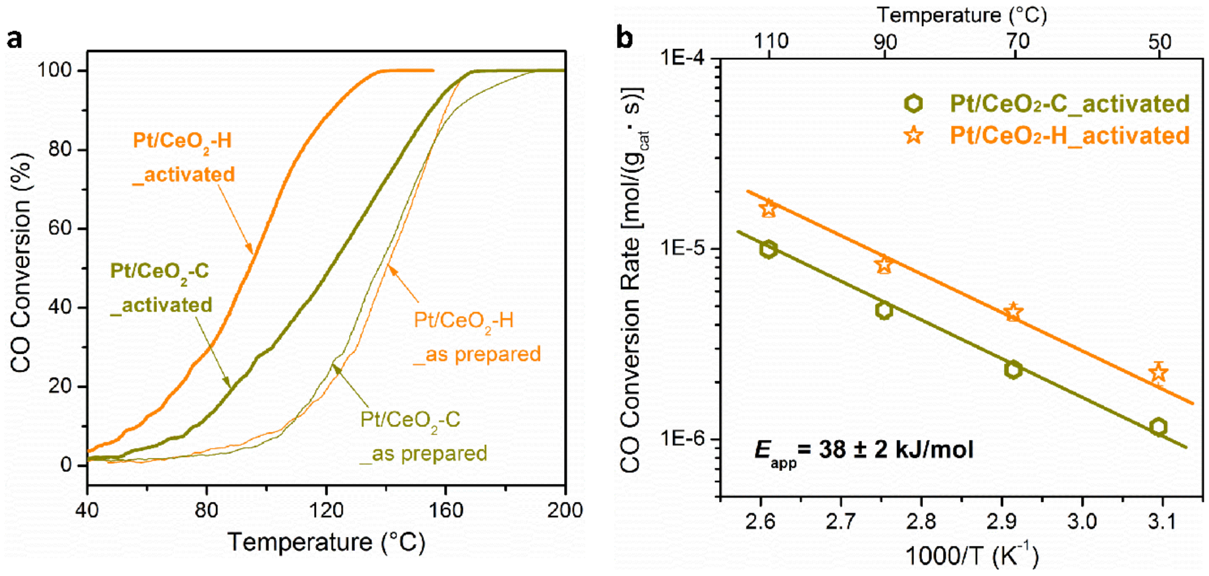

Supplementary Figure 21. Generalizability of the activation protocol. CO oxidation of (a) light-off performance and (b) Arrhenius-type plots for the Pt/CeO_2-C and Pt/CeO_2-H catalysts using commercial ceria supports. The reaction rates were measured within the kinetically controlled region, [CO] = 1000 ppm, [O_2] = 5 %, balanced with N_2. Activation: reduced at 200 °C in 5 % H_2 for 15 minutes and exposed to CO plus O_2 atmosphere at ambient temperature. The activation approach is effective to improve the light-off activity of the commercial catalysts. The E_{app} values around 38 kJ/mol indicates that the same catalytic center may have been created as in those Pt-O-Pt/CeO_2 catalysts.

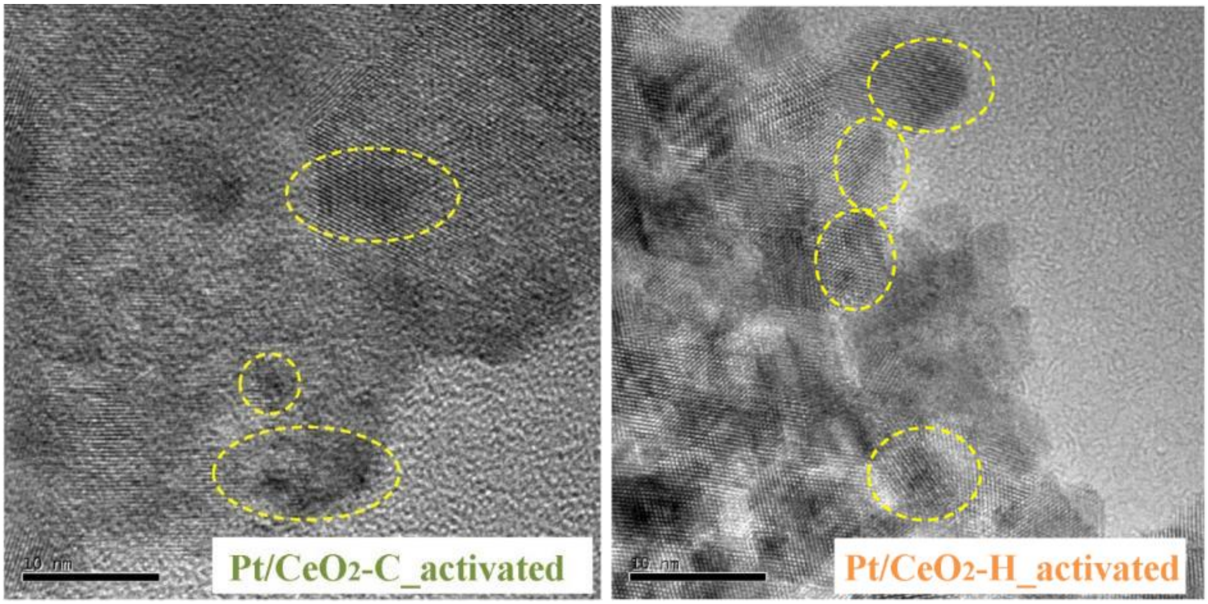

Supplementary Figure 22. HRTEM images for the Pt/CeO_2-C and Pt/CeO_2-H samples using commercial ceria supports. The scale bar on both images is 10 nm. Reflecting the typical surface morphology of commercial catalysts, large platinum nanoparticles (possible domains labeled by dashed circles) are formed due to the high Pt loading amount and suboptimal PGM loading method. The presence of those large metal particles leads to PGM waste and thus lower reactivity per mole of supported platinum, if compared with those optimized Pt-O-Pt/CeO_2 catalysts developed in our lab.

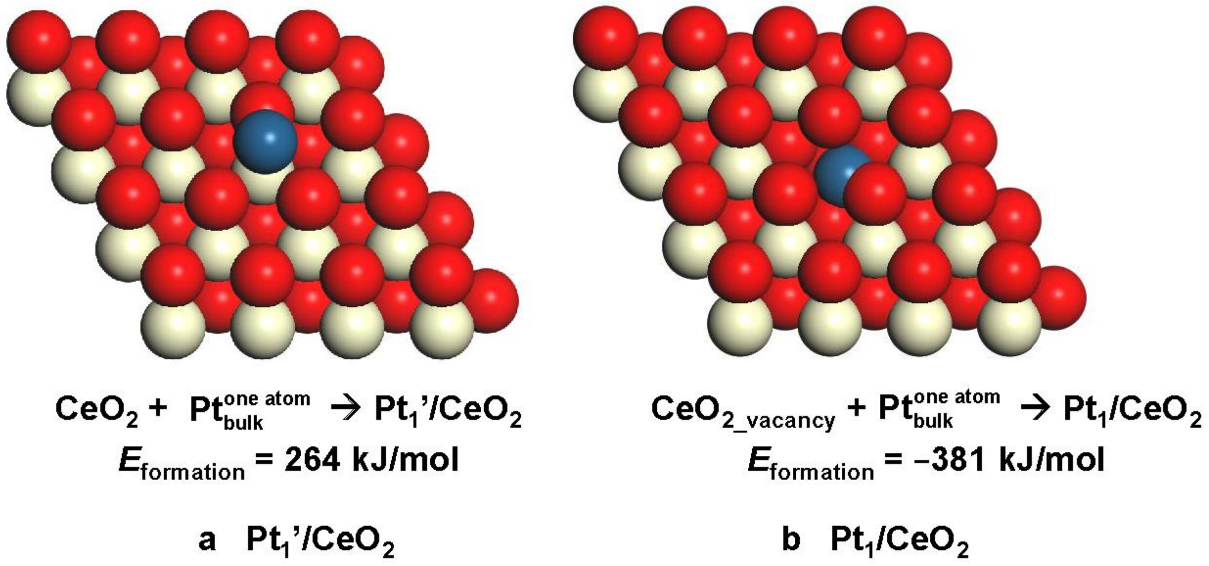

Supplementary Figure 23. Optimized structures of the single-atom catalyst. Optimized structure and formation energy ($E_{formation}$) of Pt single atom on CeO_2(111) (Pt_1'/CeO_2, **a**) and Pt single atom substituted in CeO_2(111) (Pt_1/CeO_2, **b**). For Pt_1'/CeO_2, the single Pt atom prefers to bind with one O and one Ce atom and has a coordination number of two. The Pt_1/CeO_2 is more stable than Pt_1'/CeO_2 due to the formation of four Pt-O bonds in Pt_1/CeO_2. Atom color legend: Beige spheres = Ce; Red spheres = O; Blue spheres = Pt. This notation is used throughout the supplementary information.

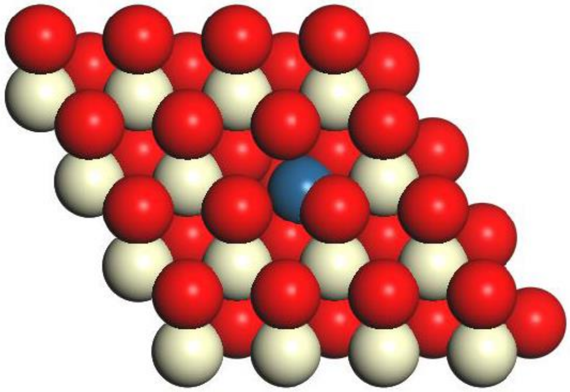

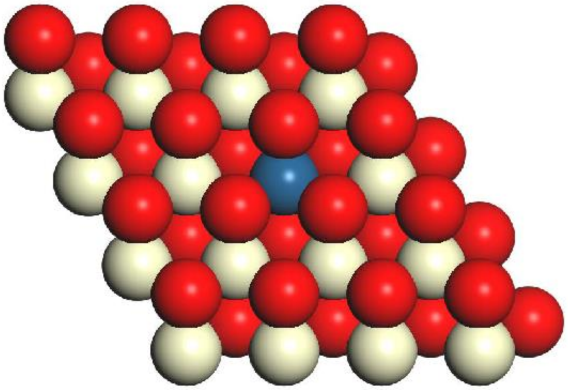

Supplementary Figure 24. Optimized structures of the Pt single atoms substituted in CeO_2(111). The Pt single atom binds four and six O atoms in the Pt_1/CeO_2 (left, **a**) and the Pt_1''/CeO_2 (right, **b**) structures, respectively. Pt_1/CeO_2 and Pt_1''/CeO_2 have similar stability and Bader charge of Pt atom. The numbers under the structures are relative electronic energies with respect to that of Pt_1/CeO_2.

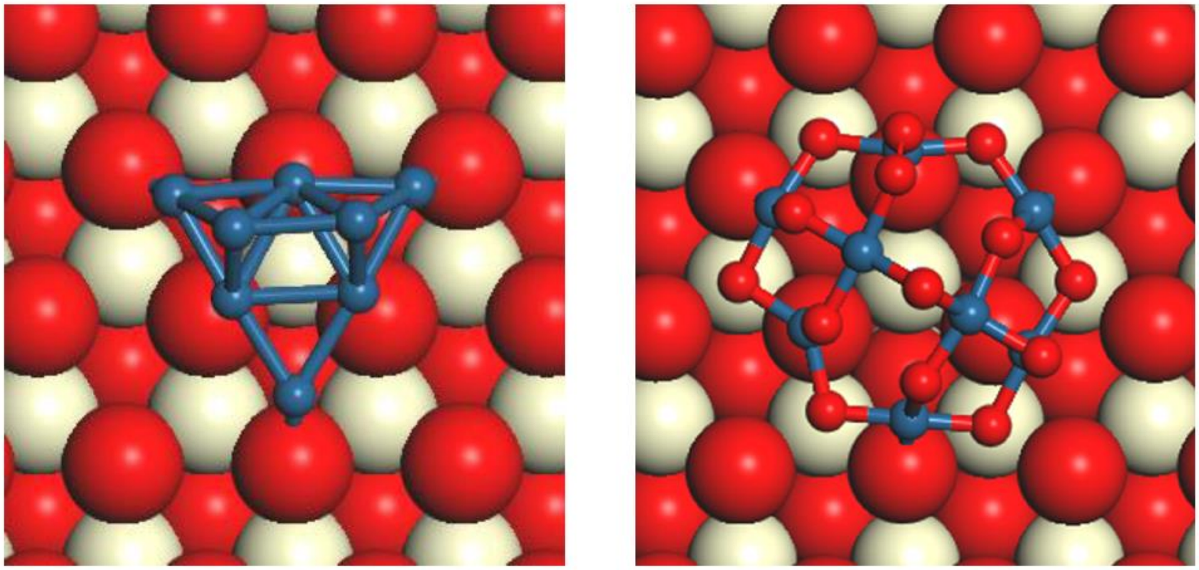

a Pt_8/CeO_2 **b** Pt_8O_{14}/CeO_2

Supplementary Figure 25. Optimized structures of the Pt_8/CeO_2 and Pt_8O_{14}/CeO_2 model systems. The structures of (**a**) Pt_8/CeO_2 and (**b**) Pt_8O_{14}/CeO_2 searched by the genetic algorithm and the grand-canonical Monte Carlo simulations, respectively.

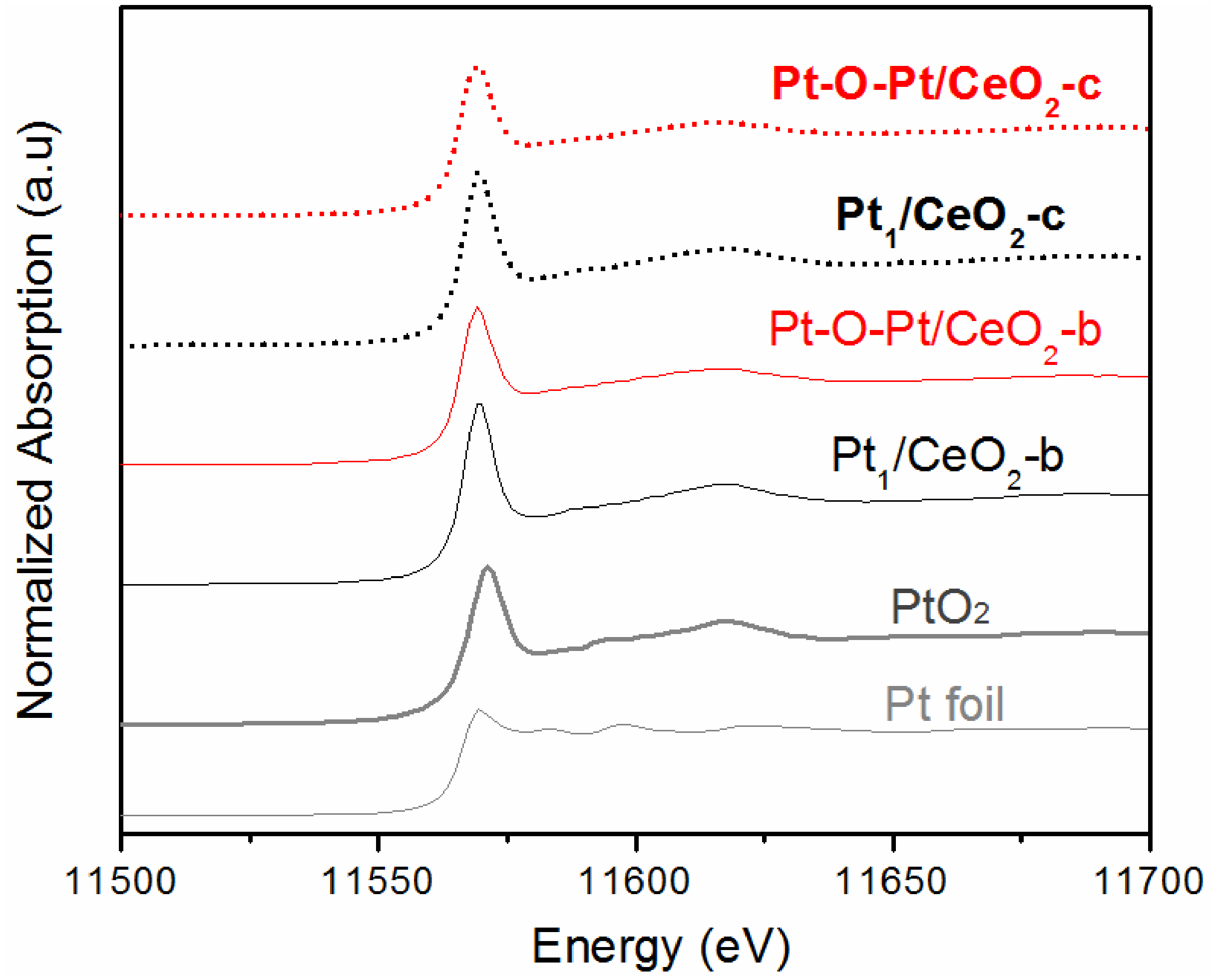

Supplementary Figure 26. Normalized Pt L_3 edge XANES spectra for the Pt_1/CeO_2-b, Pt_1/CeO_2-c, Pt-O-Pt/CeO_2-b, and Pt-O-Pt/CeO_2-c catalysts. The PtO_2 standard is in β phase.

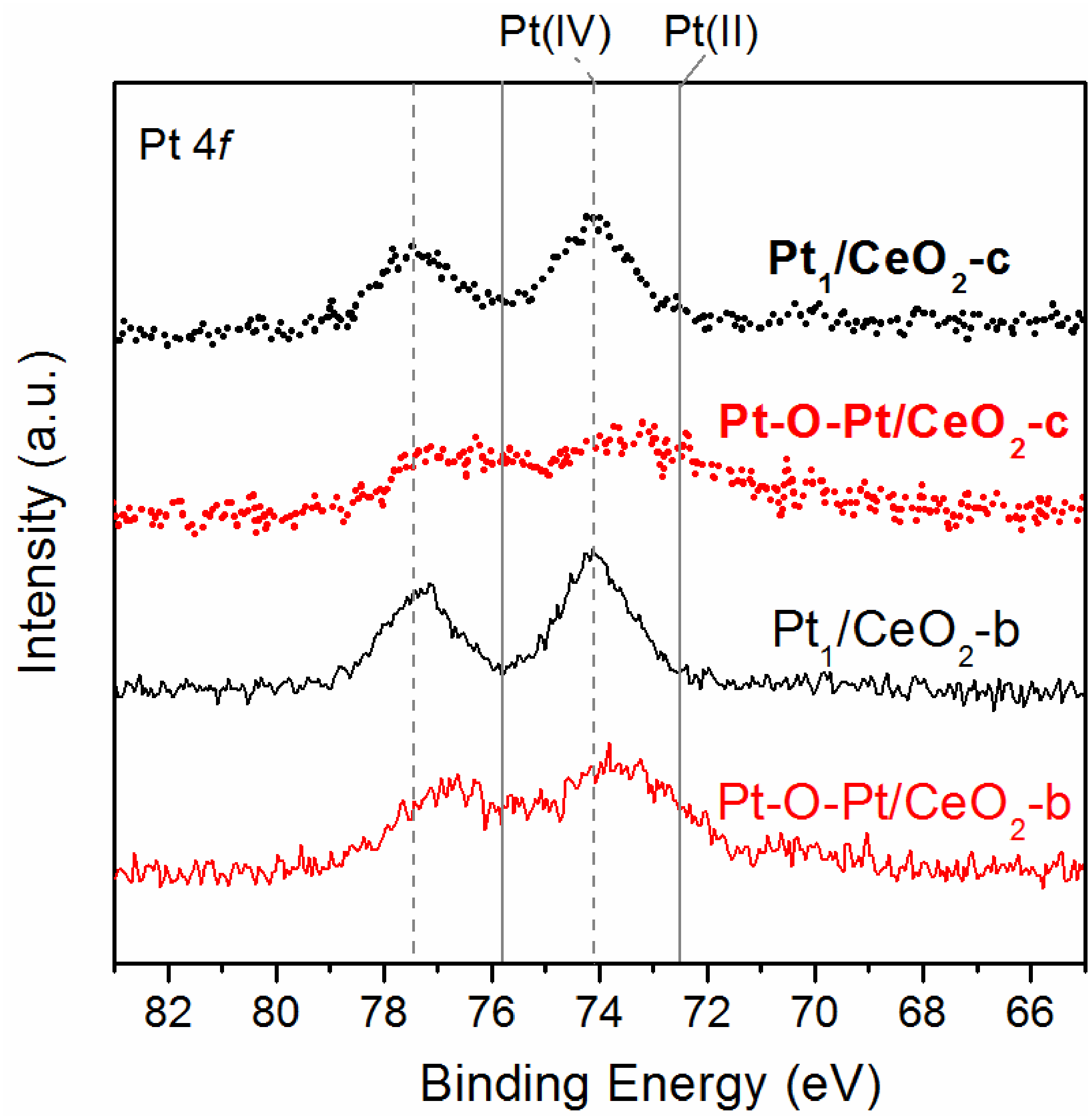

Supplementary Figure 27. Pt 4*f* XPS spectra for the Pt_1/CeO_2-b, Pt_1/CeO_2-c, Pt-O-Pt/CeO_2-b, and Pt-O-Pt/CeO_2-c catalysts. The PtO_2 standard is in β phase.

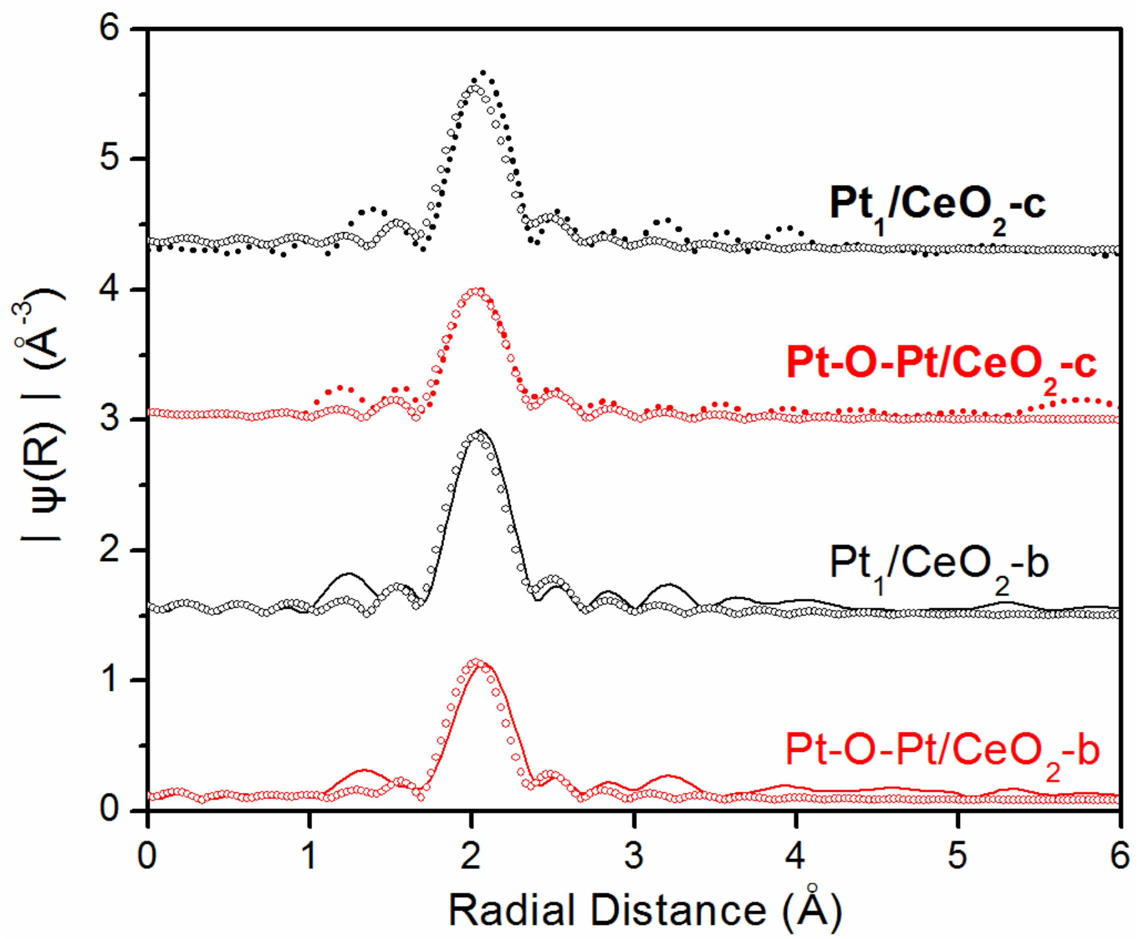

Supplementary Figure 28. Fourier transform of EXAFS spectra of Pt L_3 edge for the the Pt_1/CeO_2-b, Pt_1/CeO_2-c, Pt-O-Pt/CeO_2-b, and Pt-O-Pt/CeO_2-c catalysts. These spectra are phase corrected. The 1st shell Pt-Pt coordination is not observed in the Pt_1/CeO_2 and Pt-O-Pt/CeO_2 catalysts. The grey and red open circles are fitted curves for the Pt_1/CeO_2 and Pt-O-Pt/CeO_2 catalysts, respectively.

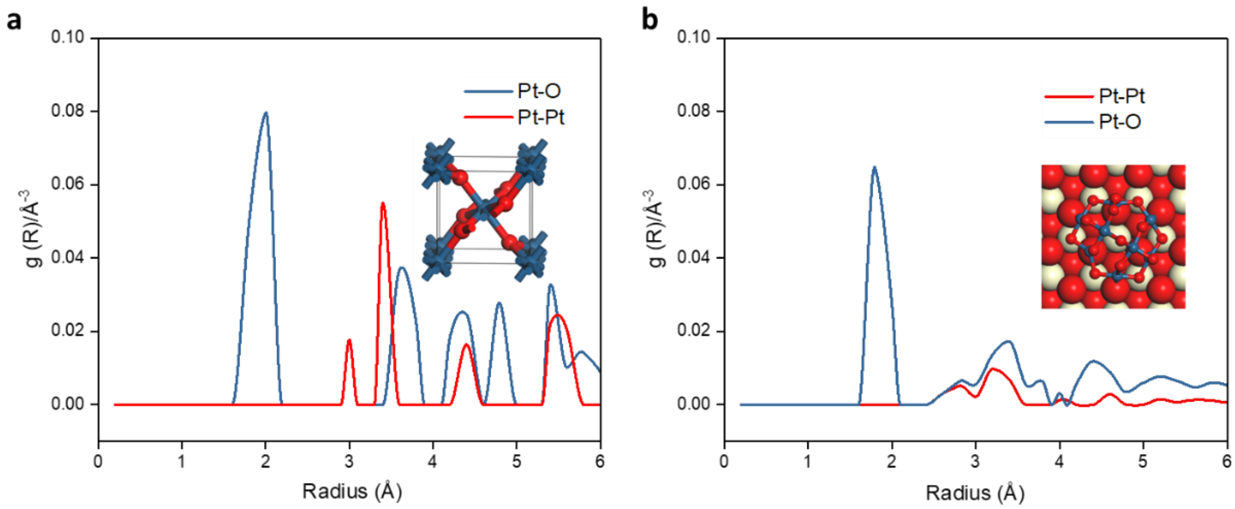

Supplementary Figure 29. Radial distribution function calculations for β-PtO_2 and Pt_8O_{14}. Simulated radial distribution function g(R) normalized to one Pt atom in the (**a**) β-PtO_2 crystalline standard and the (**b**) Pt_8O_{14} on ceria.

Supplementary Note 9:

It is typical that the EXAFS χ(*k*) signal decays quickly with *k*, and this limitation can be somewhat mitigated after increasing the signal weight for high *k* portion (e.g., k^2 and k^3) and performing Fourier Transform to generate the function as χ(R), which was used in the presentation of this work. This treatment is especially reliable in generating well-separated first-shell peaks for simple crystalline metal-oxide and metal systems, where the bond length between the metal center and its first shell oxygen or secondary metal is well below 3 Å and can be isolated from other shell overlaps and instrumental scattering signal attenuation at higher radial distances (i.e., R > 3 Å). Beyond the quantified analyses of the first shell metal-oxygen coordination conditions, we must avoid overinterpretation of observations made from the high R range, even for simple materials. This issue has been documented in a variety of XAS studies[49].

We begin the discussion by showing the β-PtO_2 crystalline standard that we used as a reference in the manuscript. Extensive studies in structural chemistry have been done on the β-PtO_2 crystal, and the theoretical bonding conditions of the crystal are well understood[50]. Supplementary Figure 29a visualizes the predicted radial distribution function normalized to one central platinum atom by fully adopting the crystal unit cell parameters of the β-PtO_2 through our theoretical modeling work. Compared with our collected data, the major signal attenuation at higher radial distances (R > 3 Å) in the experiments is obvious and in agreement with what many others have found. Precise data fitting can be only conducted for the 1st shell Pt-O coordination in this pure powder reference.

Considering the yet successful examples for the crystalline β-PtO_2 standard, it is expected that trying to obtain coordination information in the 3–4 Å radial distance range from the 0.11–0.27 wt.% Pt-O-Pt ensembles dispersed on the high surface area ceria supports will be even more challenging and unreliable. Besides the inherent experimental signal attenuation, the one-layer

structure and the dilute concentrations of the Pt-O-Pt ensemble contribute to the lack of useful information from the experimental 3–4 Å radial distance range.

Supplementary Figure 29b shows the radial distribution function normalized to one central platinum atom by fully adopting the geometric information predicted by our computational studies in finding the Pt_8O_{14} structure. The average first shell Pt-O bond length is 1.99 Å with the same coordination number of four for each platinum atom. These results agree with our experimental EXAFS measurement and fittings done in the radial distance below 3 Å (Supplementary Table 3). At higher radial distances, due to the lack of three-dimensional structure, the predicted scattering signals above 2.5 Å in R-space for the Pt_8O_{14} (even as undiluted species) is much lower than the radial distribution simulation result for the crystalline β-PtO_2 standard (Supplementary Figure 29a). As expected, the same trend is shown in the experimental data, where the predicted long-distance Pt–Pt with an average distance of 3.22 Å and a rather small coordination number of 3 to 4 can hardly be detected. The dilute platinum concentrations (0.11–0.27 wt.%) of our actual catalysts further contribute to the dampening of the already weak signal at high radial distances. In fact, it is not a rare case that EXAFS experimental data alone cannot provide direct evidence of long-distance Pt–Pt scattering that should exist in the supported small platinum oxide clusters. For instance, such a long-distance Pt–Pt feature is absent in the 1.3 wt.% PtO_x sub-nm clusters supported on ceria nanowires[51]. The absence of the long-distance Pt–Pt feature in the 1.0 ± 0.5 nm Pt_nO_x clusters with platinum loadings of 0.3 and 0.9 wt.% was also reported in the Pt/Al_2O_3 catalysts[52], which is supposed to have much less influence from oxygen bonds of the inert support. Even for those 1 wt.% Pt/Al_2O_3 catalysts with an average Pt particle size around 2.0 ± 0.5 nm, the long-distance Pt–Pt feature in the 3–4 Å range is still largely missing under EXAFS analyses[53].

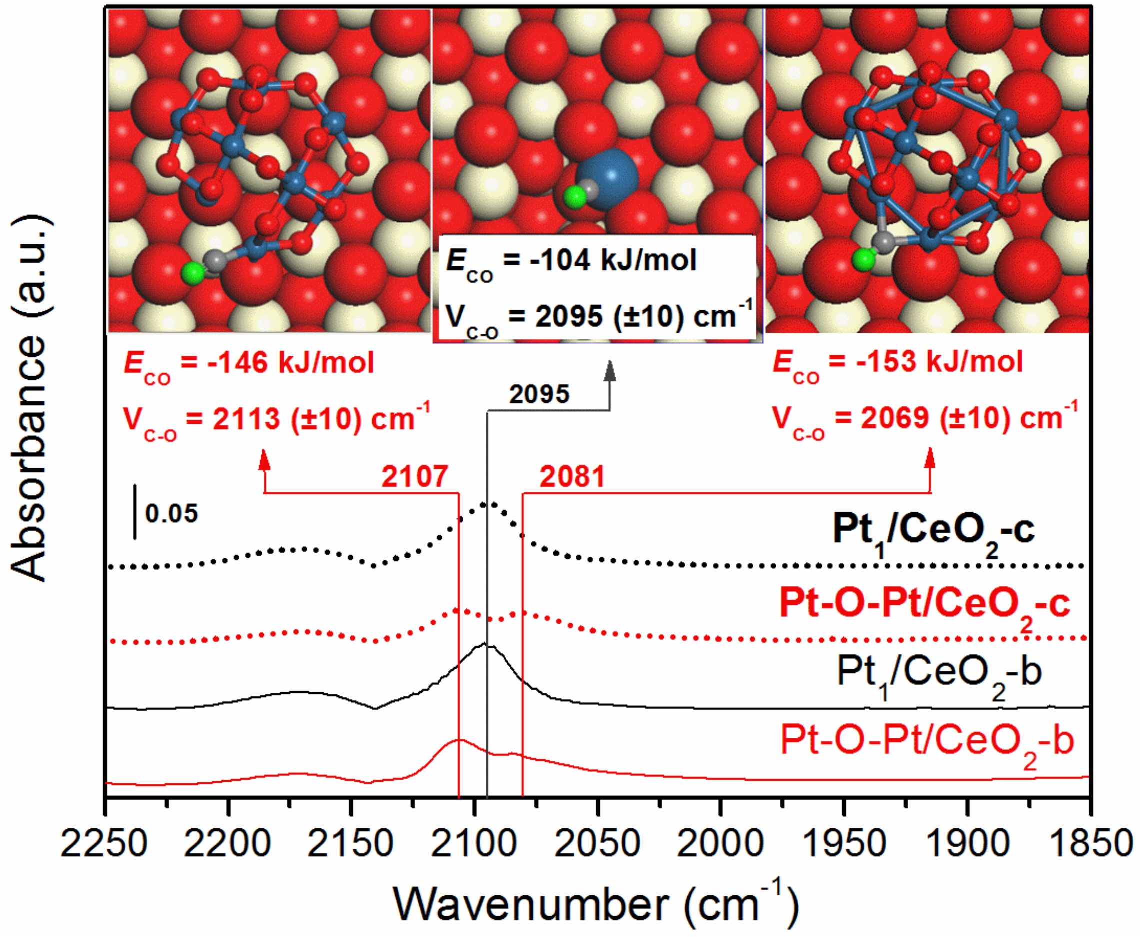

Supplementary Figure 30. ***In situ*** **DRIFTS under CO oxidation conditions for the Pt_1/CeO_2-b, Pt_1/CeO_2-c, Pt-O-Pt/CeO_2-b and Pt-O-Pt/CeO_2-c catalysts.** DFT-predicted CO adsorption modes are shown inset for Pt_1/CeO_{2-x} and Pt_8O_{13}. Beige, red, and blue spheres are Ce, O and Pt atoms, respectively. The small grey and green spheres are C and O atoms involved in CO oxidation, respectively.

Supplementary Note 10:

The FWHM of the experimental infrared (IR) peaks are in the range of 25–30 cm^{-1}, indicating the minor heterogeneity of the platinum properties due to the random nuances of local environment in the catalysts. The values of FWHM in our case are in line with other recent reports focusing on developing applied catalysts that show atomic precision of the platinum group metal (PGM) sites[26,54,55]. For truly exclusive catalytic sites, the FWHM should be in the range of 6–8 cm^{-1} [56], which is difficult to achieve if one hopes to keep appreciable amount metal loading on industrial catalyst supports. Overall, while we acknowledge the deficiency, it is safe to conclude that the experimental work effectively creates a single-dominant catalytic species, and reasonably bridges the gap between the industrial-relevant catalysts and theoretical modeling.

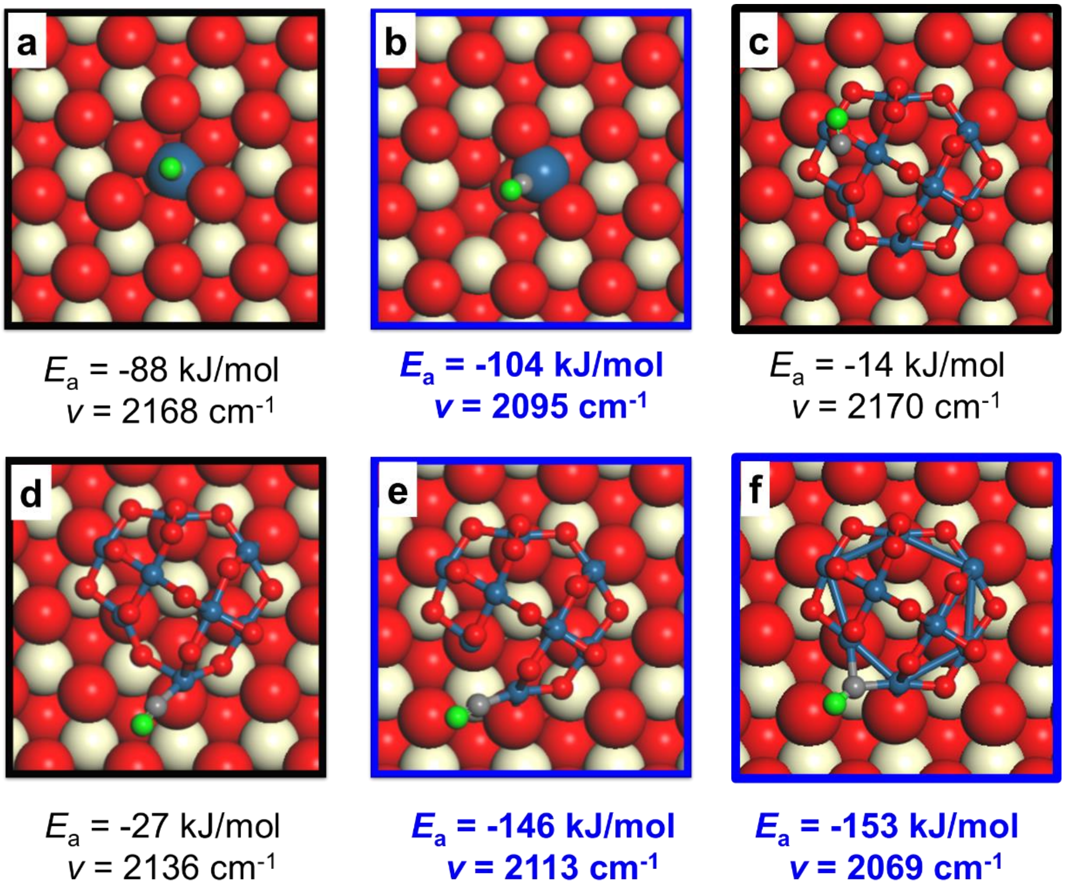

Supplementary Figure 31. CO adsorption energy and vibrational frequency calculation. Calculated CO adsorption energies and corresponding vibrational frequencies on **a** Pt_1/CeO_2, **b** Pt_1/CeO_{2-x}, **c** and **d** Pt_8O_{14}/CeO_2, **e** Pt_8O_{13}/CeO_2 and **f** Pt_8O_{12}/CeO_2 structures**.** All the energies and frequencies are in kJ/mol and cm^{-1} units, respectively. The bold blue text indicates CO adsorption modes on Pt_1/CeO_{2-x} and Pt_8O_{13}/CeO_2 structures comparable with experimental DRIFTS measurement.

Supplementary Note 11:

On Pt_1 substituted in $CeO_2(111)$, the calculated CO adsorption energy and vibrational frequency are −88 kJ/mol and 2168 cm^{-1}, respectively. The strong CO adsorption is caused by the large lattice reconstruction and the high CO vibrational frequency cannot be assigned as the CO adsorption mode in our experimental DRIFTS spectra. However, after CO reacts with lattice O in Pt_1/CeO_2 samples, a subsequent CO adsorbs on the Pt atom strongly with an adsorption energy of −104 kJ/mol (Supplementary Figure 31b), followed by CO reacting with lattice oxygen in CeO_2 and O_2 dissociation to complete the catalytic cycle. At 385 K, our microkinetic simulation predicts the coverage of CO with this adsorption mode is about 0.99, which should be observable in DRIFTS experiments. Supporting this hypothesis, our calculated CO vibrational frequency of 2095 cm^{-1} (Supplementary Figure 31b) is consistent with our DRIFTS measurement.

On the fully oxidized Pt_8O_{14}/CeO_2 structure, the calculated CO adsorption energy is −27 kJ/mol, which is too low for an appreciable abundance of adsorbed CO. More accurate DFT calculations using the Heyd-Scuseria-Ernzerhof (HSE) hybrid exchange-correlation functional also predict that CO adsorbs weakly on the Pt_8O_{14}/CeO_2 structure. By means of our microkinetic simulation, the predicted CO coverage on the Pt_8O_{14}/CeO_2 is about 10^{-7} at 385 K. Thus, the predicted high CO vibrational frequency (2136 cm^{-1}) and low CO coverage on Pt_8O_{14}/CeO_2 could not be assigned as the signal observed in our DRIFTS spectra. To understand the experimental IR spectra further, more comprehensive DFT calculations have been performed to study CO adsorption energy and the corresponding vibrational frequency on all the possible structures involved in CO oxidation cycle shown in Supplementary Figure 31.

From DRIFTS experiments on Pt-O-Pt/CeO_2 catalyst, there are generally two different CO vibrational frequencies with the values of 2107 and 2081 cm^{-1}, respectively. By considering all possible CO adsorption sites on Pt_8O_x/CeO_2 (x = 13−14) structure involved in CO oxidation cycle by DFT calculations, we found that by removing one O atom from Pt_8O_{14}/CeO_2 to create undercoordinated Pt_8O_{13}/CeO_2 enables CO to adsorb strongly on the top site with an adsorption energy of −146 kJ/mol and a vibrational frequency of 2113 cm^{-1}. The predicted CO coverage is about 0.8 at 385 K by microkinetic simulation, thus can be reasonably assigned as the observed CO adsorption mode (2107 cm^{-1}) in our DRIFTS experiment. CO can adsorb at the bridge site of Pt_8O_{13}/CeO_2 with the calculated CO adsorption energy and vibrational frequency of −153 kJ/mol and 2069 cm^{-1}, respectively. CO can adsorb readily to either the bridge site or top site and the two predicted CO vibrational frequencies with strong CO adsorption strength are consistent with our DRIFTS experiment.

We reiterate that the Pt_8O_{14} cluster model is regarded as a representative structure of our experimental system. In reality, the spectroscopic characterization and experimentally measured activity are averaged over different structures and sizes, subject to their occurrence probability under the reaction conditions[57,58]. Although the design of our experimental system aims to create the exclusive presence of the desired Pt-O-Pt structures, there nevertheless exists some heterogeneity of the cluster structure and size due to the heterogeneous surface properties of the ceria supports comprising of 10–30 nm nanoparticles and non-ideal crystal surfaces. It would be ideal to exhaustively model all observed structures of clusters as a statistical ensemble, acknowledging that they can often adopt a variety of stable and metastable structures during the reaction[57,58], but this is not tractable.

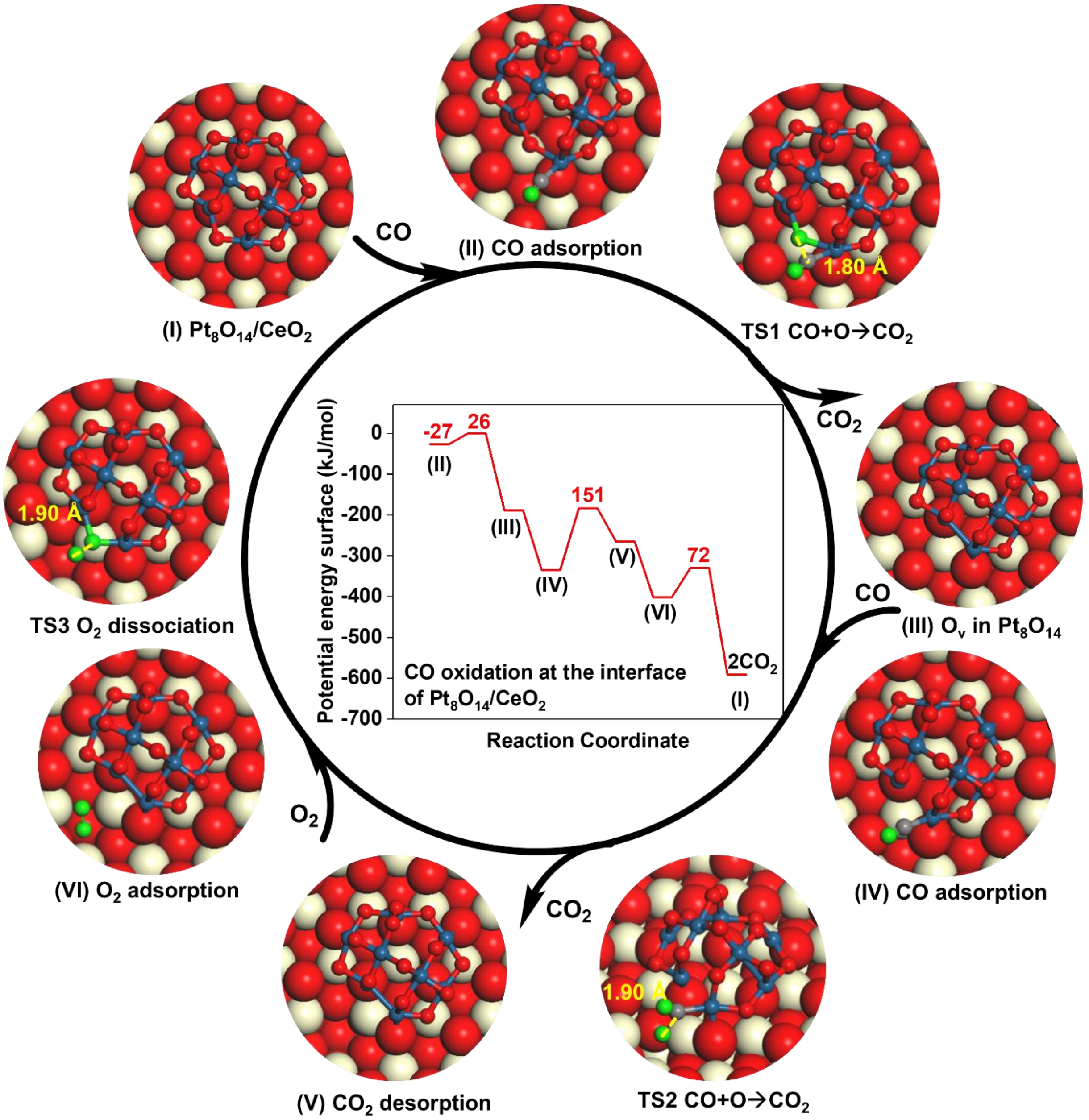

Supplementary Figure 32. Calculated CO oxidation cycle at the platinum-ceria interface of Pt_8O_{14}/CeO_2. The configurations and potential energy surface for CO oxidation cycle at the platinum-ceria interface of Pt_8O_{14}/CeO_2. The reaction barrier and CO adsorption energy are indicated in the potential energy surface in kJ/mol. The bond distance between the two fragments at the transition state (TS) is given in Å. Atom color legend: The small grey and green spheres are C and O atoms involved in CO oxidation, respectively. Corresponding energetics are given in Supplementary Table 4.

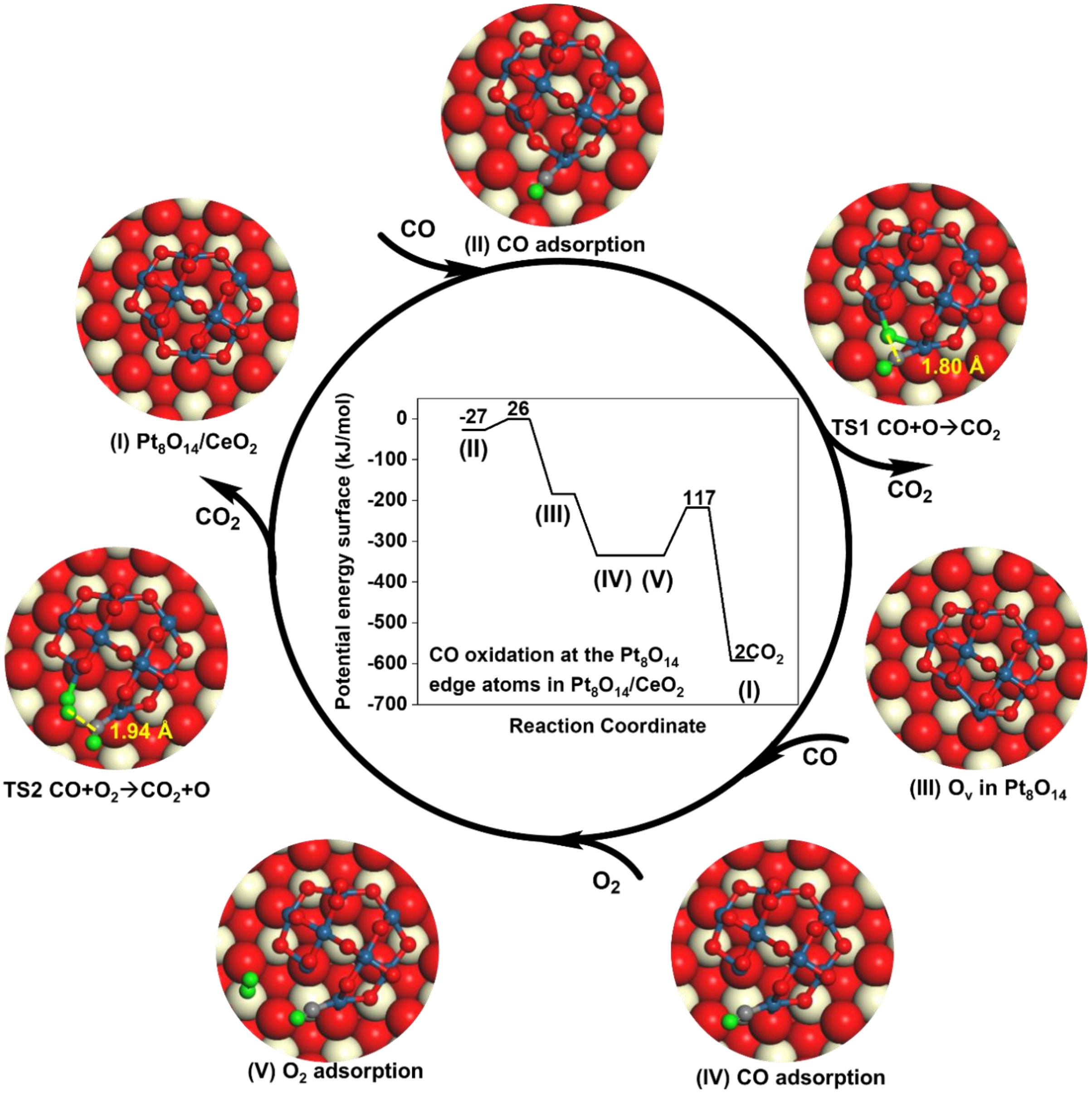

Supplementary Figure 33. Calculated CO oxidation cycle at the Pt_8O_{14} edge atoms in Pt_8O_{14}/CeO_2. The configurations and potential energy surface for CO oxidation cycle at the Pt_8O_{14} edge atoms in Pt_8O_{14}/CeO_2. The reaction barrier and CO adsorption energy are indicated in the potential energy surface in kJ/mol. The bond distance between the two fragments at the transition state (TS) is given in Å. Color legend: The small grey and green spheres are C and O atoms involved in CO oxidation, respectively. Corresponding energetics are given in Supplementary Table 4.

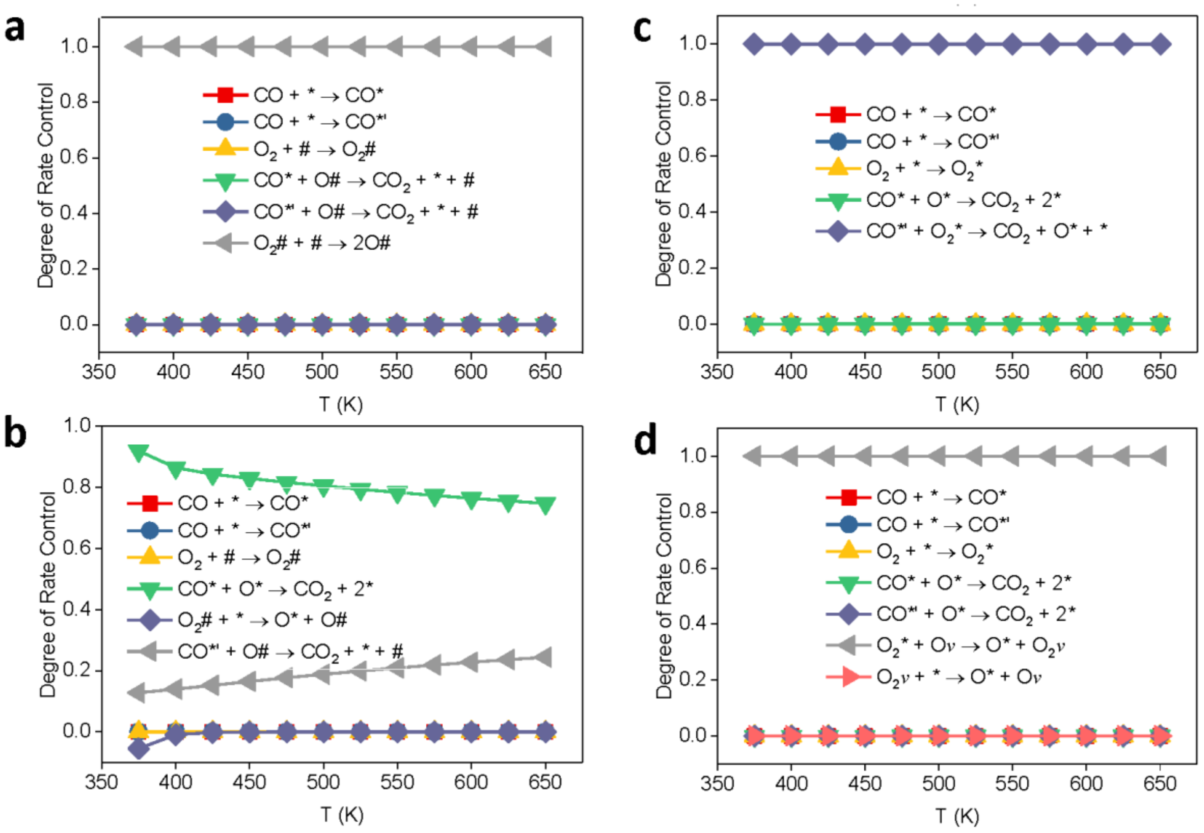

Supplementary Figure 34. Degree of rate control analysis for CO oxidation on the Pt_1/CeO_2 and Pt_8O_{14}/CeO_2. a CO oxidation on Pt_1/CeO_2. **b** CO oxidation at the platinum-ceria interface of Pt_8O_{14}/CeO_2. **c** CO oxidation at the Pt_8O_{14} edge atoms of Pt_8O_{14}/CeO_2. **d** CO oxidation at the Pt-O-Pt site in the Pt_8O_{14} of Pt_8O_{14}/CeO_2. * and # are the active sites on Pt_8O_{14} and CeO_2, respectively. O*v* and O_2*v* denote the O atom and O_2 adsorption neighboring * or O* sites in Pt_8O_{14}, respectively. CO*' denotes CO adsorption after an oxygen vacancy in ceria or Pt_8O_{14} is formed.

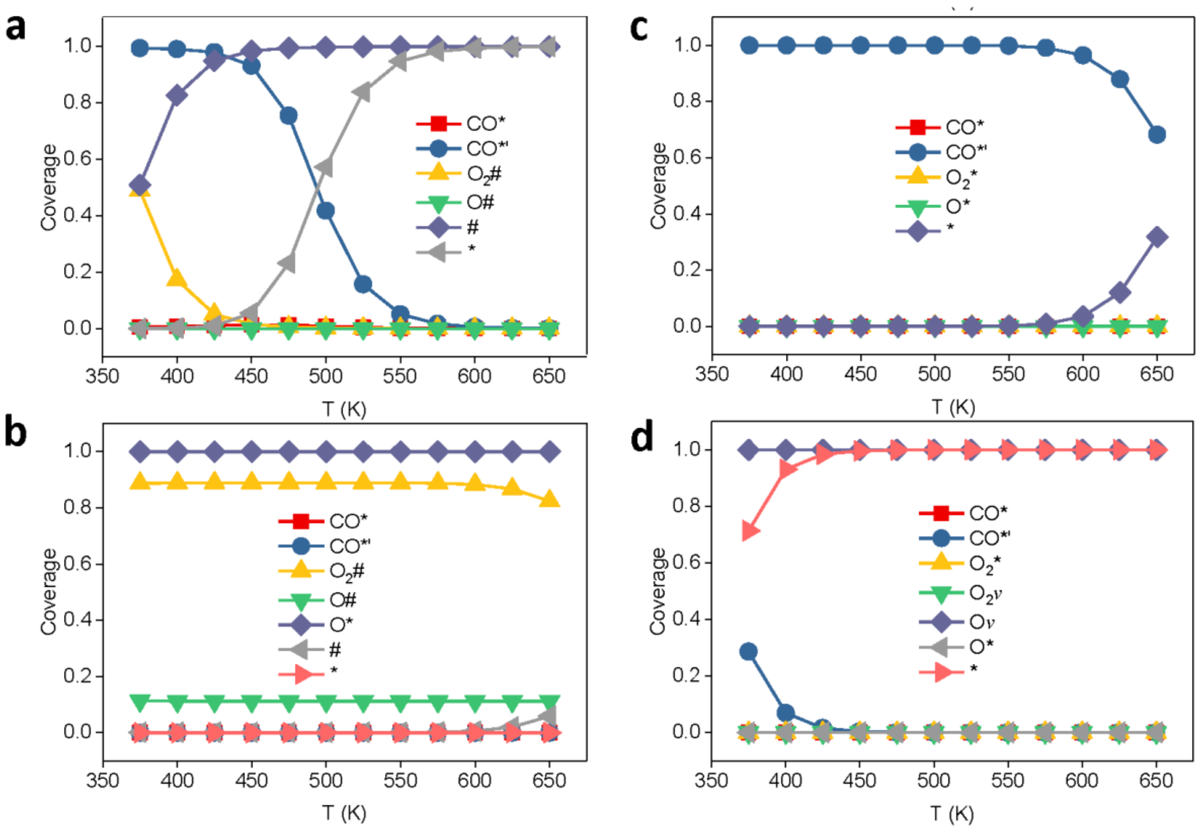

Supplementary Figure 35. Species coverages as a function of temperature for CO oxidation on Pt_1/CeO_2 and Pt_8O_{14}/CeO_2 structures, accordingly to microkinetic simulations. a CO oxidation on Pt_1/CeO_2. **b** CO oxidation at the platinum-ceria interface of Pt_8O_{14}/CeO_2. **c** CO oxidation at the Pt_8O_{14} edge atoms of Pt_8O_{14}/CeO_2. **d** CO oxidation at the Pt-O-Pt site in the Pt_8O_{14} of Pt_8O_{14}/CeO_2. * and # are the active sites on Pt_8O_{14} and CeO_2, respectively. O*v* and O_2*v* are the O atom and O_2 adsorption neighboring * or O* sites in Pt_8O_{14}, respectively. CO*' denotes CO adsorption after an oxygen vacancy in ceria or Pt_8O_{14} is formed.

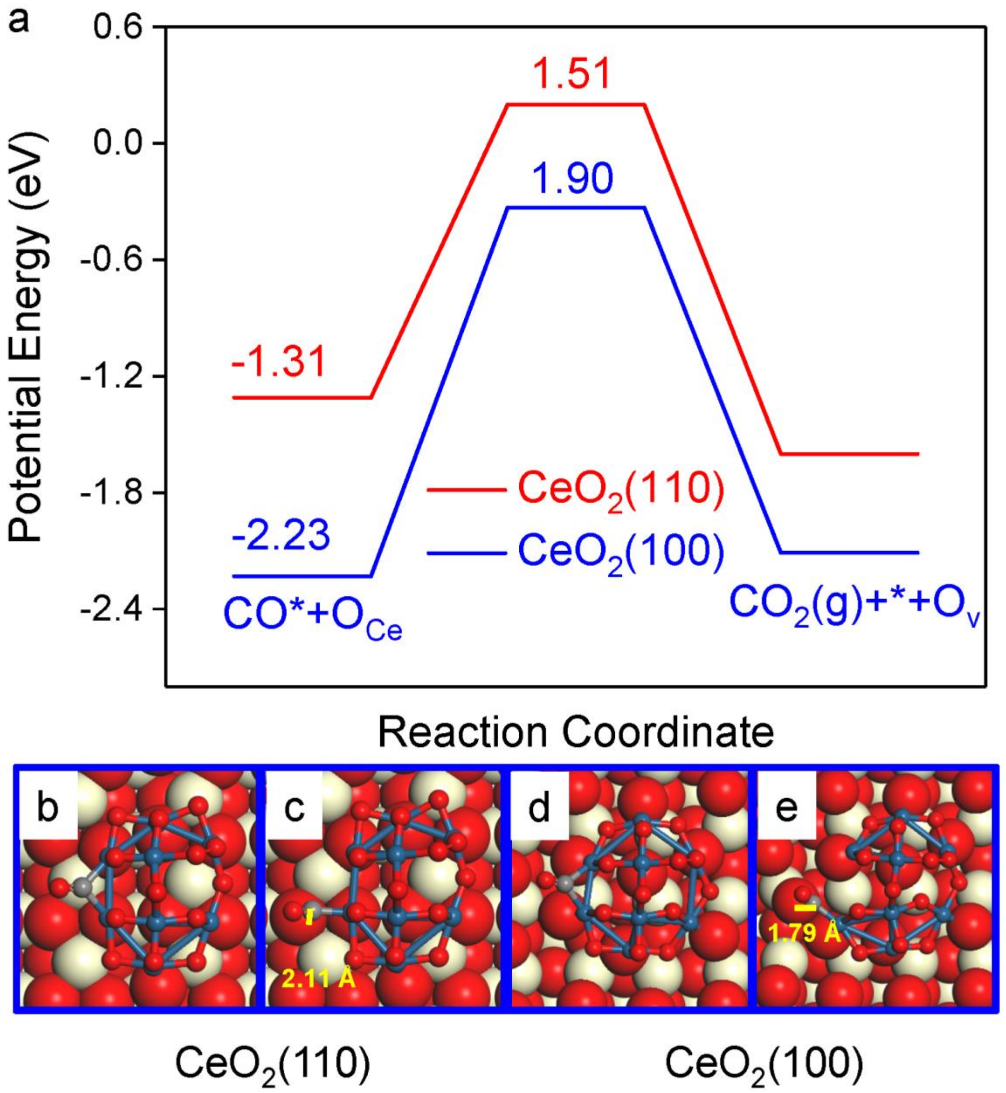

Supplementary Figure 36. CO oxidation on $Pt_8O_{14}/CeO_2(110)$ and $Pt_8O_{14}/CeO_2(100)$. a Calculated potential energy diagram for CO oxidation at the interface of $Pt_8O_{14}/CeO_2(110)$ and $Pt_8O_{14}/CeO_2(100)$ structures. CO adsorption energies and the reaction barriers for CO reacting with lattice oxygen in CeO_2 are indicated in eV. **b** and **d** CO adsorption, **c** and **e** transition state configurations for CO oxidation at the interface of $Pt_8O_{14}/CeO_2(110)$ and $Pt_8O_{14}/CeO_2(100)$ structures, respectively. The distance between the two fragments at CO oxidation transition state is indicated in Å.

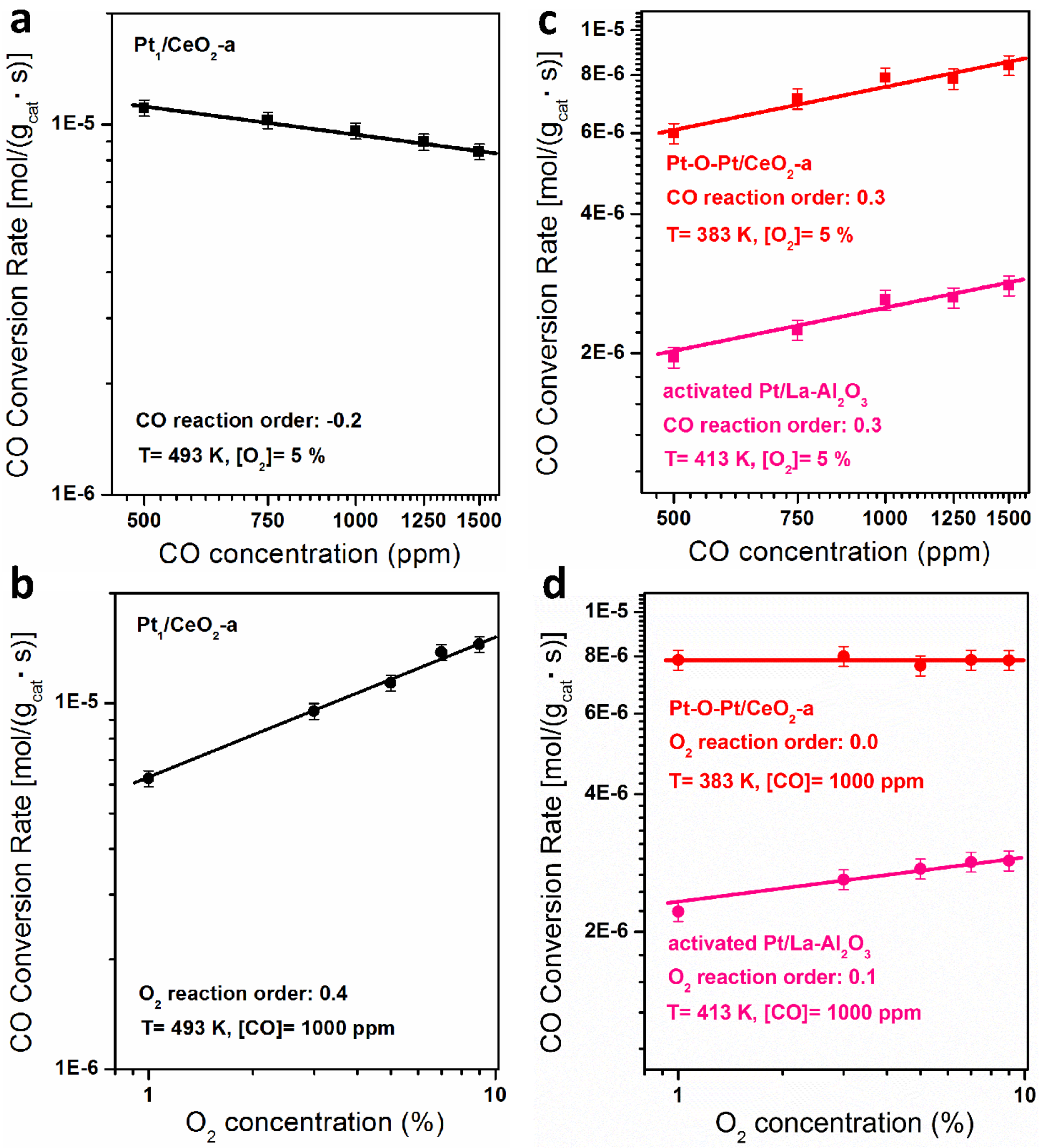

Supplementary Figure 37. Reaction orders for the Pt/CeO_2-a and activated Pt/La-Al_2O_3 catalysts. a CO and **b** O_2 reaction orders for the single-atom Pt_1/CeO_2-a. **c** CO and **d** O_2 reaction orders for Pt-O-Pt/CeO_2 and the activated Pt/La-Al_2O_3 catalysts. The reaction orders were determined at 493 K for Pt_1/CeO_2, 413 K for activated Pt/La-Al_2O_3, and 383 K for Pt-O-Pt/CeO_2-a by adjusting the CO and O_2 concentrations and limiting the overall CO conversion below 20%.

Supplementary Note 12:

The reaction orders for CO and O_2 were measured at 493 K and 383 K for the Pt_1/CeO_2 and Pt-O-Pt/CeO_2 catalysts respectively, for which the temperatures were chosen to stay above the light-

off temperature of each group of catalysts while the overall conversions were below 20 % to minimize mass transfer influences. The reaction order results show that the CO and O species are the most abundant reaction intermediates during the low-temperature CO oxidation reaction. For Pt_1/CeO_2, the reaction orders for CO and O_2 are −0.2 and 0.4 (Supplementary Figure 37a, 37b), respectively. Whereas for Pt-O-Pt/CeO_2, the reaction orders for CO and O_2 are 0.3 and 0.0 (Supplementary Figure 37c, 37d), respectively. We also performed a similar activation protocol to activate a single-atom Pt_1/La-Al_2O_3 catalyst recently reported[4] and managed to convert a portion of the platinum species into a similarly structured active Pt-O-Pt species. On the activated Pt/La-Al_2O_3 catalyst, we observed the reaction orders for CO and O_2 as 0.3 and 0.1 (Supplementary Figure 37c, 37d), respectively, which are consistent with the findings on the Pt-O-Pt/CeO_2 catalysts besides the similar apparent activation energy. These reaction order numbers fall into the typical range that one should expect to see those proposed reaction mechanisms, where the PGM species won't bind CO too strongly and utilize the O from a nearby sites effectively to oxidize CO[59-63].

It is challenging to directly compare the measured reaction orders with the values predicted by microkinetic simulations due to the adsorption energy deviations between experimental data and calculated results[64,65]. For instance, a small variation of CO adsorption energy of 0.15 eV will change the CO reaction order greatly from -0.67 to 0.75 in the water-gas-shift reaction based on DFT calculations[64]. To examine the experimentally measured reaction orders, we have used a global kinetics model in reflection of the theoretical reaction routes to predict the CO oxidation reaction orders on the two groups of catalysts.

In our Pt_1/CeO_2 models, the CO and O_2 molecules will adsorb on distinctive sites. Namely, the CO will adsorb on the single-atom Pt_1, while the O_2 will adsorb and dissociate on CeO_2. A simplified reaction route comprising three key steps is proposed below to capture the essence of the theory predicted mechanism and to relate to classic kinetic expressions:

1) CO adsorption on the single-atom Pt site
2) O_2 adsorption on CeO_{2-x} without competition with CO
3) CO_2 formation from CO and O and desorption

The overall reaction route becomes:

1） $CO + * \underset{k_{-1}}{\overset{k_1}{\rightleftharpoons}} CO^*$

2） $O_2 + 2\# \underset{k_{-2}}{\overset{k_2}{\rightleftharpoons}} 2O^\#（RDS）$

3） $CO^* + O^\# \underset{k_{-3}}{\overset{k_3}{\rightleftharpoons}} CO_2\,(\mathrm{g}) + * + \#$

Since the dissociation of O_2 is rate-determining, then reaction step 1) should reach equilibrium, so:

$$\left[CO^*\right] = \frac{K_1 P_{co}}{1 + K_1 P_{co}}, where\, K_1 = \frac{k_1}{k_{-1}}$$

Reaction step 2) can be treated with the pseudo steady-state hypothesis, where

$$\frac{d\left[O^{\#}\right]}{dt}=k_2P_{o_2}(1-\left[O^{\#}\right])^2-k_{-2}\left[O^{\#}\right]^2-k_3\left[CO^*\right]\left[O^{\#}\right]=0$$

By combining the above two expressions, we obtain:

$$\left[O^{\#}\right]=\frac{2k_2P_{o_2}+k_3\left[CO^*\right]\pm\sqrt{(k_3\left[CO^*\right])^2+4k_2P_{o_2}(k_3\left[CO^*\right]+k_{-2})}}{2(k_2P_{o_2}-k_{-2})}$$

Since the dissociation of O_2 is rate determining, the $[O^{\#}]$ should be a small value. Therefore, $k_2P_{o_2} \ll k_{-2}$ at low O_2 pressure and high O_2 dissociation barrier. Consequently,

$$\left[O^{\#}\right]=\frac{\sqrt{(k_3\left[CO^*\right])^2+4k_2P_{o_2}(k_3\left[CO^*\right]+k_{-2})}-k_3\left[CO^*\right]}{2k_{-2}}$$

The equation for $[O^{\#}]$ can be simplified to:

$$\left[O^{\#}\right]=\sqrt{mP_{O_2}+n}-\frac{k_3\left[CO^*\right]}{2k_{-2}}$$

$$where\ \ m=\frac{K_2\left(k_3\left[CO^*\right]+k_{-2}\right)}{k_{-2}},\left(K_2=\frac{k_2}{k_{-2}}\right)\ and\ \ n=\frac{\left(k_3\left[CO^*\right]\right)^2}{4k_{-2}{}^2}$$

The rate of CO_2 formation can be expressed as:

$$r=k_3\left[CO^*\right]\left[O^*\right]=k_3\sqrt{mP_{o_2}+n}(\frac{K_1P_{co}}{1+K_1P_{co}})-\frac{k_3{}^2}{2k_{-2}}(\frac{K_1P_{co}}{1+K_1P_{co}})^2$$

Ultimately, the observed reaction order of O_2 near 0.5 for Pt_1/CeO_2 is thus feasible for $mP_{o_2} \gg n$ (when [CO*] is very low). The slightly negative reaction order for CO is driven by the input of negative $\frac{K_1P_{co}}{1+K_1P_{co}}$ expressions.

In our Pt-O-Pt/CeO_2 models, CO adsorbs stronger than O_2 on Pt site (Supplementary Table 5). After the formation of O vacancy in Pt-O-Pt ensemble, O_2 adsorbs at the vacancy site (denoted as Pt-[O_v]-Pt ensemble) for dissociation by the oxygen migration step. In the simplified experimental kinetics expression, we therefore propose CO will adsorb on one of the Pt atoms (*) in Pt-O-Pt ensemble, while the O_2 will adsorb on the vacancy in Pt-[O_v]-Pt ensemble, labeled as "σ". The reaction route mainly includes three key steps:

1) CO adsorption on Pt atoms * in the P-O-Pt ensemble
2) O_2 adsorption on the vacancy in Pt-[O_v]-Pt ensemble σ

3) CO_2 formation from CO and the activated O^{σ}, upon the -O-O- migration/activation as the rate determining step

The overall reaction route becomes:

1) $CO + * \underset{k_{-1}}{\overset{k_1}{\rightleftharpoons}} CO^*$

2) $O_2 + \sigma \underset{k_{-2}}{\overset{k_2}{\rightleftharpoons}} O_2^{\sigma}$

3) $CO^* + \frac{1}{2}O_2^{\sigma} \xrightarrow{k_3} CO_2$ （RDS）

By considering steps 1) and 2) in equilibrium, we can get:

$$\left[CO^*\right] = \frac{K_1 P_{co}}{1 + K_1 P_{co}} \quad (K_1 = \frac{k_1}{k_{-1}})$$

$$\left[O_2^{\sigma}\right] = \frac{K_2 P_{o_2}}{1 + K_2 P_{o_2}} \quad (K_2 = \frac{k_2}{k_{-2}})$$

Therefore, the CO oxidation reaction rate can be determined by:

$$r = k_3\left[CO^*\right]\sqrt{\left[O_2^{\sigma}\right]} = k_3 K_1 \sqrt{K_2}\,\frac{P_{co}\sqrt{P_{o_2}}}{(1 + K_1 P_{co})\sqrt{(1 + K_2 P_{o_2})}}$$

Since the oxygen partial pressure is relatively high ([CO]:[O_2] = 1:50), and the oxygen intake to the oxygen removed Pt-[O_V]-Pt is thermodynamically preferred:

$$K_2 P_{o_2} \gg 1 \quad and \quad 1 + K_2 P_{o_2} \approx K_2 P_{o_2}$$

and

$$r = k_3 K_1 \frac{P_{co}}{1 + K_1 P_{co}}$$

Thus, a reaction order close to 0 for O_2 is plausible.

When $K_1 P_{co} \gg 1$, the reaction order for CO can be 0, corresponding to high CO concentration and strong CO adsorption. When $K_1 P_{co} \ll 1$, the reaction order for CO can be 1, which is related to a low CO concentration and weak CO adsorption. While the reacting CO binds to the Pt-O-Pt ensemble with a strong strength at 2081 cm^{-1} (see Fig. 2f and Fig. 3b), and the gaseous CO concentration is reasonably substantial from 500 to 1500 ppm. Therefore, the reaction order for CO of 0.3, between the above mentioned two extremes, is plausible.

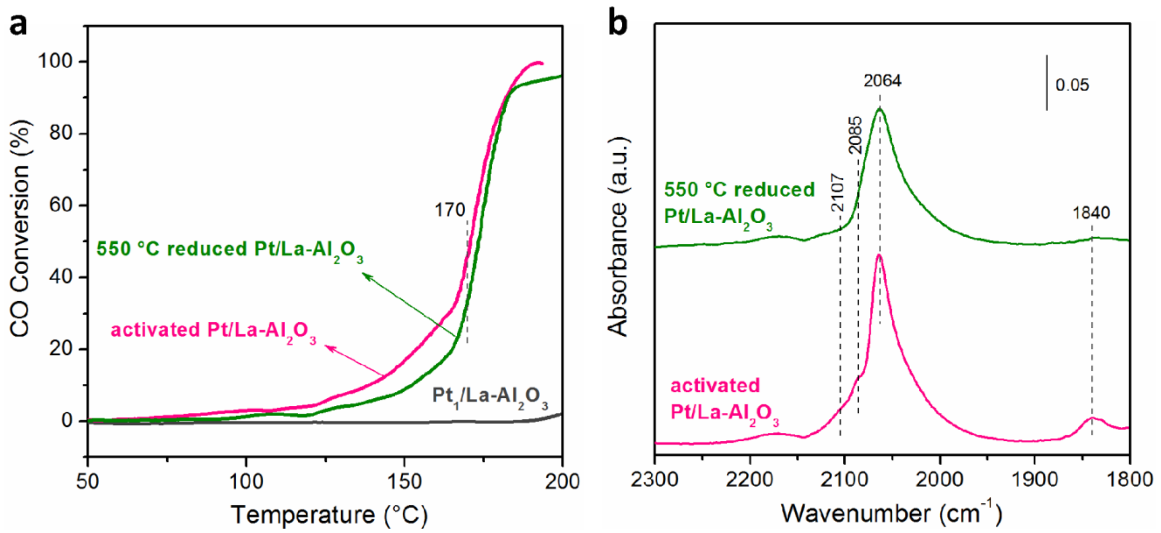

Supplementary Figure 38. CO oxidation performance and *in situ* DRIFTS for the Pt_1/La-Al_2O_3, activated Pt/La-Al_2O_3, and 550 °C reduced Pt/La-Al_2O_3 catalysts. a CO oxidation light-off performance ([CO] = 1000 ppm, [O_2] = 5 %, balanced with N_2 at a contact time of 2,400,000 mL g_{cat}^{-1} $hour^{-1}$) and **b** *in situ* DRIFTS under the CO oxidation conditions (tested at 100 °C with [CO]:[O_2] = 1:50).

Supplementary Note 13:

The data in Supplementary Figure 38a shows the single-atom Pt_1/La-Al_2O_3 has a much inferior light-off activity compared with the activated Pt/La-Al_2O_3 catalyst. The CO adsorption in DRIFTS experiment following the same treatment with reaction tests indicates a formation of Pt-O-Pt like structure (CO adsorption at 2107 and 2085 cm^{-1}) on the activated Pt/La-Al_2O_3 catalyst (Supplementary Figure 38b). However, unlike the ideal case for a few of our Pt-O-Pt/CeO_2 catalysts, the Pt-O-Pt ensemble is not the exclusive platinum species in this activated Pt/La-Al_2O_3 catalyst. As for other Pt species found by in situ DRIFTS, the adsorption at 2064 cm^{-1} can be assigned to CO adsorbed on oxidized Pt particles over 3 nm size, and the adsorption at 1840 cm^{-1} indicates the bridged CO adsorption on metallic Pt particles[66-71]. Next, we tried to passivate the possible Pt-O-Pt structure by H_2 reduction at 550 °C to make the 550 °C reduced Pt/La-Al_2O_3 catalyst. As shown in Supplementary Figure 38b, the CO adsorption signal on the Pt-O-Pt species at 2107 and 2085 cm^{-1} has decreased in the 550 °C reduced Pt/La-Al_2O_3 catalyst and gives rise to the widening IR peak shoulder between 2050 to 2000 cm^{-1}, indicating linear CO adsorption on the formed metallic Pt particles upon severe reduction. Under these conditions, the CO oxidation activity of the 550 °C reduced Pt/La-Al_2O_3 catalyst decreases especially in the low-temperature region of 100-170 °C, further indicating the benefit of the Pt-O-Pt structure in catalyzing the low-temperature CO oxidation.

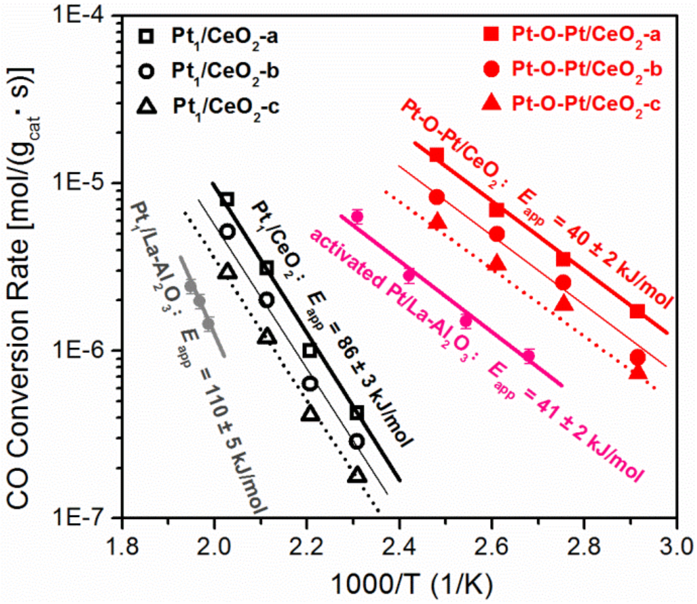

Supplementary Figure 39. Arrhenius-type plot of CO oxidation rates at different temperatures with apparent activation energies (E_{app}) for each catalyst shown.

Supplementary Table 1. Comparison of the CO oxidation rates per platinum atom site (turnover frequency, TOF) for various supported platinum catalysts from literature and in this work.

Reaction tests under H_2O/-OH free conditions					
Species	Sample	Reaction conditions	Temperature (°C)	TOF ($\times 10^{-2}$ s^{-1})	Ref.
Single atoms	Pt/Al_2O_3 + polyhedral ceria (Pt_1/CeO_2 mostly)	[CO] = 1.94 %, [O_2] = 1.29 %, original rate reported at 160 °C	150	0.70[a]	27
	Pt_1/CeO_2	[CO] = 0.4 %, [O_2] = 10 %, original rate reported at ~280 °C	150	0.05 [a]	26
	Pt_1/CeO_2	[CO] = 1 %, [O_2] = 20 %, original rate reported at ~175 °C	150	9.2 [a]	72
	0.18Pt/θ-Al_2O_3	[CO] = 3.7 %, [O_2] = 3.7 %, original rates reported in 200~250 °C	150	0.05[a]	66
	0.2Pt/m-Al_2O_3-H_2	[CO] = 2.5 %, [O_2] = 2.5 %	150	0.3[b]	54
	$Pt(NH_3)_4^{2+}$/KLTL	[CO] = 1 %, [O_2] = 5 %	150	0.38[b]	73
	PtO_x/KLTL			1.2[b]	
	Pt_1/TiO_2	[CO] = 1 %, [O_2] = 1 %, original rate reported at 160 °C	150	1.5	56
	Pt_1/CeO_2-a	[CO] = 0.1 %, [O_2] = 5 %	150	1.7	This work
Nanoparticles dominated	1% Pt/TiO_2 (Pt dispersion ≈ 60 %)	[CO] = 1 %, [O_2] = 1 %, original rate reported at 160 °C	150	0.4[a]	56
	Commercial Pt/Al_2O_3 (Pt dispersion= 41 %)	[CO] = 2.5 %, [O_2] = 2.5 %	150	35[b]	54
	Pt/Al_2O_3 (19 nm, Pt dispersion=1.5 %)	[CO] = 1 %, [O_2] = 10 %	150	22[b]	67
	2 % Pt/CeO_2 (Pt dispersion=57 %, originally named as Pt(2)/(800 C)CeO_2)	[CO] = 0.1 %, [O_2] = 10 %, original rate reported at 190 °C	150	2.3[a]	74
	1.3% PtO_x/CeO_2 NWs-350 (less O) (0.5±0.1 nm, Pt dispersion=68 %)	[CO] = 13 %, [O_2] = 33 %, original rate reported at 40-80 °C	150	66[c]	51
Pt-O-Pt/CeO_2	Pt-O-Pt /CeO_2-a [e] (Pt dispersion=100 %)	[CO] = 0.1 %, [O_2] = 5 %	150	197	This work

Reaction tests under H_2O/-OH enriched environment					
Species	Sample	Reaction conditions	Temperature (°C)	TOF ($\times 10^{-2}$ s^{-1})	Ref.
Single atoms	0.4Pt-Ba/La-Al_2O_3	[CO] = 5000 ppm, [HCs] = 500 ppm, [O_2] = 1%, [H_2O] = 5 %, original rate reported at 200 °C	50	0.005[a]	4
	Pt_1/CeO_2	[CO] = 1 %, [O_2] = 20 %, [H_2O] = 4 %, original rate reported at 110 °C	50	1.2[a]	72
	Pt_1/CeO_2-hydrated (Originally named as Pt/CeO_2_S)	[CO] = 0.4 %, [O_2] = 10 %, original rate reported at ~100 °C	50	0.007[a]	26
		[CO] = 0.4 %, [O_2] = 10 %, [H_2O] = 10 %	50	7.7[d]	
Pt-O-Pt/CeO_2	Pt-O-Pt /CeO_2-a [e] (Pt dispersion=100 %	[CO] = 0.1 %, [O_2] = 5 %, [H_2O] = 3 %	50	60[d]	This work
	Activated aged Pt-O-Pt /CeO_2-a [f]	[CO] = 0.1 %, [O_2] = 5 %, [H_2O] = 3 %	50	51[d]	

*Note: [a] Calculated based on the known/calculated E_{app} and TOFs read from figures or reported by authors. [b] Shown by the authors. [c] E_{app} was adopted from our measurement of 40 ± 2 kJ/mol, since they did not report E_{app} at high temperatures up to 150 °C, and they may have created a portion of the similar sites as we did in this work. [d] Calculated based on flow rate, CO concentration, light-off conversion and loading amount. [e] The Pt-O-Pt/CeO_2-b and Pt-O-Pt/CeO_2-c also exhibited the same activity. [f] Hydrothermal aging condition: 750 °C for 20 hrs, 10 % H_2O in air.

Supplementary Table 2. Bader charge analysis. Charge state of Pt atoms in the Pt_1/CeO_2, Pt_8O_{14}/CeO_2, PtO, and PtO_2 structures are given.

Structure	Bader Charge of Pt atoms
Pt_1/CeO_2	+1.51
Pt_8O_{14}/CeO_2	$+1.23 \pm 0.21^{\alpha}$
PtO (bulk phase with orthorhombic P_{nma} structure)[75]	+0.81
PtO_2 (bulk phase with orthorhombic P_{nnm} structure)[75]	+1.73

$^{\alpha}$ The average Bader charge is given for Pt_8O_{14}/CeO_2.

Supplementary Table 3. Fitting parameters of the curve fitted k^2-weighted EXAFS analysis of the Pt_1/CeO_2 and Pt-O-Pt/CeO_2 catalysts*.

Sample	Shell	N	R(Å)	ΔE_0 (eV)	R-factor
Pt_1/CeO_2-a	Pt-O	6.02	2.00	8.49	0.02
Pt-O-Pt /CeO_2-a	Pt-O	3.99	1.99	8.43	0.02
Pt_1/CeO_2-b	Pt-O	6.07	1.99	8.47	0.02
Pt-O-Pt/CeO_2-b	Pt-O	3.99	1.99	8.47	0.02
Pt_1/CeO_2-c	Pt-O	5.72	1.99	8.54	0.02
Pt-O-Pt/CeO_2-c	Pt-O	3.96	1.99	8.47	0.02

*Direct Pt-Pt contact as the 1st shell Pt-Pt coordination does not exist in the above listed reaction-spent catalysts. Amplitude reduction factor: S_0^2: 0.79; N, coordination number; R, distance between absorber and backscattered atoms; ΔE_0, inner potential correction; R-factor, closeness of the fit, if < 0.05, consistent with broadly correct models. Estimated error: N: ± 20 %, R: ± 0.03; ΔE_0: ± 25 %.

Supplementary Table 4. Calculated energetics for CO oxidation. Calculated adsorption energies of CO (E_{CO} and $E_{CO'}$) and O_2 (E_{O2}), as well as forward (E_f) and backward (E_b) activation energies for CO oxidation on the Pt_1/CeO_2, at the interface and Pt_8O_{14} edge atoms of Pt_8O_{14}/CeO_2, and at Pt-O-Pt in Pt_8O_{14} on ceria. All energies are reported in kJ/mol.

CO oxidation on Pt_1/CeO_2

Reaction step[a]	CO*	CO* + O# → CO_2 + * + #		CO'*	CO'* + O# → CO_2 + * + #		O_2#	O_2# + # → 2O#	
Energy (kJ/mol)	E_{CO}	E_f	E_b	$E_{CO'}$	E_f	E_b	E_{O2}	E_f	E_b
	−88	11	256	−103	105	207	−65	150	138

CO oxidation at the interface of Pt_8O_{14}/CeO_2

Reaction step	CO*	CO* + O* → CO_2 + 2*		CO'*	CO'* + O# → CO_2 + * + #		O_2#	O_2# + * → O# + O*	
Energy (kJ/mol)	E_{CO}	E_f	E_b	$E_{CO'}$	E_f	E_b	E_{O2}	E_f	E_b
	−27	26	188	−146	151	81	−137	72	261

CO oxidation at the Pt_8O_{14} edge atoms in Pt_8O_{14}/CeO_2

Reaction step	CO*	CO* + O* → CO_2 + 2*		CO'*	O_2*	CO'* + O_2* → CO_2 + O* + * [b]	
Energy (kJ/mol)	E_{CO}	E_f	E_b	$E_{CO'}$	E_{O2}	E_f	E_b
	−27	26	188	−146	−1	117	373

CO oxidation at the Pt-O-Pt catalytic unit in Pt_8O_{14} on ceria

Reaction step	CO*	CO* + O* → CO_2 + 2*		O_2*	O_2* + O*v* → O* + O_2*v*		CO*'	CO*' + O* → CO_2 + 2*		O_2*v* + * → O*v* + O*	
Energy (kJ/mol)	E_{CO}	E_f	E_b	E_{O2}	E_f	E_b	$E_{CO'}$	E_f	E_b	E_f	E_b
	−27	26	188	−36	103	132	−73	37	159	77	220

[a] The * and # denote the active site on Pt_1 and CeO_2, respectively. CO* and CO'* denote CO adsorption on the pristine Pt_1/CeO_2 and Pt_8O_{14}/CeO_2 with one oxygen vacancy in ceria, respectively. This notation is used throughout the supplementary information. [b] Note, this step corresponds to CO_{Pt} + O_2 → CO_2 + O_{Pt} in the main text. *v* is the adsorption site that is neighboring * on Pt_8O_{14}.

Supplementary Table 5. CO and O_2 adsorption energies. $Pt_1/CeO_{2\text{-}x}$ and Pt_8O_{13}/CeO_2 indicate the structures after the removal of one oxygen atom by the first *CO + *O → *CO_2 reaction step on Pt_1/CeO_2 and Pt_8O_{14}/CeO_2, respectively. All the energies are in kJ/mol.

Structures	E_{CO}	E_{O2}
Pt_1/CeO_2	−88	−2
$Pt_1/CeO_{2\text{-}x}$	−104	−15
Pt_8O_{14}/CeO_2	−27 (top site)	−2
Pt_8O_{13}/CeO_2	−146 (top site)	−1

Supplementary References

1. Takeguchi, T. et al. Determination of dispersion of precious metals on CeO_2-containing supports. *Appl. Catal. A* **293**, 91-96 (2005).
2. Jeong, H. et al. Fully dispersed Rh ensemble catalyst to enhance low-temperature activity. *J. Am. Chem. Soc.* **140**, 9558-9565 (2018).
3. Yoshida, H. et al. Redox dynamics of Pd supported on CeO_2–ZrO_2 during oxygen storage/release cycles analyzed by time-resolved in situ reflectance spectroscopy. *J. Phys. Chem. C* **122**, 28173-28181 (2018).
4. Wang, H. et al. Single-site Pt/La-Al_2O_3 stabilized by barium as an active and stable catalyst in purifying CO and C_3H_6 emissions. *Appl. Catal. B* **244**, 327-339 (2019).
5. Zhao, M., Shen M., Wang J. Effect of surface area and bulk structure on oxygen storage capacity of $Ce_{0.67}Zr_{0.33}O_2$. *J. Catal.* **248**, 258-267 (2007).
6. Vilhelmsen, L. B., Hammer B. A genetic algorithm for first principles global structure optimization of supported nano structures. *J. Chem. Phys.* **141**, 044711 (2014).
7. Chase Jr, M. et al. JANAF THERMOCHEMICAL TABLES - 3RD EDITION .1. AL-CO. (Ref. Data). *J. Phys. Chem.* **14**, 1-926 (1985).
8. Filot, I. A. W. et al. First-principles-based microkinetics simulations of synthesis gas conversion on a stepped rhodium surface. *ACS Catal.* **5**, 5453-5467 (2015).
9. Niton, P., Zywocinski A., Fialkowski M., Holyst R. A "nano-windmill" driven by a flux of water vapour: a comparison to the rotating ATPase. *Nanoscale* **5**, 9732-9738 (2013).
10. Stegelmann, C., Andreasen A., Campbell C. T. Degree of rate control: how much the energies of intermediates and transition states control rates. *J. Am. Chem. Soc.* **131**, 8077-8082 (2009).
11. Romeo, M. et al. XPS study of the reduction of cerium dioxide. *Surf. Interface Anal.* **20**, 508-512 (1993).
12. Kato, S. et al. Quantitative depth profiling of Ce^{3+} in Pt/CeO_2 by *in situ* high-energy XPS in a hydrogen atmosphere. *Phys. Chem. Chem. Phys.* **17**, 5078-5083 (2015).
13. Peng, R. et al. Size effect of Pt nanoparticles on the catalytic oxidation of toluene over Pt/CeO_2 catalysts. *Appl. Catal. B* **220**, 462-470 (2018).
14. Chen, A. et al. Structure of the catalytically active copper–ceria interfacial perimeter. *Nat. Catal.* **2**, 334–341 (2019).
15. Stadnichenko, A. I. et al. Study of active surface centers of Pt/CeO_2 catalysts prepared using radio-frequency plasma sputtering technique. *Surf. Sci.* **679**, 273-283 (2019).
16. Monai, M. et al. Phosphorus poisoning during wet oxidation of methane over Pd@CeO_2/graphite model catalysts. *Appl. Catal. B* **197**, 271-279 (2016).
17. Beran, J. et al. RHEED and XPS study of cerium interaction with SnO_2 (110) surface. *Ceram. Int.* **40**, 323-329 (2014).
18. Slavinskaya, E. M. et al. Transformation of a Pt-CeO_2 mechanical mixture of pulsed-laser-ablated nanoparticles to a highly active catalyst for carbon monoxide oxidation. *ChemCatChem* **10**, 2232-2247 (2018).
19. Fierro, J. L. G., Palacios J. M., Tomas F. An anarlytical SEM and XPS study of platinum-rhodium gauzes used in high pressure ammonia burners. *Surf. Interface Anal.* **13**, 25-32 (1988).
20. Dauscher, A. et al. Characterization by XPS and XAS of supported Pt/TiO_2-CeO_2 catalysts. *Surf. Interface Anal.* **16**, 341-346 (1990).
21. Svintsitskiy, D. A. et al. Highly oxidized platinum nanoparticles prepared through radio-frequency sputtering: Thermal stability and reaction probability towards CO. *ChemPhysChem* **16**, 3318-3324 (2015).
22. Wang, X., Kang Q., Li D. Low-temperature catalytic combustion of chlorobenzene over MnO_x–CeO_2 mixed oxide catalysts. *Catal. Commun.* **9**, 2158-2162 (2008).
23. Mullins, D. R. et al. Water dissociation on CeO_2(100) and CeO_2(111) thin films. *J. Phys. Chem. C* **116**, 19419-19428 (2012).

24. Trovarelli, A. Catalytic properties of ceria and CeO_2-containing materials. *Catal. Rev.* **38**, 439-520 (1996).
25. Kopelent, R. et al. Catalytically active and spectator Ce^{3+} in ceria-supported metal catalysts. *Angew. Chem. Int. Ed.* **54**, 8728-8731 (2015).
26. Nie, L. et al. Activation of surface lattice oxygen in single-atom Pt/CeO_2 for low-temperature CO oxidation. *Science* **358**, 1419-1423 (2017).
27. Jones, J. et al. Thermally stable single-atom platinum-on-ceria catalysts via atom trapping. *Science* **353**, 150-154 (2016).
28. Yang, M. et al. A common single-site Pt(II)-$O(OH)_x$- species stabilized by sodium on "active" and "inert" supports catalyzes the water-gas shift reaction. *J. Am. Chem. Soc.* **137**, 3470-3473 (2015).
29. Henderson, M. A. et al. Redox properties of water on the oxidized and reduced surfaces of CeO_2(111). *Surf. Sci.* **526**, 1–18 (2003).
30. Pozdnyakova-Tellinger, O. et al. Surface water-assisted preferential CO oxidation on Pt/CeO_2 catalyst. *J. Phys. Chem. C* **111**, 5426-5431 (2007).
31. Saavedra, J. et al. The critical role of water at the gold-titania interface in catalytic CO oxidation. *Science* **345**, 1599-1602 (2014).
32. Lou, Y., Liu J. CO oxidation on metal oxide supported single Pt atoms: The role of the support. *Ind. Eng. Chem. Res.* **56**, 6916-6925 (2017).
33. Holmgren, A., Andersson B., Duprez D. Interactions of CO with Pt/ceria catalysts. *Appl. Catal. B* **22**, 215–230 (1999).
34. Jacobs, G. Low temperature water–gas shift: in situ DRIFTS-reaction study of ceria surface area on the evolution of formates on Pt/CeO_2 fuel processing catalysts for fuel cell applications. *Appl. Catal. A* **252**, 107-118 (2003).
35. Yang, H. et al. Regeneration of SO_2-poisoned diesel oxidation Pd/CeO_2 catalyst. *Catal. Commun.* **36**, 5-9 (2013).
36. Fronzi, M. et al. Stability and morphology of cerium oxide surfaces in an oxidizing environment: A first-principles investigation. *J. Chem. Phys.* **131**, 104701 (2009).
37. Mao, M. et al. Metal support interaction in Pt nanoparticles partially confined in the mesopores of microsized mesoporous CeO_2 for highly efficient purification of volatile organic compounds. *ACS Catal.* **6**, 418−427 (2016).
38. Bruix, A. et al. Effects of deposited Pt particles on the reducibility of CeO_2(111). *Phys. Chem. Chem. Phys.* **13**, 11384-11392 (2011).
39. Wu, T. et al. Investigation of the redispersion of Pt nanoparticles on polyhedral ceria nanoparticles. *J. Phys. Chem. Lett.* **5**, 2479-2483 (2014).
40. Gao, Y., Wang W., Chang S., Huang W. Morphology effect of CeO_2 support in the preparation, metal-support interaction, and catalytic performance of Pt/CeO_2 catalysts. *ChemCatChem* **5**, 3610-3620 (2013).
41. Huang, W., Gao Y. Morphology-dependent surface chemistry and catalysis of CeO_2 nanocrystals. *Catal. Sci. Technol.* **4**, 3772-3784 (2014).
42. Vayssilov, G. N. et al. Support nanostructure boosts oxygen transfer to catalytically active platinum nanoparticles. *Nat. Mater.* **10**, 310-315 (2011).
43. Sk, M. A. et al. Oxygen vacancies in self-assemblies of ceria nanoparticles. *J. Mater. Chem. A* **2**, 18329-18338 (2014).
44. Wang, Z. L., Feng X. Polyhedral shapes of CeO_2 nanoparticles. *J. Phys. Chem. B* **107**, 13563-13566 (2003).
45. Zang, C., Zhang X., Hu S., Chen F. The role of exposed facets in the Fenton-like reactivity of CeO_2 nanocrystal to the Orange II. *Appl. Catal. B* **216**, 106-113 (2017).
46. Migani, A. et al. Greatly facilitated oxygen vacancy formation in ceria nanocrystallites. *Chem. Commun.Chem.* **46**, 5936-5938 (2010).

47. Vayssilov, G. N., Migani A., Neyman K. Density functional modeling of the interactions of platinum clusters with CeO_2 nanoparticles of different size. *J. Phys. Chem. C* **115**, 16081-16086 (2011).
48. Lykhach, Y. et al. Redox-mediated conversion of atomically dispersed platinum to sub-nanometer particles. *J. Mater. Chem. A* **5**, 9250-9261 (2017).
49. Ravel, B. Composing complex EXAFS problems with severe information constraints. *J. Phys.: Conf. Ser.* **190**, 012026 (2009).
50. Range, K.-J., Klement F. R., Heyns A. M. β-PtO_2: High pressure synthesis of single crystals and structure refinement. *Mater. Res. Bull.* **22**, 1541-1547 (1987).
51. Ke, J. et al. Strong Local Coordination Structure Effects on Subnanometer PtO_x Clusters over CeO_2 Nanowires Probed by Low-Temperature CO Oxidation. *ACS Catal.* **5**, 5164-5173 (2015).
52. Dessal, C. et al. Dynamics of Single Pt Atoms on Alumina during CO Oxidation Monitored by Operando X-ray and Infrared Spectroscopies. *ACS Catal.* **9**, 5752-5759 (2019).
53. Ganzler, A. M. et al. Tuning the structure of platinum particles on ceria in situ for enhancing the catalytic performance of exhaust gas catalysts. *Angew. Chem. Int. Ed.* **56**, 13078-13082 (2017).
54. Zhang, Z. et al. Thermally stable single atom Pt/m-Al_2O_3 for selective hydrogenation and CO oxidation. *Nat. Commun.* **8**, 16100 (2017).
55. Ding, K. et al. Identification of active sites in CO oxidation and water-gas shift over supported Pt catalysts. *Science* **350**, 189-192 (2015).
56. DeRita, L. et al. Catalyst architecture for stable single atom dispersion enables site-specific spectroscopic and reactivity measurements of CO adsorbed to Pt atoms, oxidized Pt clusters, and metallic Pt clusters on TiO_2. *J. Am. Chem. Soc.* **139**, 14150-14165 (2017).
57. Sun, G., Sautet P. Metastable structures in cluster catalysis from First-principles: Structural ensemble in reaction conditions and metastability triggered reactivity. *J. Am. Chem. Soc.* **140**, 2812-2820 (2018).
58. Zhai, H., Alexandrova A. N. Fluxionality of catalytic clusters: When it matters and how to address it. *ACS Catal.* **7**, 1905-1911 (2017).
59. Nibbelke, R. H., Campman M. A. J., Hoebink J. H. B. J., Marin G. B. Kinetic study of the CO oxidation over Pt/r-Al_2O_3 and Pt/Rh/CeO_2/r-Al_2O_3 in the presence of H_2O and CO_2. *J. Catal.* **171**, 358-373 (1997).
60. Yao, H. C., Yao Y. F. Y. Ceria in automotive exhaust catalysts: I. Oxygen storage. *J. Catal.* **86**, 254-265 (1984).
61. Bunluesin, T., Cordatos H., Gorte R. J. Study of CO oxidation kinetics on Rh/ceria. *J. Catal.* **157**, 222-226 (1995).
62. Yao, Y. F. Y. The oxidation of CO and hydrocarbons over noble metal catalysts. *J. Catal.* **87**, 152-162 (1984).
63. Liu, H.-H. et al. Oxygen vacancy promoted CO oxidation over Pt/CeO_2 catalysts: A reaction at Pt–CeO_2 interface. *Appl. Surf. Sci.* **314**, 725-734 (2014).
64. Bukowski, B. C., Bates J. S., Gounder R., Greeley J. First principles, microkinetic, and experimental analysis of Lewis acid site speciation during ethanol dehydration on Sn-Beta zeolites. *J. Catal.* **365**, 261-276 (2018).
65. Zhao, Z.-J. et al. Importance of metal-oxide interfaces in heterogeneous catalysis: A combined DFT, microkinetic, and experimental study of water-gas shift on Au/MgO. *J. Catal.* **345**, 157-169 (2017).
66. Moses-DeBusk, M. et al. CO oxidation on supported single Pt atoms: experimental and ab initio density functional studies of CO interaction with Pt atom on theta-Al_2O_3(010) surface. *J. Am. Chem. Soc.* **135**, 12634-12645 (2013).
67. Kale, M. J., Christopher P. Utilizing quantitative in situ FTIR spectroscopy to identify well-coordinated Pt atoms as the active site for CO oxidation on Al_2O_3-supported Pt catalysts. *ACS Catal.* **6**, 5599-5609 (2016).

68. Oertzen, A. v., Rotermund H. H., Nettesheim S. Diffusion of carbon monoxide and oxygen on Pt(110): experiments performed with the PEEM. *Surf. Sci.* **311**, 322-330 (1994).
69. Primet, M., Basset J. M., Mathieu M. V., Prettre M. Infrared study of CO adsorbed on $PtAl_2O_3$.A method for determining metal-adsorbate interactions. *J. Catal.* **29**, 213-223 (1973).
70. Haaland, D. M., Williams F. L. Simultaneous measurement of CO oxidation rate and surface coverage on $PtAl_2O_3$ using infrared spectroscopy: Rate hysteresis and CO island formation. *J. Catal.* **76**, 450-465 (1982).
71. Primet, M. Electronic transfer and ligand effects in the infrared spectra of adsorbed carbon monoxide. *J. Catal.* **88**, 273-282 (1984).
72. Wang, C. et al. Water-mediated Mars–Van Krevelen mechanism for CO oxidation on ceria-supported single-atom Pt_1 Catalyst. *ACS Catal.* **7**, 887-891 (2016).
73. Kistler, J. D. et al. A single-site platinum CO oxidation catalyst in zeolite KLTL: microscopic and spectroscopic determination of the locations of the platinum atoms. *Angew. Chem. Int. Ed.* **53**, 8904-8907 (2014).
74. Lee, J. et al. Influence of the defect concentration of ceria on the Pt dispersion and the CO oxidation activity of Pt/CeO_2. *J. Phys. Chem. C* **122**, 4972-4983 (2018).
75. Nomiyama, R. K., Piotrowski M. J., Da Silva J. L. F. Bulk structures of PtO and PtO_2 from density functional calculations. *Phys. Rev. B* **84**, (2011).
